# Synthesis of Aryl Propionamide Scaffold Containing a Pentafluorosulfanyl Moiety as SARMs

**DOI:** 10.3390/molecules24234227

**Published:** 2019-11-20

**Authors:** Pingxuan Shao, Yan Zhou, Dehua Yang, Ming-Wei Wang, Wei Lu, Jiyu Jin

**Affiliations:** 1 Shanghai Engineering Research Center of Molecular Therapeutics and New Drug Development, School of Chemistry and Molecular Engineering, East China Normal University, 3663 North Zhongshan Road, Shanghai 200062, China; 51174300069@stu.ecnu.edu.cn (P.S.); wlu@chem.ecnu.edu.cn (W.L.); 2 The National Center for Drug Screening and the CAS Key Laboratory of Receptor Research, Shanghai Institute of Materia Medica, Chinese Academy of Sciences (CAS), 189 Guo Shou Jing Road, Shanghai 200062, China; yzhou@simm.ac.cn (Y.Z.); dhyang@simm.ac.cn (D.Y.)

**Keywords:** pentafluorosulfanyl, aryl propionamide, androgen receptor, agonist, SARMs

## Abstract

The pentafluorosulfane (SF_5_) group, as a more electronegative bioisostere than the trifluoromethyl (CF_3_) group, has been gaining greater attention and increasingly reported usage in medicinal chemistry. Ostarine is the selective androgen receptor modulators (SARMs) containing a CF_3_ group in clinical trial III. In this study, 21 ostarine derivatives for replacing the CF_3_ group with SF_5_ substituents were synthesized. Some SF_5_-derivatives showed androgen receptor (AR) agonistic activities in vitro. The results pointed to the potential of using this scaffold to develop new AR agonists.

## 1. Introduction

Fluorine has been used widely in drug design and development, and 20–25% of pharmaceuticals contain a fluorine atom at present [1]. It is known that the existence of fluorine can influence electrostatic interactions and hydrogen bonding of a ligand [2]. Besides, the introduction of fluorine into a compound can change its lipophilicity, pKa, and metabolic stability [1]. Direct fluorination and addition of fluorinated functional groups are common methods to incorporate fluorine. The most common example of functional groups is trifluoromethyl (CF_3_).

The pentafluorosulfanyl (SF_5_) group, not only a bioisostere of the CF_3_ group, has been gaining greater attention and increasingly reported usage in medicinal chemistry according to the literature [3,4,5]. Some of the special properties of SF_5_ have been described, such as chemical and hydrolytically stability [5], steric demand and symmetry (volume of SF_5_ is slightly less than t-butyl and greater than CF_3_), electron-withdrawing effect [6,7], electronegativity (SF_5_ = 3.65, CF_3_ = 3.36), and nontoxicity of its degradation products [3]. However, the synthetic accessibility of the SF_5_ group has limited its use. Access to SF_5_-substituted benzenes is primarily accomplished through two methods, namely, treatment of thiophenols with fluorine gas and chlorofluorination of dibenzyl disulfides to give chlorotetrafluorosulfanyl benzenes (ArSF_4_Cl), which are, in turn, fluorinated to SF_5_ analogs [7,8]. The shortcomings of these methods may include low yield and reaction safety. SF_5_-bearing building blocks are attractive due to their reported bioactivities [9].

**Figure 1:**
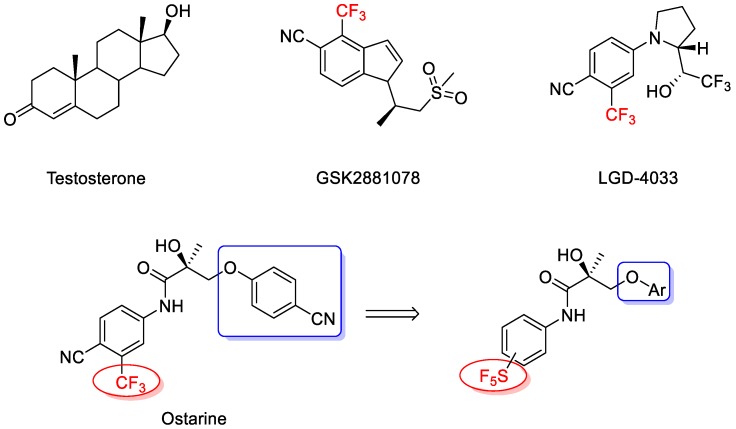
Structures of testosterone and selected SARMs (selective androgen receptor modulators).

Androgen exerts its action via the androgen receptor (AR). Upon activation, AR translocates to the nucleus to mediate both androgenic and anabolic effects. Selective androgen receptor modulators (SARMs) bind AR and display tissue-selective activation of androgenic signaling [10,11,12]. Several nonsteroidal SARMs, such as ostarine [13,14], GSK2881078 [15], and LGD4033 [16], were developed for clinical trials (Figure 1). Among them, ostarine was shown to improve muscle functions in patients with cancer cachexia [17].

It appears that the strong electron-withdrawing effect of trifluoromethyl in SARMs plays a key role in AR binding [18,19,20]. Since none of the reported SARMs bears SF_5_ moiety, we decided to study the possibility and effect of replacing the CF_3_ group with SF_5_ substituents.

## 2. Results and Discussion

### 2.1. Chemistry

**Scheme 1:**
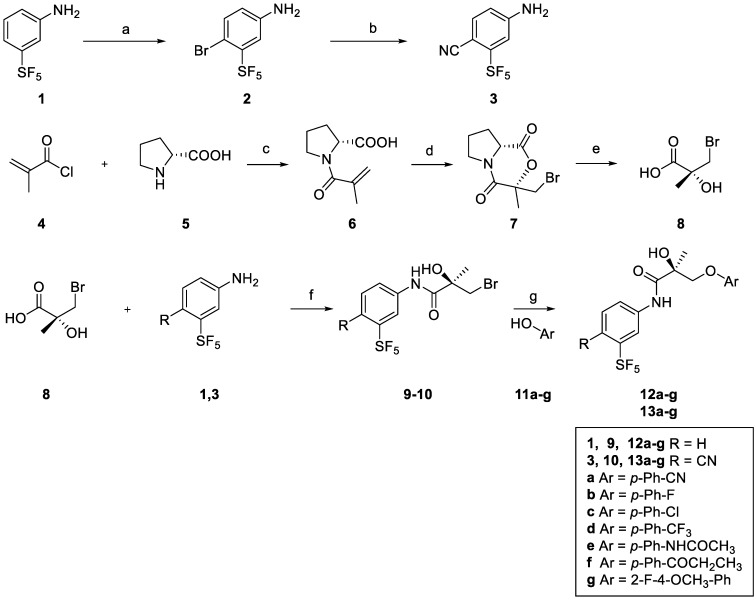
Synthesis of (*S*)-*meta*-SF_5_ derivatives **12a–g**, **13a–g**. Reagents and conditions: (**a**) DBDMH (1,3-Dibromo-5,5-dimethylhydantoin), DMAc, 10 °C 30 min; (**b**) CuCN, NMP (1-Methyl-2-pyrrolidinone), 180 °C, 2 h; (**c**) 2N NaOH, Acetone, 0 °C to rt, 3 h; (**d**) NBS (*N*-Bromosuccinimide), DMF, rt, 16 h; (**e**) 20% HBr(aq), reflux, 16 h; (**f**) **8**, SOCl_2_, DMAc, −10 °C, 3 h followed by **21**, DMAc, rt, 16 h; (**g**) K_2_CO_3_, 2-Propanol, reflux, 4 h.

*Meta*-SF_5_ derivatives **12a–g** and **13a–g** were prepared, as illustrated in Scheme 1. 3-(pentafluorosulfanyl)aniline (**1**) was brominated with 1,3-Dibromo-5,5-dimethylhydantoin in DMAc (N,N-dimethylacetamide) in order to form compound 4-Bromo-3-(pentafluorosulfanyl)aniline (**2**). The second step consisted of the aromatic nucleophilic displacement of bromide from compound **2** using copper cyanide, leading to the formation of 4-amino-2-(pentafluorosulfanyl)benzonitrile (**3**).

(*R*)-*N*-methacryloylproline **6**, prepared using *D*-proline (**5**) and methacryloyl chloride (**4**), was reacted with *N*-bromosuccinimide in DMF (*N*,*N*-dimethylformamide) to afford bromolactone **7** as a single enantiomer. Acidic hydrolysis with aqueous HBr then produced the key intermediate (*R*)-3-Bromo-2-hydroxy-2-methylpropanoic acid (**8**) [21]. Amide coupling with anilines **1,3** using thionyl chloride produced chiral anilides **9–10**. Amide derivatives **9–10** were reacted with different commercial phenols (**11a–g**) using potassium carbonate in 2-propanol to give the desired (*S*)-*meta*-SF_5_ derivatives (**12a–g, 13a–g**).

**Scheme 2:**
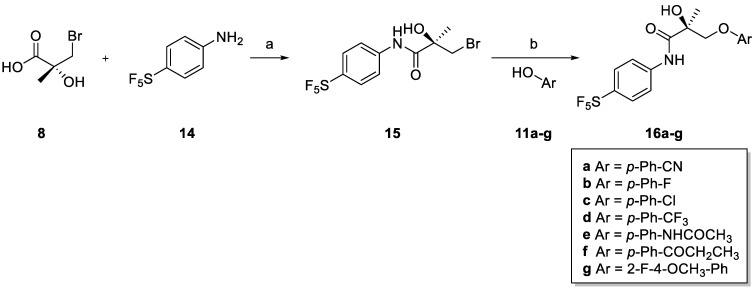
Synthesis of (*S*)-*para*-SF_5_ derivatives **16a–g**. Reagents and conditions: (**a**) **8**, SOCl_2_, DMAc, −10 ^o^C, 3 h followed by **14**, DMAc, rt, 16 h; (**b**) K_2_CO_3_, 2-Propanol, reflux, 4 h.

*Para*-SF_5_ derivatives **16a–g** were prepared, as illustrated in Scheme 2. Amide coupling with 4-(pentafluorosulfanyl)aniline (**14**) using thionyl chloride produced chiral anilide **15**. Amide derivative **15** was reacted with different commercial phenols (**11a–g**) using potassium carbonate in 2-propanol to give the desired (*S*)-*para*-SF_5_ derivatives (**16a–g**).

### 2.2. AR Activity In Vitro

All compounds listed in Table 1 and Table 2 were evaluated in vitro for their agonist activities using a luciferase reporter gene assay with CV-1 (*Cercopithecus aethiops* kidney cell line) and C2C12 cells (mouse myoblast cell line) expressing AR. The results of the reference compound (ostarine) are also shown for comparison in Table 1.

Accordingly, with cyano and SF_5_ moieties, a series of *para*-substituted *O*-linked analogs were synthesized. All of these compounds displayed different degrees of AR agonist activity in CV-1 cells (Table 1). The introduction of the methoxy and *N*-amide groups as an electron-donating group on the *para*-phenyl ring (**13e**,**g**) resulted in significantly decreased AR agonist activity. When electron-withdrawing groups were introduced, compounds **13b**, **13c**, **13d,** and **13f** exhibited weak agonist activity (Table 1). The remaining compounds exhibited less than 66% efficacy of 1 μM dihydrotestosterone (DHT) at the concentrations tested. When the *para*-position of the phenyl ring was cyano moiety as an electron-withdrawing group, compound **13a** showed potent agonist activity: 30 μM elicited 89.5% of the efficacy observed with 1 μM DHT, similar to that of ostarine (85.8% efficacy). The agonistic activity of the compounds in C2C12 cells was comparable.

To investigate the importance of the cyano moiety, compound series **12a–g** and **16a–g** in which the cyano is absent, were synthesized. Unfortunately, **16a–g** in which the SF_5_ group as a bioisostere of the SF_3_ group is in the *para* position were completely inactive, and analogs **12a–g** bearing only SF_5_ group at *meta* position lost AR agonist activity (Table 2). Thus, cyano moiety is required for AR agonism.

## 3. Materials and Methods

All reagents were commercially available and were used without further purification. The solvents used were of analytical grade. Melting points were taken on a Fisher Johns melting point apparatus, uncorrected, and reported in degrees centigrade. ^1^H NMR and ^13^C NMR spectra were scanned on a Bruker DRX-400 (400 MHz) using tetramethylsilane (TMS) as internal standard and one or two of the following solvents: DMSO-*d6* and CDCl_3_. Chemical shifts were given in δ, ppm. Splitting patterns were designated as follows: s: singlet; d: doublet; t: triplet; q: quartet; m: multiplet. The mass spectra (MS) were recorded on a Finnigan MAT-95 mass spectrometer. The purity of all testing compounds was established by HPLC to be >95%. HPLC analysis was performed at room temperature using an Agilent Eclipse XDBC18 (250 mm × 4.6 mm) and as a mobile phase gradient from 5% MeCN/H_2_O (1‰ TFA (trifluoroethanoic acid)) for 1 min, 5% MeCN/H_2_O (1‰ TFA) to 95% MeCN/H_2_O (1‰ TFA) for 9 min, and 95% MeCN/H_2_O (1‰ TFA) for 5 min more, a flow rate of 1 mL/min, and plotted at 254 nm (Supplementary Materials).

### 3.1. General Synthesis

*4-Bromo-3-(pentafluorosulfanyl)aniline* (**2**). In a 100 mL round-bottomed flask, 3-(pentafluorosulfanyl)aniline (**1**) (2 g, 9.13 mmol, 1 eq) in 15 mL DMAc was added to give a yellow solution. The reaction mixture was cooled to −20 °C with stirring on. Then, 1,3-dibromo-5,5-dimethylimidazolidine-2,4-dione (1.435 g, 5.02 mmol, 0.55 eq) was added in 10 mL DMAc. The reaction mixture was held at −20 °C with stirring on for 16 h. The 100 mL water was added. The aq layer was backextracted with EA. The organic layers were combined and washed with water and brine. The organic was dried with Na_2_SO_4_, filtered, and concentrated to give crude product 3.3 g. The crude product was purified by column chromatography to give 4-Bromo-3-(pentafluorosulfanyl)aniline (**2**) (1.7 g, 62.5% yield) as an orange oil. ^1^H NMR (400 MHz, CDCl_3_) δ 7.47 (d, *J* = 8.8 Hz, 1H), 7.12 (s, 1H), 6.97 (d, *J* = 8.8 Hz, 1H), 4.30 (s, 2H). ^19^F NMR (376 MHz, CDCl_3_) δ 84.81–82.68 (m, 1F), 65.22 (d, *J* = 152 Hz, 4F). ^13^C NMR (101 MHz, CDCl_3_) δ 154.32, 154.15, 153.98, 145.52, 136.59, 118.89, 116.06, 116.01, 115.96, 115.90, 104.15. ESI-MS *m*/*z*: 297.97, 300.14 [M + H, M + 2H]^+^.

*4-amino-2-(pentafluorosulfanyl)benzonitrile* (**3**). In a 100 mL round-bottomed flask, 4-Bromo-3-(pentafluorosulfanyl)aniline (**2**) (1.7 g, 5.70 mmol, 1 eq) and cyanocopper (0.613 g, 6.84 mmol, 1.2 eq) were added in 30 mL NMP (1-Methyl-2-pyrrolidinone) to give a brown solution. The reaction vessel was purged with nitrogen. The reaction was heated to 180 °C with stirring on for 4 h. Then, 100 mL 25% EDA (ethylenediamine) (aq) was added. The aq layer was backextracted with EA. The organic layers were combined and washed with 25% EDA (aq) and brine. The organic was dried with Na_2_SO_4_, filtered, and concentrated to give crude product 1.5 g. The crude product was purified by column chromatography to give 4-amino-2-(pentafluorosulfanyl)benzonitrile (**3**) (1.05 g, 75% yield) as orange solid. m.p. 149–151 °C; ^1^H NMR (400 MHz, CDCl_3_) δ 7.54 (d, *J* = 8.4 Hz, 1H), 7.09 (s, 1H), 6.76 (d, *J* = 8.4 Hz, 1H), 4.46 (s, 2H). ^19^F NMR (376 MHz, CDCl_3_) δ 82.40–80.21 (m, 1F), 65.27 (d, *J* = 151.1 Hz, 4F). ^13^C NMR (101 MHz, CDCl_3_) δ 156.35, 150.29, 136.39, 117.38, 116.09, 113.80, 97.05. ESI-MS *m*/*z*: 245.25 [M + H]^+^.

*methacryloyl-D-proline* (**6**). A solution of methacryloyl chloride (**4**) (17.49 mL, 181 mmol, 1.04 eq) in 100 mL acetone was added to a solution of *D*-proline (**5**) (20 g, 174 mmol, 1 eq) in 2M NaOH (100 mL, 200 mmol, 1.15 eq) and 100 mL acetone dropwise at 0 °C for 30 min. The pH of the reaction mixture was maintained within 10~11 range via simultaneous dropwise addition of 5 mL 2M NaOH (aq) during the addition of methacryloyl chloride. The reaction mixture was warmed up to room temperature with stirring on for 16 h. The mixture was concentrated by rotovap. The reaction mixture was washed with Et_2_O. Then, 150 mL 2M HCl was added to adjust pH to 2. The aq layer was backextracted with EA. The organic layers were combined and washed with sat.NaCl (aq). The organic was dried with Na_2_SO_4_, filtered, and concentrated to give methacryloyl-*D*-proline (**6**) (31.5 g, 99% yield) as white solid. The crude product was used until the next step without further purification. m.p. 102–104 °C; ^1^H NMR (400 MHz, CDCl_3_) δ: 9.78 (s, 1H), 5.32 (d, J = 9 Hz, 1H), 5.25–5.03 (m, 1H), 4.57 (s, 1H), 3.63 (s, 2H), 2.22 (dd, J = 14.7, 7.7 Hz, 2H), 2.07–1.84 (m, 5H).

*(3R,8aR)-3-(bromomethyl)-3-methyltetrahydro-1H-pyrrolo[2,1-c][1,4]oxazine-1,4(3H)-dione* (**7**). In a 500 mL round-bottomed flask, methacryloyl-*D*-proline (**6**) (31.5 g, 172 mmol, 1 eq) in 150 mL anhydrous DMF was added to give a colorless solution. The reaction vessel was purged with nitrogen. The reaction mixture was cooled to 0 °C with stirring on for 30 min. 1-bromopyrrolidine-2,5-dione (61.2 g, 344 mmol, 2 eq) in 150 mL anhydrous DMF was added dropwise at 0 °C for 120 min. The reaction mixture was warmed up to room temperature with stirring on for overnight. The mixture was concentrated by rotovap. Then, 1000 mL EA (ethyl acetate) was added. The organic was washed with sat.NaHCO_3_, water, and brine. The organic was dried with Na_2_SO_4_, filtered, and concentrated to give the crude product. Then, 30 mL EA and 150 mL Et_2_O were added. The product was filtered through sintered glass funnel with 30 mL Et_2_O to give (3*R*,8*aR*)-3-(bromomethyl)-3-methyltetrahydro-1*H*-pyrrolo[2,1-c][1,4]oxazine-1,4(3*H*)-dione *(**7**)* (40 g, 89% yield) as white solid. m.p. 158–160 °C; ^1^H NMR (400 MHz, DMSO) δ 4.77–4.66 (m, 1H), 4.03 (d, *J* = 11.5 Hz, 1H), 3.87 (d, *J* = 11.3 Hz, 1H), 3.52 (dd, *J* = 18.7, 9.2 Hz, 1H), 3.40 (t, *J* = 10.3 Hz, 1H), 2.27 (dd, *J* = 17.3, 6.7 Hz, 1H), 1.95 (dt, *J* = 17.5, 8 Hz, 2H), 1.82 (dd, *J* = 19, 10.4 Hz, 1H), 1.58 (s, 3H). ^13^C NMR (101 MHz, DMSO) δ 167.26, 163.07, 83.88, 57.22, 45.41, 37.82, 29.02, 22.92, 21.58.

*(R)-3-Bromo-2-hydroxy-2-methylpropanoic acid* (**8**). In a 250 mL round-bottomed flask, (3*R*,8*aR*)-3-(bromomethyl)-3-methyltetrahydro-1*H*-pyrrolo[2,1-c][1,4]oxazine-1,4(3*H*)-dione *(**7**)* (25 g, 95 mmol, 1 eq) in HBr (250 mL, 921 mmol, 9.7 eq) was added to give an orange solution. The reaction was heated to 100°C with stirring on for 16 h. Then, 250 mL sat.NaCl was added. The aq layer was backextracted with EA. The organic layers were combined and washed with sat.NaCl (aq). The organic was washed with sat.NaHCO_3_. Later, 45 mL 12M HCl was added to adjust pH to 1. The aq layer was backextracted with EA. The organic layers were combined and washed with water and sat.NaCl (aq). The organic was dried with Na_2_SO_4_, filtered, and concentrated to give (*R*)-3-Bromo-2-hydroxy-2-methylpropanoic acid (**8**) (12.2 g, 69.9% yield) as white solid. m.p. 111–113 °C; ^1^H NMR (400 MHz, DMSO) δ 12.84 (s, 1H), 5.43 (s, 1H), 3.65 (d, *J* = 10.2 Hz, 1H), 3.54 (d, *J* = 10 Hz, 1H), 1.37 (s, 3H). ^13^C NMR (101 MHz, DMSO) δ 174.51, 73.11, 40.94, 24.45. [α]_D_^20^ = +9.9 (*c* = 1.1, MeOH).

### 3.2. General Procedure for the Synthesis of 9, 10, and 15

In a 100 mL round-bottomed flask, (*R*)-3-Bromo-2-hydroxy-2-methylpropanoic acid (1.7 eq) in anhydrous 25 mL DMAc was added to give a yellow solution. The reaction vessel was purged with nitrogen. The reaction mixture was cooled to −10 °C with stirring on. Thionyl chloride (1.85 eq) was added slowly. The reaction mixture was held at −5 °C with stirring on for 3 h. ***1, 3,*** *or **14*** (1 eq) in 15 mL anhydous DMAc was added. The reaction mixture was warmed up to room temperaure with stirring on for 16 h. Then, 60 mL water was added. The aq layer was backextracted with EA. The organic layers were combined and washed with sat.NaHCO_3_ (aq), sat.NH_4_Cl (aq), and sat.NaCl (aq). The organic was dried with Na_2_SO_4_, filtered, and concentrated to give crude product. The crude product was purified by column chromatography to give ***9, 10,*** *and **15***.

*(R)-3-Bromo-2-hydroxy-2-methyl-N-(3-(pentafluorosulfanyl)phenyl)propanamide* (**9**). Yellow solid (1.7 g, 96% yield). m.p. 105–107 °C; ^1^H NMR (400 MHz, CDCl_3_) δ 8.77 (s, 1H), 8.03 (s, 1H), 7.74 (d, *J* = 7.7 Hz, 1H), 7.51 (d, *J* = 8 Hz, 1H), 7.42 (t, *J* = 7.9 Hz, 1H), 4.01 (d, *J* = 10.4 Hz, 1H), 3.59 (d, *J* = 10.4 Hz, 1H), 3.01 (s, 1H), 1.61 (s, 3H). ^19^F NMR (376 MHz, CDCl_3_) δ 84.94–82.68 (m, 1F), 62.66 (d, *J* = 150.2 Hz, 4F). ^13^C NMR (101 MHz, CDCl_3_) δ 171.12, 154.28, 137.42, 129.28, 122.63, 122.04, 117.46, 75.39, 41.50, 24.79. ESI-MS *m*/*z*: 409.14, 411.22 [M + H, M + 2H]^+^. [α]_D_^20^ = −28.1 (*c* = 1.1, MeOH).

*(R)-3-Bromo-N-(4-cyano-3-(pentafluorosulfanyl)phenyl)-2-hydroxy-2-methylpropanamide* (**10**). Light-yellow solid (450 mg, 49% yield). m.p. 125–127 °C; ^1^H NMR (400 MHz, CDCl_3_) δ 9.03 (s, 1H), 8.22 (s, 1H), 7.97 (d, *J* = 8.3 Hz, 1H), 7.81 (d, *J* = 7.9 Hz, 1H), 4.02 (d, *J* = 10.5 Hz, 1H), 3.60 (d, *J* = 10.5 Hz, 1H), 3.01 (s, 1H), 1.65 (s, 3H). ^19^F NMR (376 MHz, CDCl_3_) δ 81.81–78.79 (m, 1F), 65.52 (d, *J* = 151.5 Hz, 4F). ^13^C NMR (101 MHz, CDCl_3_) δ 171.63, 155.37, 141.15, 136.09, 121.67, 119.13, 116.10, 104.70, 75.59, 40.98, 24.83. ESI-MS *m*/*z*: 384.24, 386.19 [M + H, M + 2H]^+^. [α]_D_^20^ = –23.3 (*c* = 0.1, MeOH).

*(R)-3-Bromo-2-hydroxy-2-methyl-N-(4-(pentafluorosulfanyl)phenyl)propanamide* (**15**). Light-yellow solid (1.1 g, 88% yield). m.p. 117–119 °C; ^1^H NMR (400 MHz, CDCl_3_) δ 8.85 (s, 1H), 7.73 (d, *J* = 9.4 Hz, 2H), 7.69 (d, *J* = 9.3 Hz, 2H), 4.02 (d, *J* = 10.4 Hz, 1H), 3.60 (d, *J* = 10.5 Hz, 1H), 3.21 (s, 1H), 1.63 (s, 3H). ^19^F NMR (376 MHz, CDCl_3_) δ 85.87–83.96 (m, 1F), 63.38 (d, *J* = 150.3 Hz, 4F). ^13^C NMR (101 MHz, CDCl_3_) δ 171.30, 149.60, 139.66, 127.13, 127.09, 127.04, 119.10, 75.42, 41.38, 24.80. ESI-MS *m*/*z*: 384.24, 386.26 [M + H, M + 2H]^+^. [α]_D_^20^ = −28 (*c* = 1, MeOH).

### 3.3. General Procedure for the Synthesis of 12a–g, 13a–g, and 16a–g

In a 50 mL round-bottomed flask, ***9***, ***10,*** or ***15*** (1 eq), different commercial phenols ***11a–g*** (1.5 eq), and potassium carbonate (3 eq) were added in 20 mL 2-propanol to give a white suspension. The reaction vessel was purged with nitrogen. The reaction was heated to 85 °C with stirring on for 16 h. The reaction mixture was filtered through sintered glass funnel. The mixture was concentrated by rotovap. The crude product was purified by column chromatography to give ***12a–g***, ***13a–g,*** *and **16a–g***.

*(S)-3-(4-cyanophenoxy)-2-hydroxy-2-methyl-N-(3-(pentafluoro-l6-sulfanyl)phenyl)propanamide* (**12a**). White solid (50 mg, 46% yield). m.p. 70–72 °C; ^1^H NMR (400 MHz, CDCl_3_) δ 8.92 (s, 1H), 8.09 (t, *J* = 2 Hz, 1H), 7.76 (d, *J* = 8 Hz, 1H), 7.57 (d, *J* = 8.8 Hz, 2H), 7.53 (dd, *J* = 8.4, 1.3 Hz, 1H), 7.44 (t, *J* = 8.2 Hz, 1H), 6.98 (d, *J* = 8.9 Hz, 2H), 4.50 (d, *J* = 9.2 Hz, 1H), 4.06 (d, *J* = 9.2 Hz, 1H), 3.43 (s, 1H), 1.62 (s, 3H). ^19^F NMR (376 MHz, CDCl_3_) δ 84.99–82.98 (m, 1F), 62.66 (d, *J* = 150.2 Hz, 4F). ^13^C NMR (101 MHz, CDCl_3_) δ 171.80, 161.13, 154.29, 137.55, 134.12, 129.30, 122.52, 122, 118.80, 117.40, 116.33, 115.48, 105.10, 75.58, 72.74, 23.03. HRMS(ESI)*m/z* calcd for C_17_H_15_F_5_N_2_O_3_S [M + H]^+^: 423.0802, Found: 423.0818. [α]_D_^20^ = +3 (*c* = 0.1, MeOH).

*(S)-3-(4-fluorophenoxy)-2-hydroxy-2-methyl-N-(3-(pentafluoro-l6-sulfanyl)phenyl)propanamide* (**12b**). White solid (95 mg, 88% yield). m.p. 91–93 °C; ^1^H NMR (400 MHz, CDCl_3_) δ 8.94 (s, 1H), 8.08 (t, *J* = 1.9 Hz, 1H), 7.74 (d, *J* = 8.2 Hz, 1H), 7.50 (dd, *J* = 8.3, 1.2 Hz, 1H), 7.40 (t, *J* = 8.2 Hz, 1H), 6.94 (dt, *J* = 11.9, 3 Hz, 2H), 6.87–6.78 (m, 2H), 4.40 (d, *J* = 9 Hz, 1H), 3.94 (d, *J* = 9 Hz, 1H), 3.58 (s, 1H), 1.57 (s, 3H). ^19^F NMR (376 MHz, CDCl_3_) δ 85.25–82.86 (m, 1F), 62.63 (d, *J* = 150.1 Hz, 4F), –122.19 (s, 1F). ^13^C NMR (101 MHz, CDCl_3_) δ 172.39, 159.06, 156.67, 153.98, 137.67, 129.23, 122.54, 121.85, 117.38, 116.14, 116.06, 115.98, 115.90, 75.62, 73.32, 22.94. HRMS(ESI)*m/z* calcd for C_16_H_15_F_6_NO_3_S [M + H]^+^: 416.0755, Found: 416.0744. [α]_D_^20^ = –3.3 (*c* = 0.3, MeOH).

*(S)-3-(4-chlorophenoxy)-2-hydroxy-2-methyl-N-(3-(pentafluoro-l6-sulfanyl)phenyl)propanamide* (**12c**). White solid (110 mg, 98% yield). m.p. 82–84 °C; ^1^H NMR (400 MHz, CDCl_3_) δ 8.93 (s, 1H), 8.08 (t, *J* = 2 Hz, 1H), 7.72 (d, *J* = 8 Hz, 1H), 7.50 (dd, *J* = 8.3, 1.4 Hz, 1H), 7.40 (t, *J* = 8.2 Hz, 1H), 7.23 – 7.17 (m, 2H), 6.86–6.78 (m, 2H), 4.39 (d, *J* = 9 Hz, 1H), 3.94 (d, *J* = 9.2 Hz, 1H), 3.58 (s, 1H), 1.57 (s, 3H). ^19^F NMR (376 MHz, CDCl_3_) δ 84.97–82.86 (m, 1F), 62.65 (d, *J* = 150.2 Hz, 4F). ^13^C NMR (101 MHz, CDCl_3_) δ 172.30, 156.50, 154.25, 137.63, 129.50, 129.24, 126.84, 122.56, 121.90, 117.39, 116.11, 75.59, 72.91, 22.95. HRMS(ESI)*m/z* calcd for C_16_H_15_ClF_5_NO_3_S [M + H]^+^: 432.0460, Found: 432.0455. [α]_D_^20^ = +1.2 (*c* = 0.2, MeOH).

*(S)-2-hydroxy-2-methyl-N-(3-(pentafluoro-l6-sulfanyl)phenyl)-3-(4-(trifluoromethyl)phenoxy)propanamide* (**12d**). White solid (70 mg, 58% yield). m.p. 96–98 °C; ^1^H NMR (400 MHz, CDCl_3_) δ 8.93 (s, 1H), 8.09 (s, 1H), 7.74 (d, *J* = 8 Hz, 1H), 7.53 (t, *J* = 6.8 Hz, 3H), 7.42 (t, *J* = 8.2 Hz, 1H), 6.97 (d, *J* = 8.4 Hz, 2H), 4.49 (d, *J* = 9 Hz, 1H), 4.03 (d, *J* = 9 Hz, 1H), 3.44 (s, 1H), 1.60 (s, 3H). ^19^F NMR (376 MHz, CDCl_3_) δ 84.86 – 82.96 (m, 1F), 62.64 (d, *J* = 150.1 Hz, 4F), –61.69 (s, 3F). ^13^C NMR (101 MHz, CDCl_3_) δ 172.03, 160.21, 154.26, 137.55, 129.28, 127.10, 127.06, 125.54, 124.23, 123.90, 122.85, 122.51, 121.96, 117.37, 114.72, 75.59, 72.57, 23. HRMS(ESI)*m/z* calcd for C_17_H_15_F_8_NO_3_S [M + Na]^+^: 488.0543, Found: 488.0521. [α]_D_^20^ = –2.5 (*c* = 0.2, MeOH).

*(S)-3-(4-acetamidophenoxy)-2-hydroxy-2-methyl-N-(3-(pentafluoro-l6-sulfanyl)phenyl)propanamide* (**12e**). White solid (100 mg, 85% yield). m.p. 79–81 °C; ^1^H NMR (400 MHz, DMSO) δ 10.19 (s, 1H), 9.76 (s, 1H), 8.53 (s, 1H), 8.06 (d, *J* = 7.5 Hz, 1H), 7.57 (p, *J* = 8.3 Hz, 2H), 7.45 (d, *J* = 8.9 Hz, 2H), 6.86 (d, *J* = 8.9 Hz, 2H), 6.13 (s, 1H), 4.17 (d, *J* = 9.5 Hz, 1H), 3.93 (d, *J* = 9.5 Hz, 1H), 2 (s, 3H), 1.43 (s, 3H). ^19^F NMR (376 MHz, DMSO) δ 88.61–86.49 (m, 1F), 63.66 (d, *J* = 150.6 Hz, 4F). ^13^C NMR (101 MHz, DMSO) δ 173.81, 167.68, 154.23, 152.87, 139.30, 132.74, 129.49, 123.32, 120.33, 116.73, 114.55, 74.67, 73.73, 23.76, 23.04. HRMS(ESI)*m/z* calcd for C_18_H_19_F_5_N_2_O_4_S [M + H]^+^: 455.1064, Found: 455.1046. [α]_D_^20^ = –2.5 (*c* = 1.2, MeOH).

*(S)-2-hydroxy-2-methyl-N-(3-(pentafluoro-l6-sulfanyl)phenyl)-3-(4-propionylphenoxy)propanamide* (**12f**). White solid (95 mg, 80% yield). m.p. 76–78 °C; ^1^H NMR (400 MHz, CDCl_3_) δ 9.11 (s, 1H), 8.12 (s, 1H), 7.85 (d, *J* = 8.7 Hz, 2H), 7.74 (d, *J* = 8 Hz, 1H), 7.49 (d, *J* = 8.3 Hz, 1H), 7.39 (t, *J* = 8.2 Hz, 1H), 6.86 (d, *J* = 8.5 Hz, 2H), 4.47 (d, *J* = 9.3 Hz, 1H), 4.26 (s, 1H), 4.05 (d, *J* = 9.2 Hz, 1H), 2.91 (q, *J* = 7.2 Hz, 2H), 1.60 (s, 3H), 1.17 (t, *J* = 7.3 Hz, 3H). ^19^F NMR (376 MHz, CDCl_3_) δ 84.90–82.96 (m, 1F), 62.65 (d, *J* = 150.2 Hz, 4F). ^13^C NMR (101 MHz, CDCl_3_) δ 200.16, 172.43, 161.78, 154.17, 137.71, 130.52, 130.24, 129.21, 122.60, 121.82, 117.38, 114.38, 75.55, 72.78, 31.48, 22.94, 8.36. HRMS(ESI)*m/z* calcd for C_19_H_20_F_5_NO_4_S [M + Na]^+^: 476.0931, Found: 476.0923. [α]_D_^20^ = +1 (*c* = 0.2, MeOH).

*(S)-3-(2-fluoro-4-methoxyphenoxy)-2-hydroxy-2-methyl-N-(3-(pentafluoro-l6-sulfanyl)phenyl)propanamide* (**12g**). White solid (100 mg, 86% yield). m.p. 92–94 °C; ^1^H NMR (400 MHz, CDCl_3_) δ 8.98 (s, 1H), 8.09 (t, *J* = 2 Hz, 1H), 7.71 (d, *J* = 8.2 Hz, 1H), 7.49 (dd, *J* = 8.3, 1.4 Hz, 1H), 7.39 (t, *J* = 8.2 Hz, 1H), 6.93 (t, *J* = 9.2 Hz, 1H), 6.64 (dd, *J* = 12.6, 2.9 Hz, 1H), 6.56 (ddd, *J* = 9, 2.9, 1.5 Hz, 1H), 4.43 (d, *J* = 9.3 Hz, 1H), 3.97 (d, *J* = 9.2 Hz, 1H), 3.90 (s, 1H), 3.72 (s, 3H), 1.56 (s, 3H). ^19^F NMR (376 MHz, CDCl_3_) δ 85.16 – 82.99 (m, 1F), 62.64 (d, *J* = 150.1 Hz, 4F), –131.31 (s, 1F). ^13^C NMR (101 MHz, CDCl_3_) δ 172.62, 155.42, 155.33, 154.62, 154.20, 152.18, 139.77, 139.65, 137.74, 129.15, 122.63, 121.77, 118, 117.98, 117.46, 109.15, 109.12, 103.35, 103.13, 75.61, 75.38, 55.74, 22.94. HRMS(ESI)*m/z* calcd for C_17_H_17_F_6_NO_4_S [M + Na]^+^: 468.0680, Found: 468.0692. [α]_D_^20^ = −7 (*c* = 0.1, MeOH).

*(S)-N-(4-cyano-3-(pentafluoro-l6-sulfanyl)phenyl)-3-(4-cyanophenoxy)-2-hydroxy-2-methylpropanamide* (**13a**). White solid (260 mg, 60% yield). m.p. 77–79 °C; ^1^H NMR (400 MHz, CDCl_3_) δ 9.20 (s, 1H), 8.27 (s, 1H), 7.96 (d, *J* = 8.4 Hz, 1H), 7.78 (d, *J* = 8.5 Hz, 1H), 7.57 (d, *J* = 8 Hz, 2H), 6.98 (d, *J* = 8.2 Hz, 2H), 4.50 (d, *J* = 9.2 Hz, 1H), 4.07 (d, *J* = 9 Hz, 1H), 3.53 (s, 1H), 1.63 (s, 3H). ^19^F NMR (376 MHz, CDCl_3_) δ 81.19–79.05 (m, 1F), 65.53 (d, *J* = 151.3 Hz, 4F). ^13^C NMR (101 MHz, CDCl_3_) δ 172.36, 161.02, 155.55, 141.42, 136.05, 134.13, 121.61, 119.10, 118.76, 116.13, 115.46, 105.18, 104.46, 75.79, 72.60, 22.99. HRMS(ESI)*m/z* calcd for C_18_H_14_F_5_N_3_O_3_S [M + Na]^+^: 470.0574, Found: 470.0566. [α]_D_^20^ = −5.8 (*c* = 1, MeOH).

*(S)-N-(4-cyano-3-(pentafluoro-l6-sulfanyl)phenyl)-3-(4-fluorophenoxy)-2-hydroxy-2-methylpropanamide* (**13b**). White solid (40 mg, 62% yield). m.p. 85–87 °C; ^1^H NMR (400 MHz, CDCl_3_) δ 9.16 (s, 1H), 8.25 (s, 1H), 7.96 (d, *J* = 8.5 Hz, 1H), 7.79 (d, *J* = 8.4 Hz, 1H), 6.98 (t, *J* = 8.4 Hz, 2H), 6.86 (dd, *J* = 8.8, 4.1 Hz, 2H), 4.43 (d, *J* = 9 Hz, 1H), 3.95 (d, *J* = 9 Hz, 1H), 3.46 (s, 1H), 1.59 (s, 3H). ^19^F NMR (376 MHz, CDCl_3_) δ 81.37–79.25 (m, 1F), 65.53 (d, *J* = 151.2 Hz, 4F), –121.89 (s, 1F). ^13^C NMR (101 MHz, CDCl_3_) δ 172.76, 159.14, 156.76, 155.58, 153.75, 141.41, 136.07, 121.55, 119.04, 116.25, 116.15, 116.04, 116.02, 115.96, 104.48, 75.89, 73.05, 22.92. HRMS(ESI)*m/z* calcd for C_17_H_14_F_6_N_2_O_3_S [M + H]^+^: 441.0708, Found: 441.0723. [α]_D_^20^ = −0.7 (*c* = 0.2, MeOH).

*(S)-3-(4-chlorophenoxy)-N-(4-cyano-3-(pentafluoro-l6-sulfanyl)phenyl)-2-hydroxy-2-methylpropanamide* (**13c**). White solid (51 mg, 76% yield). m.p. 65–67 °C; ^1^H NMR (400 MHz, CDCl_3_) δ 9.14 (s, 1H), 8.24 (s, 1H), 7.95 (d, *J* = 8.5 Hz, 1H), 7.79 (d, *J* = 8.5 Hz, 1H), 7.25 (d, *J* = 9 Hz, 2H), 6.84 (d, *J* = 8.8 Hz, 2H), 4.44 (d, *J* = 9 Hz, 1H), 3.97 (d, *J* = 9 Hz, 1H), 3.40 (s, 1H), 1.59 (s, 3H). ^19^F NMR (376 MHz, CDCl_3_) δ 81.55 – 78.71 (m, 1F), 65.49 (d, *J* = 151.3 Hz, 4F). ^13^C NMR (101 MHz, CDCl_3_) δ 172.61, 156.23, 155.58, 141.36, 136.07, 129.60, 127.10, 121.55, 119.04, 116.14, 116.07, 104.53, 75.85, 72.60, 22.96. HRMS(ESI)*m/z* calcd for C_17_H_14_ClF_5_N_2_O_3_S [M + H]^+^: 457.0412, Found: 457.0408. [α]_D_^20^ = −3 (*c* = 0.1, MeOH).

*(S)-N-(4-cyano-3-(pentafluoro-l6-sulfanyl)phenyl)-2-hydroxy-2-methyl-3-(4 (trifluoromethyl)phenoxy)propanamide* (**13d**). White solid (30 mg, 42% yield). m.p. 69–71 °C; ^1^H NMR (400 MHz, CDCl_3_) δ 9.19 (s, 1H), 8.27 (s, 1H), 7.95 (d, *J* = 8.3 Hz, 1H), 7.77 (d, *J* = 8.5 Hz, 1H), 7.55 (d, *J* = 8.4 Hz, 2H), 6.98 (d, *J* = 8.5 Hz, 2H), 4.51 (d, *J* = 9.2 Hz, 1H), 4.05 (d, *J* = 9 Hz, 1H), 3.49 (s, 1H), 1.62 (s, 3H). ^19^F NMR (376 MHz, CDCl_3_) δ 80.94 – 79.24 (m, 1F), 65.52 (d, *J* = 151.2 Hz, 4F), –61.72 (s, 3F). ^13^C NMR (101 MHz, CDCl_3_) δ 172.54, 160.05, 155.73, 155.55, 155.36, 141.42, 136.05, 131.62, 127.13, 127.10, 127.06, 125.50, 124.69, 124.36, 124.03, 123.71, 122.80, 121.58, 119.07, 116.17, 114.72, 104.41, 75.84, 72.43, 22.96. HRMS(ESI)*m/z* calcd for C_18_H_14_F_8_N_2_O_3_S [M + Na]^+^: 513.0495, Found: 513.0483. [α]_D_^20^ = −3 (*c* = 0.1, MeOH).

*(S)-3-(4-acetamidophenoxy)-N-(4-cyano-3-(pentafluoro-l6-sulfanyl)phenyl)-2-hydroxy-2-methylpropanamide* (**13e**). White solid (40 mg, 57% yield). m.p. 112–114 °C; ^1^H NMR (400 MHz, DMSO) δ 10.69 (s, 1H), 9.78 (s, 1H), 8.80 (s, 1H), 8.35 (d, *J* = 8.7 Hz, 1H), 8.12 (d, *J* = 8.7 Hz, 1H), 7.45 (d, *J* = 9 Hz, 2H), 6.85 (d, *J* = 9 Hz, 2H), 6.29 (s, 1H), 4.18 (d, *J* = 9.7 Hz, 1H), 3.95 (d, *J* = 9.6 Hz, 1H), 1.99 (s, 3H), 1.44 (s, 3H). ^19^F NMR (376 MHz, DMSO) δ 83.68 (m, 1F), 66.17 (d, *J* = 152 Hz, 4F). ^13^C NMR (101 MHz, DMSO) δ 174.78, 167.68, 154.14, 143.36, 136.56, 132.80, 122.40, 120.33, 118.99, 116.37, 114.59, 101.46, 74.86, 73.74, 23.76, 22.97. HRMS(ESI)*m/z* calcd for C_19_H_18_F_5_N_3_O_4_S [M + Na]^+^: 502.0836, Found: 502.0829. [α]_D_^20^ = −5.2 (*c* = 0.3, MeOH).

*(S)-N-(4-cyano-3-(pentafluoro-l6-sulfanyl)phenyl)-2-hydroxy-2-methyl-3-(4-propionylphenoxy)propanamide* (**13f**). White solid (37 mg, 53% yield). m.p. 66–68 °C; ^1^H NMR (400 MHz, DMSO) δ 10.71 (s, 1H), 8.79 (d, *J* = 1.9 Hz, 1H), 8.35 (d, *J* = 8.7 Hz, 1H), 8.12 (d, *J* = 8.7 Hz, 1H), 7.91 (d, *J* = 8.9 Hz, 2H), 7.03 (d, *J* = 8.8 Hz, 2H), 6.36 (s, 1H), 4.33 (d, *J* = 9.9 Hz, 1H), 4.09 (d, *J* = 9.8 Hz, 1H), 2.96 (q, *J* = 7.2 Hz, 2H), 1.46 (s, 3H), 1.06 (t, *J* = 7.2 Hz, 3H). ^19^F NMR (376 MHz, DMSO) δ 83.81 (p, *J* = 153.6 Hz, 1F), 66.16 (d, *J* = 151.9 Hz, 4F). ^13^C NMR (101 MHz, DMSO) δ 198.75, 174.59, 162.06, 143.31, 136.56, 129.99, 129.71, 122.45, 119.04, 116.34, 114.41, 101.52, 74.75, 73.59, 30.75, 22.92, 8.23. HRMS(ESI)*m/z* calcd for C_20_H_19_F_5_N_2_O_4_S [M + Na]^+^: 501.0883, Found: 501.0883. [α]_D_^20^ = −1 (*c* = 0.1, MeOH).

*(S)-N-(4-cyano-3-(pentafluoro-l6-sulfanyl)phenyl)-3-(2-fluoro-4-methoxyphenoxy)-2-hydroxy-2-methylpropanamide* (**13g**). White solid (55 mg, 80% yield). m.p. 58–60 °C; ^1^H NMR (400 MHz, CDCl_3_) δ 9.24 (s, 1H), 8.26 (s, 1H), 7.93 (d, *J* = 8.5 Hz, 1H), 7.76 (d, *J* = 8.4 Hz, 1H), 6.93 (t, *J* = 9.1 Hz, 1H), 6.64 (d, *J* = 12.7 Hz, 1H), 6.59 (d, *J* = 8.9 Hz, 1H), 4.45 (d, *J* = 9.2 Hz, 1H), 3.97 (d, *J* = 9.2 Hz, 1H), 3.84 (s, 1H), 3.74 (s, 3H), 1.57 (s, 3H). ^19^F NMR (376 MHz, CDCl_3_) δ 81.73–79.26 (m, 1F), 65.50 (d, *J* = 151.3 Hz, 4F), –131.34 (s, 1F). ^13^C NMR (101 MHz, CDCl_3_) δ 173.15, 155.56, 155.47, 155.28, 154.60, 152.16, 141.55, 139.54, 139.43, 135.97, 121.66, 119.13, 118.08, 118.06, 116.22, 109.20, 109.16, 104.26, 103.35, 103.14, 75.68, 75.54, 55.79, 22.85. HRMS(ESI)*m/z* calcd for C_18_H_16_F_6_N_2_O_4_S [M + Na]^+^: 493.0633, Found: 493.0601. [α]_D_^20^ = −2.5 (*c* = 0.1, MeOH).

*(S)-3-(4-cyanophenoxy)-2-hydroxy-2-methyl-N-(4-(pentafluoro-l6-sulfanyl)phenyl)propanamide* (**16a**). White solid (140 mg, 71% yield). m.p. 73–75 °C; ^1^H NMR (400 MHz, CDCl_3_) δ 8.87 (s, 1H), 7.68 – 7.59 (m, 4H), 7.49 (d, *J* = 8.5 Hz, 2H), 6.90 (d, *J* = 8.5 Hz, 2H), 4.42 (d, *J* = 9.2 Hz, 1H), 3.99 (d, *J* = 9.2 Hz, 1H), 3.34 (s, 1H), 1.54 (s, 3H). ^19^F NMR (376 MHz, CDCl_3_) δ 84.93 (p, *J* = 150.4 Hz, 1F), 63.41 (d, *J* = 150.1 Hz, 4F). ^13^C NMR (101 MHz, CDCl_3_) δ 170.80, 160.11, 138.79, 133.11, 126.10, 117.97, 117.76, 114.47, 104.13, 74.61, 71.75, 22.02. HRMS(ESI)*m/z* calcd for C_17_H_15_F_5_N_2_O_3_S [M + H]^+^: 423.0802, Found: 423.0818. [α]_D_^20^ = +0.2 (*c* = 0.9, MeOH).

*(S)-3-(4-fluorophenoxy)-2-hydroxy-2-methyl-N-(4-(pentafluoro-l6-sulfanyl)phenyl)propanamide* (**16b**). White solid (90 mg, 83% yield). m.p. 87–89 °C; ^1^H NMR (400 MHz, CDCl_3_) δ 8.93 (s, 1H), 7.73 (d, *J* = 9.5 Hz, 2H), 7.70 (d, *J* = 9.8 Hz, 2H), 6.98 (t, *J* = 8.5 Hz, 2H), 6.86 (dd, *J* = 9.1, 4.1 Hz, 2H), 4.42 (d, *J* = 9 Hz, 1H), 3.95 (d, *J* = 8.9 Hz, 1H), 3.40 (s, 1H), 1.58 (s, 3H). ^19^F NMR (376 MHz, CDCl_3_) δ 86.09–84.13 (m, 1F), 63.41 (d, *J* = 150.1 Hz, 4F), –122.12 (s, 1F). ^13^C NMR (101 MHz, CDCl_3_) δ 172.27, 159.09, 156.71, 153.90, 153.88, 139.90, 127.14, 127.10, 127.05, 118.92, 116.20, 116.05, 115.97, 75.65, 73.21, 23. HRMS(ESI)*m/z* calcd for C_16_H_15_F_6_NO_3_S [M + H]^+^: 416.0755, Found: 416.0744. [α]_D_^20^ = −6.2 (*c* = 0.2, MeOH).

*(S)-3-(4-chlorophenoxy)-2-hydroxy-2-methyl-N-(4-(pentafluoro-l6-sulfanyl)phenyl)propanamide* (**16c**). White solid (88 mg, 78% yield). m.p. 65–68 °C; ^1^H NMR (400 MHz, CDCl_3_) δ 8.94 (s, 1H), 7.70 (q, *J* = 9.5 Hz, 4H), 7.22 (d, *J* = 8.5 Hz, 2H), 6.83 (d, *J* = 8.5 Hz, 2H), 4.41 (d, *J* = 8.9 Hz, 1H), 3.95 (d, *J* = 9 Hz, 1H), 3.48 (s, 1H), 1.58 (s, 3H). ^19^F NMR (376 MHz, CDCl_3_) δ 84.98 (d, *J* = 150.4 Hz,, 1F), 63.42 (d, *J* = 150.3 Hz, 4F). ^13^C NMR (101 MHz, CDCl_3_) δ 172.25, 156.41, 149.45, 139.85, 129.54, 127.08, 126.91, 118.95, 116.08, 75.63, 72.81, 23. HRMS(ESI)*m/z* calcd for C_16_H_15_ClF_5_NO_3_S [M + H]^+^: 432.0460, Found: 432.0455. [α]_D_^20^ = −1.7 (*c* = 0.5, MeOH).

*(S)-2-hydroxy-2-methyl-N-(4-(pentafluorosulfanyl)phenyl)-3-(4-(trifluoromethyl)phenoxy)propanamide* (**16d**). White solid (20 mg, 17% yield). m.p. 78–80 °C; ^1^H NMR (400 MHz, CDCl_3_) δ 8.93 (s, 1H), 7.73 (d, *J* = 9.5 Hz, 2H), 7.69 (d, *J* = 9.5 Hz, 2H), 7.55 (d, *J* = 8.8 Hz, 2H), 6.98 (d, *J* = 8.7 Hz, 2H), 4.49 (d, *J* = 9 Hz, 1H), 4.04 (d, *J* = 9 Hz, 1H), 3.37 (s, 1H), 1.61 (s, 3H). ^19^F NMR (376 MHz, CDCl_3_) δ 84.90 (p, *J* = 150.4 Hz, 1F), 63.39 (d, *J* = 150.2 Hz, 4F), –61.70 (s, 3F). ^13^C NMR (101 MHz, CDCl_3_) δ 171.98, 160.17, 149.53, 139.81, 127.15, 127.12, 127.08, 127.06, 125.52, 124.33, 124, 122.83, 118.96, 114.75, 75.63, 72.59, 23.03. HRMS(ESI)*m/z* calcd for C_17_H_15_F_8_NO_3_S [M + Na]^+^: 488.0543, Found: 488.0521. [α]_D_^20^ = −3.7 (*c* = 0.3, MeOH).

*(S)-3-(4-acetamidophenoxy)-2-hydroxy-2-methyl-N-(4-(pentafluorosulfanyl)phenyl)propanamide* (**16e**). White solid (98 mg, 83% yield). m.p. 156–157 °C; ^1^H NMR (400 MHz, DMSO) δ 10.17 (s, 1H), 9.77 (s, 1H), 8.02 (d, *J* = 8.9 Hz, 2H), 7.85 (d, *J* = 9.2 Hz, 2H), 7.45 (d, *J* = 8.9 Hz, 2H), 6.85 (d, *J* = 8.9 Hz, 2H), 6.17 (s, 1H), 4.17 (d, *J* = 9.5 Hz, 1H), 3.94 (d, *J* = 9.5 Hz, 1H), 2 (s, 3H), 1.43 (s, 3H). ^19^F NMR (376 MHz, DMSO) δ 89.74–87.61 (m, 1F), 64.86 (d, *J* = 150.7 Hz, 5F). ^13^C NMR (101 MHz, DMSO) δ 173.79, 167.68, 154.24, 141.77, 132.74, 126.47, 120.36, 119.57, 114.57, 74.73, 73.78, 23.75, 22.99. HRMS(ESI)*m/z* calcd for C_18_H_19_F_5_N_2_O_4_S [M + Na]^+^: 477.0883, Found: 477.0859. [α]_D_^20^ = −0.6 (*c* = 0.5, MeOH).

*(S)-2-hydroxy-2-methyl-N-(4-(pentafluorosulfanyl)phenyl)-3-(4-propionylphenoxy)propanamide* (**16f**). White solid (100 mg, 85% yield). m.p. 100–102 °C; ^1^H NMR (400 MHz, CDCl_3_) δ 8.99 (s, 1H), 7.91 (d, *J* = 8.8 Hz, 2H), 7.71 (s, 4H), 6.91 (d, *J* = 8.8 Hz, 2H), 4.49 (d, *J* = 9.2 Hz, 1H), 4.06 (d, *J* = 9.2 Hz, 1H), 3.66 (s, 1H), 2.94 (q, *J* = 7.2 Hz, 2H), 1.61 (s, 3H), 1.20 (t, *J* = 7.2 Hz, 3H). ^19^F NMR (376 MHz, CDCl_3_) δ 86.14–83.94 (m, 1F), 63.41 (d, *J* = 150.2 Hz, 4F). ^13^C NMR (101 MHz, CDCl_3_) δ 199.69, 172.11, 161.50, 149.46, 139.89, 130.88, 130.28, 127.07, 118.96, 114.40, 75.60, 72.59, 31.51, 23.04, 8.35. HRMS(ESI)*m/z* calcd for C_19_H_20_F_5_NO_4_S [M + Na]^+^: 476.0931, Found: 476.0923. [α]_D_^20^ = +0.2 (*c* = 0.7, MeOH).

*(S)-3-(2-fluoro-4-methoxyphenoxy)-2-hydroxy-2-methyl-N-(4-(pentafluorosulfanyl)phenyl)propanamide* (**16g**). White solid (100 mg, 86% yield). m.p. 91–93 °C; ^1^H NMR (400 MHz, CDCl_3_) δ 9 (s, 1H), 7.76 – 7.62 (m, 4H), 6.92 (t, *J* = 9.2 Hz, 1H), 6.64 (dd, *J* = 12.7, 2.9 Hz, 1H), 6.57 (ddd, *J* = 9, 2.9, 1.4 Hz, 1H), 4.42 (d, *J* = 9.3 Hz, 1H), 3.97 (d, *J* = 9.2 Hz, 1H), 3.90 (s, 1H), 3.72 (s, 3H), 1.56 (s, 3H). ^19^F NMR (376 MHz, CDCl_3_) δ 86.57–84.10 (m, 1F), 63.39 (d, *J* = 150.3 Hz, 3F), –131.31 (s, 1F). ^13^C NMR (101 MHz, CDCl_3_) δ 172.68, 155.45, 155.36, 154.62, 152.18, 149.38, 139.98, 139.73, 139.62, 127.02, 126.97, 126.93, 119.04, 117.99, 117.97, 109.17, 109.15, 103.36, 103.14, 75.59, 75.39, 55.74, 22.97. HRMS(ESI)*m/z* calcd for C_17_H_17_F_6_NO_4_S [M + H]^+^: 446.0861, Found: 446.0818. [α]_D_^20^ = –3.9 (*c* = 0.8, MeOH).

### 3.4. Cell Preparation

CV-1 cells and C2C12 cells were both provided and certified by the Cell Bank at Shanghai Institutes for Biological Sciences, the Chinese Academy of Sciences, and confirmed as negative for mycoplasma contamination. CV-1 cells were cultured in phenol red-free LG-DMEM medium (Life Technologies, Carlsbad, CA, USA) supplemented with 10% (v/v) fetal bovine serum, 50 IU/mL penicillin, and 50 μg/mL streptomycin. C2C12 were cultured in phenol red-free HG-DMEM medium (Life Technologies, Carlsbad, CA, USA) supplemented with 10% (v/v) fetal bovine serum, 50 IU/mL penicillin, 50 μg/mL streptomycin, and 1% sodium pyruvate. Cells were maintained at 37 °C in a 5% CO_2_ incubator and seeded onto a 10 cm cell culture dish before transfection. After overnight culture, the cells were transiently co-transfected with AR expressing plasmid (pSVAR0) and luciferase reporter gene vector (MMTV-Luc) using FuGENE® HD Transfection Reagent (Promega, Madison, WI, USA) with cells reaching 80–90% confluence. After 18 h, the transfected cells were distributed to 384-well plates at a density of 15,000 cells per well and incubated for further 6 h at 37 °C before compound treatment.

### 3.5. Agonist Assay

DHT (Sigma, St. Louis, MO, USA) and ostarine were used as positive controls. The agonist activity of compounds was measured by Steadylite plus Reporter Gene Assay System (PerkinElmer, Boston, MA, USA) according to the manufacturer’s instructions. In brief, after incubation for 6 h, as mentioned above, 10 μL testing compounds diluted in culture medium with eight different working concentrations (384 pM to 30 μM; DHT (1 fM–1 μM); ostarine (32 pM–2.5 μM)) were added to the cell well (40 μL). After 24 h incubation, Steadylite reagent (50 μL, equal volume) was introduced, gently shook for 2 min, and kept at room temperature for 15 min before luminescence 384 measurement on an EnSpire multilabel plate reader (PerkinElmer, Boston, MA, USA).

### 3.6. Cytotoxicity

CellTiter-Glo® 2.0 assay (Promega, Madison, WI, USA) was applied to assess cytotoxicity. In brief, cells were seeded onto 384-well plates at a density of 1500 cells per well and incubated for 24 h. A total of 10 μL testing compounds diluted in culture medium were added and reacted for 24 h. CellTiter-Glo reagent was then introduced, and luminescence 384 was measured as above.

## 4. Conclusions

In summary, a small library of SF_5_-containing ostarine analogs instead of the CF_3_ group was synthesized, and their biological activity and SAR (structure-activity relationship), as SARMs, were studied. A series of *para*-SF_5_, *meta*-CN derivatives (**13a–g**) displayed different degrees of AR agonist activity. It appears that *mono*-SF_5_ substitution in the phenyl ring (**12a–g**, **16a–g**) resulted in a total loss of AR agonist activity. The derivatives containing the SF_5_ group maintained fewer AR agonist activities than the derivatives containing the CF_3_ group. The results pointed to the potential of using this derivative containing the SF_5_ group scaffold to develop new AR agonists.

## Figures and Tables

**Table 1:**
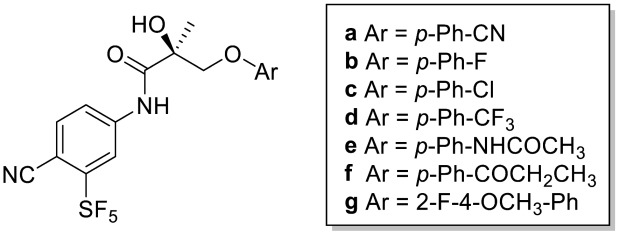
AR (androgen receptor) agonist activity of ostarine derivatives.

Compounds	Agonist Activity in CV-1		Agonist Activity in C2C12	
	EC_50_ (nM)	Efficacy (%)	EC_50_ (nM)	Efficacy (%)
DHT	3.1 ± 0.9	100	0.1 ± 0.03	100
Ostarine	1.7 ± 0.1	85.8 ± 7.4	3.9 ± 1.3	81.9 ± 11.6
**13a**	80.3 ± 74.5	89.5 ± 15.4	119.7 ± 109.9	80.8 ± 8.1
**13b**	28.1 ± 3.3	46.1 ± 11.3	42.1 ± 21.7	45.8 ± 21.9
**13c**	180.0 ± 116.0	66.3 ± 2.8 *	196.6 ± 110.0	69.9 ± 5.9 *
**13d**	1449.5 ± 99.7	51.2 ± 10.3	1035.8 ± 643.1	53.2 ± 7.9 *
**13e**	426.8 ± 387.8	65.1 ± 10.0	305.3 ± 161.7	68.4 ± 16.9
**13f**	15.1 ± 7.2	46.0 ± 5.2 *	66.9 ± 19.7	57.3 ± 16.3
**13g**	1135.0 ± 357.8	26.6 ± 15.2	921.4 ± 112.5	42.1 ± 6.9

Data presented are means ± SD of three independent experiments. EC_50_, half maximal effective concentration. * represents the efficiency at 6 μM as a result of their cytotoxicity at 30 μM.

**Table 2:**
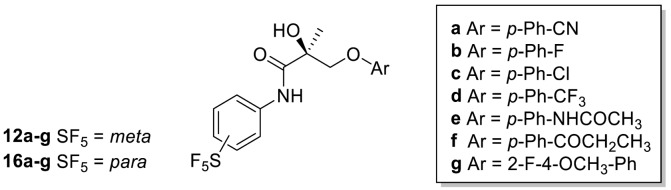
AR agonist activity of aryl propionamide derivatives.

Compounds	Agonist activity in CV-1		Compounds	Agonist activity in CV-1	
	EC_50_ (nM)	Efficacy (%)		EC_50_ (nM)	Efficacy (%)
**DHT**	3.1 ± 0.9	100	Ostarine	1.7 ± 0.1	85.8 ± 7.4
**12a**	1508 ± 429.9	9.1 ± 6.5	**16a**	NA	NA
**12b**	51213.5 ± 5806.1	5.2 ± 3.6	**16b**	NA	NA
**12c**	1282 ± 79.2	6.7 ± 4.9	**16c**	NA	NA
**12d**	NA	NA	**16d**	NA	NA
**12e**	NA	1.6 ± 1.4	**16e**	NA	NA
**12f**	12733.5 ± 2144.7	5.7 ± 9.5	**16f**	NA	0.7 ± 2.2
**12g**	NA	0.3 ± 1.6	**16g**	2144.0 ± 694.4	1.5 ± 0.5

Data presented are the means ± SD of three independent experiments. NA, not active.

## Supplementary Materials

The following are available online at https://www.mdpi.com/1420-3049/24/23/4227/s1, 1H, 13C, 19F NMR spectra, HPLC and HRMS of the synthesized compounds can be found in the Supporting Information.

## Abbreviations

SF_5_	Pentafluorosulfane
CF_3_	Trifluoromethyl
SARMs	Selective androgen receptor modulators
AR	Androgen receptor
DMAc	*N*,*N*-dimethylacetamide
DMF	*N*,*N*-dimethylformamide
DHT	Dihydrotestosterone
SAR	Structure-activity relationship
TMS	Tetramethylsilane
