# Supplementary material for: Synthesis of Aryl Propionamide Scaffold Containing a Pentafluorosulfanyl Moiety as SARMs
Source: Molecules. 2019 Nov 20;24(23):4227. doi: 10.3390/molecules24234227 (PMC6930600; doi:10.3390/molecules24234227)
Supplement: Supplementary file 1 [file molecules-24-04227-s001.pdf]

Supporting Information

Synthesis of Aryl Propionamide Scaffold Containing a Pentafluorosulfanyl Moiety as SARMs

Pingxuan Shao [1,†], Yan Zhou [2,†], Dehua Yang [2], Ming-Wei Wang [2,*], Wei Lu [1] and Jiyu Jin [1,*]

1 Shanghai Engineering Research Center of Molecular Therapeutics and New Drug Development, School of Chemistry and Molecular Engineering, East China Normal University, 3663 North Zhongshan Road, Shanghai 200062, China

2 The National Center for Drug Screening and the CAS Key Laboratory of Receptor Research, Shanghai Institute of Materia Medica, Chinese Academy of Sciences (CAS), 189 Guo Shou Jing Road, Shanghai, China

* Correspondence: mwwang@simm.ac.cn (M.-W.W.); jyjin@chem.ecnu.edu.cn (J.J.)

† These two authors contributed equally to this work.

Table of contents·····S 1
^{1}H, ^{13}C, ^{19}F NMR and MS spectra of **2**·····S 3
^{1}H, ^{13}C, ^{19}F NMR and MS spectra of **3**·····S 5
^{1}H and ^{13}C NMR spectra of **7**·····S 7
^{1}H and ^{13}C NMR spectra of **8**·····S 8
^{1}H, ^{13}C, ^{19}F NMR and MS spectra of **9**·····S 9
^{1}H, ^{13}C, ^{19}F NMR and MS spectra of **10**·····S 11
^{1}H, ^{13}C, ^{19}F NMR and MS spectra of **15**·····S 13
^{1}H, ^{13}C, ^{19}F NMR, HPLC and HRMS spectra of **12a**·····S 15
^{1}H, ^{13}C, ^{19}F NMR, HPLC and HRMS spectra of **12b**·····S 18
^{1}H, ^{13}C, ^{19}F NMR, HPLC and HRMS spectra of **12c**·····S 21
^{1}H, ^{13}C, ^{19}F NMR, HPLC and HRMS spectra of **12d**·····S 24
^{1}H, ^{13}C, ^{19}F NMR, HPLC and HRMS spectra of **12e**·····S 27
^{1}H, ^{13}C, ^{19}F NMR, HPLC and HRMS spectra of **12f**·····S 30
^{1}H, ^{13}C, ^{19}F NMR, HPLC and HRMS spectra of **12g**·····S 33
^{1}H, ^{13}C, ^{19}F NMR, HPLC and HRMS spectra of **13a**·····S 36
^{1}H, ^{13}C, ^{19}F NMR, HPLC and HRMS spectra of **13b**·····S 39
^{1}H, ^{13}C, ^{19}F NMR, HPLC and HRMS spectra of **13c**·····S 42
^{1}H, ^{13}C, ^{19}F NMR, HPLC and HRMS spectra of **13d**·····S 45
^{1}H, ^{13}C, ^{19}F NMR, HPLC and HRMS spectra of **13e**·····S 48
^{1}H, ^{13}C, ^{19}F NMR, HPLC and HRMS spectra of **13f**·····S 51
^{1}H, ^{13}C, ^{19}F NMR, HPLC and HRMS spectra of **13g**·····S 54
^{1}H, ^{13}C, ^{19}F NMR, HPLC and HRMS spectra of **16a**·····S 57
^{1}H, ^{13}C, ^{19}F NMR, HPLC and HRMS spectra of **16b**·····S 60
^{1}H, ^{13}C, ^{19}F NMR, HPLC and HRMS spectra of **16c**·····S 63
^{1}H, ^{13}C, ^{19}F NMR, HPLC and HRMS spectra of **16d**·····S 66
^{1}H, ^{13}C, ^{19}F NMR, HPLC and HRMS spectra of **16e**·····S 69
^{1}H, ^{13}C, ^{19}F NMR, HPLC and HRMS spectra of **16f**·····S 72
^{1}H, ^{13}C, ^{19}F NMR, HPLC and HRMS spectra of **16g**·····S 75

^{1}H, ^{13}C, ^{19}F NMR and MS spectra of *compound* **2**

^{1}H NMR (400 MHz, $CDCl_3$) δ 7.45 (d, J = 8.5 Hz, 1H), 7.13 (d, J = 2.3 Hz, 1H), 6.61 (dd, J = 8.5, 1.8 Hz, 1H), 3.89 (s, 2H).

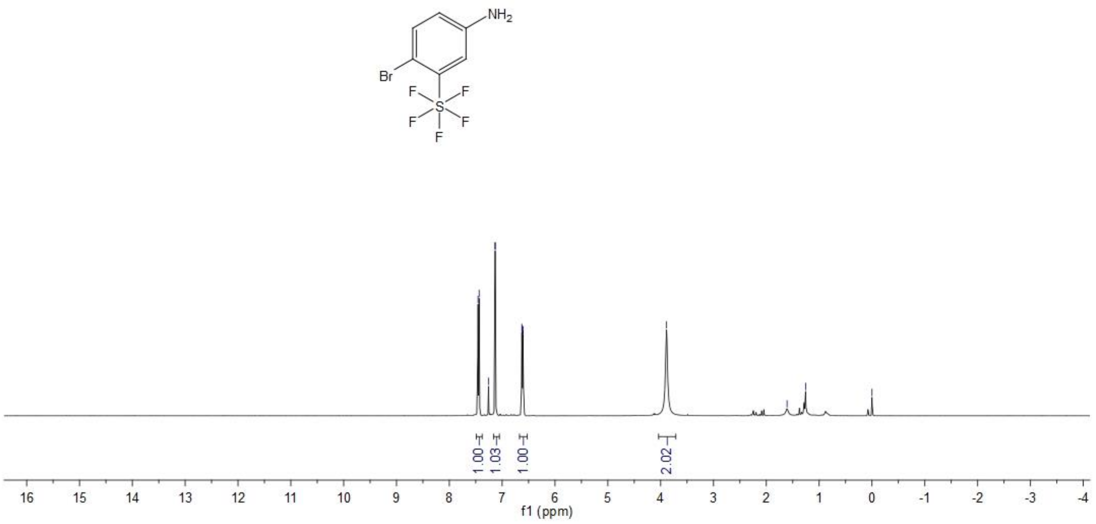

^{13}C NMR (101 MHz, $CDCl_3$) δ 154.32, 154.15, 153.98, 145.52, 136.59, 118.89, 116.06, 116.01, 115.96, 115.90, 104.15.

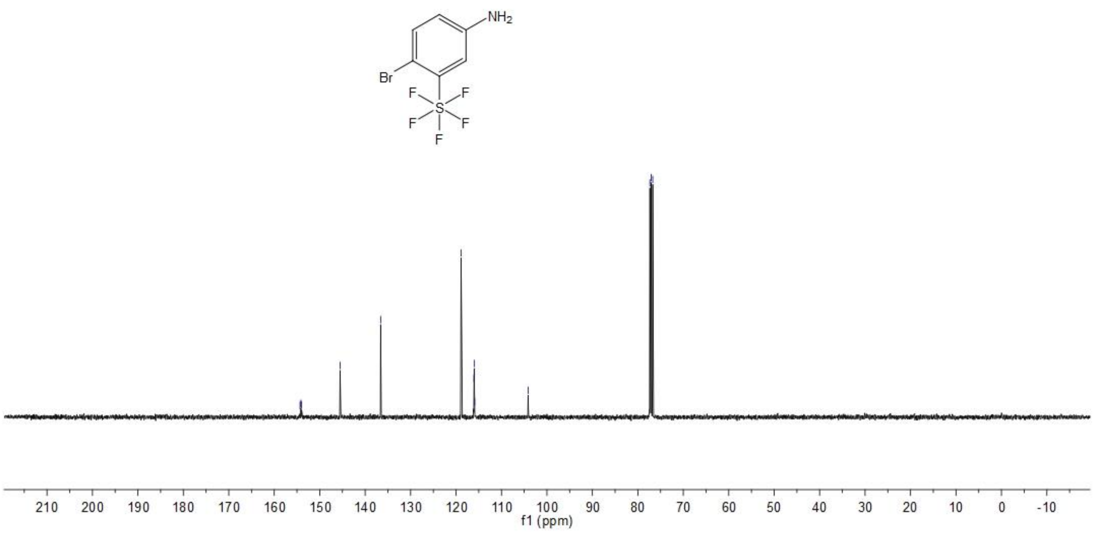

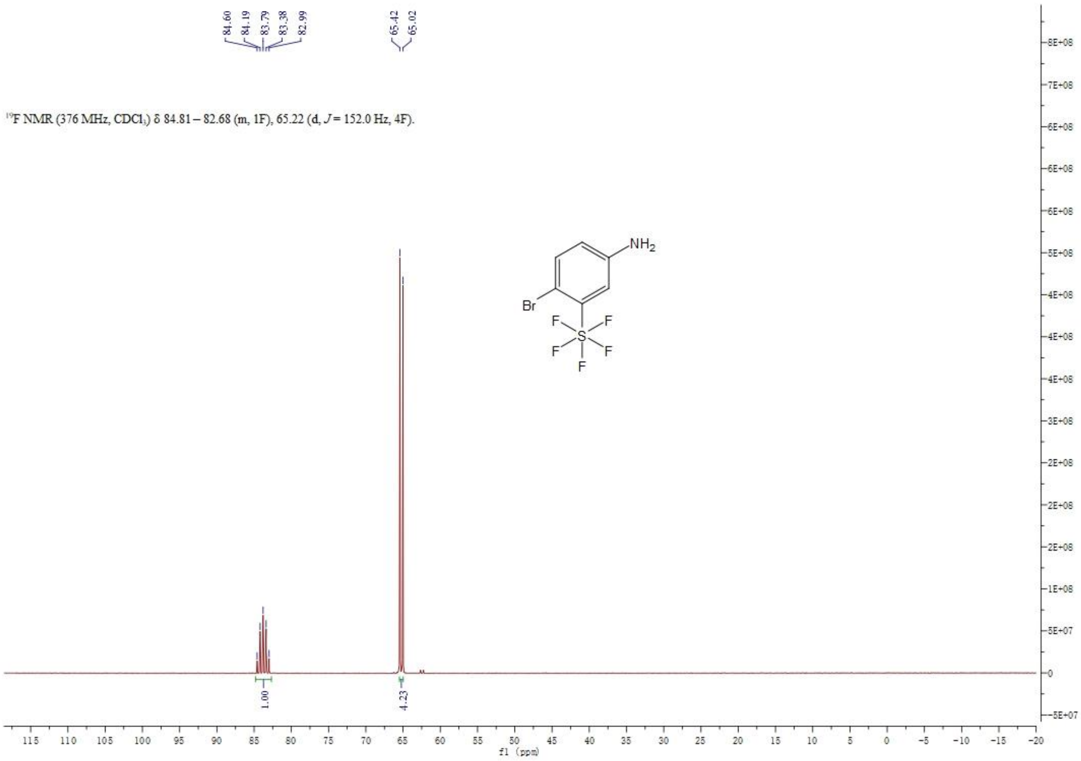
84.60
84.19
83.79
83.38
82.99
65.42
65.02
^{19}F NMR (376 MHz, $CDCl_3$) δ 84.81 – 82.68 (m, 1F), 65.22 (d, J = 152.0 Hz, 4F).
NH_2
Br
F
S
1.00
4.23
f1 (ppm)

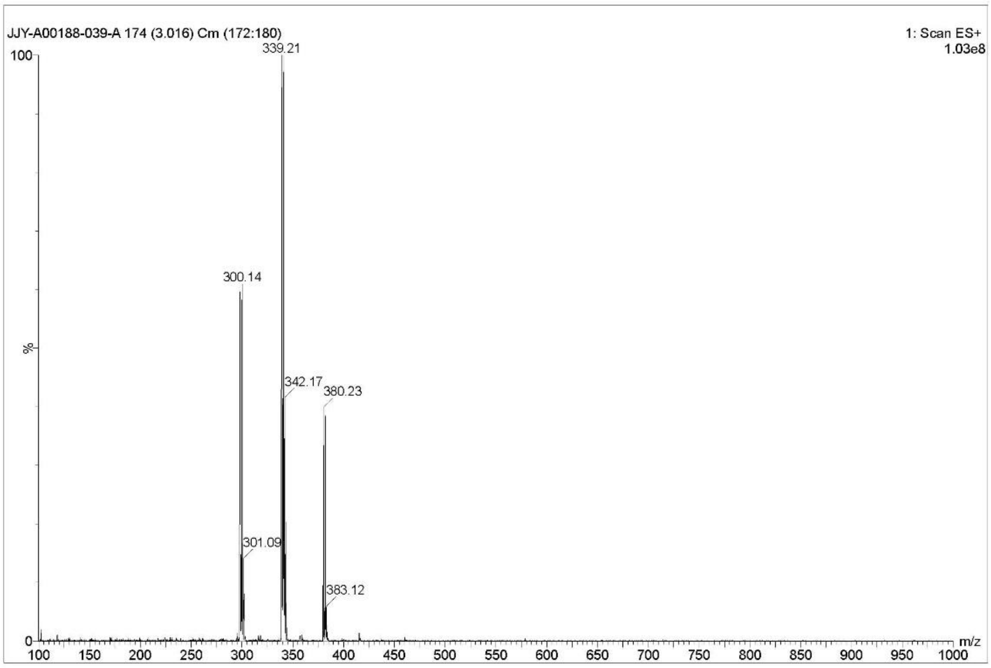
JJY-A00188-039-A 174 (3.016) Cm (172:180)
1: Scan ES+
1.03e8
339.21
300.14
342.17
380.23
301.09
383.12
%
m/z

^{1}H, ^{13}C, ^{19}F NMR and MS spectra of *compound* ***3***

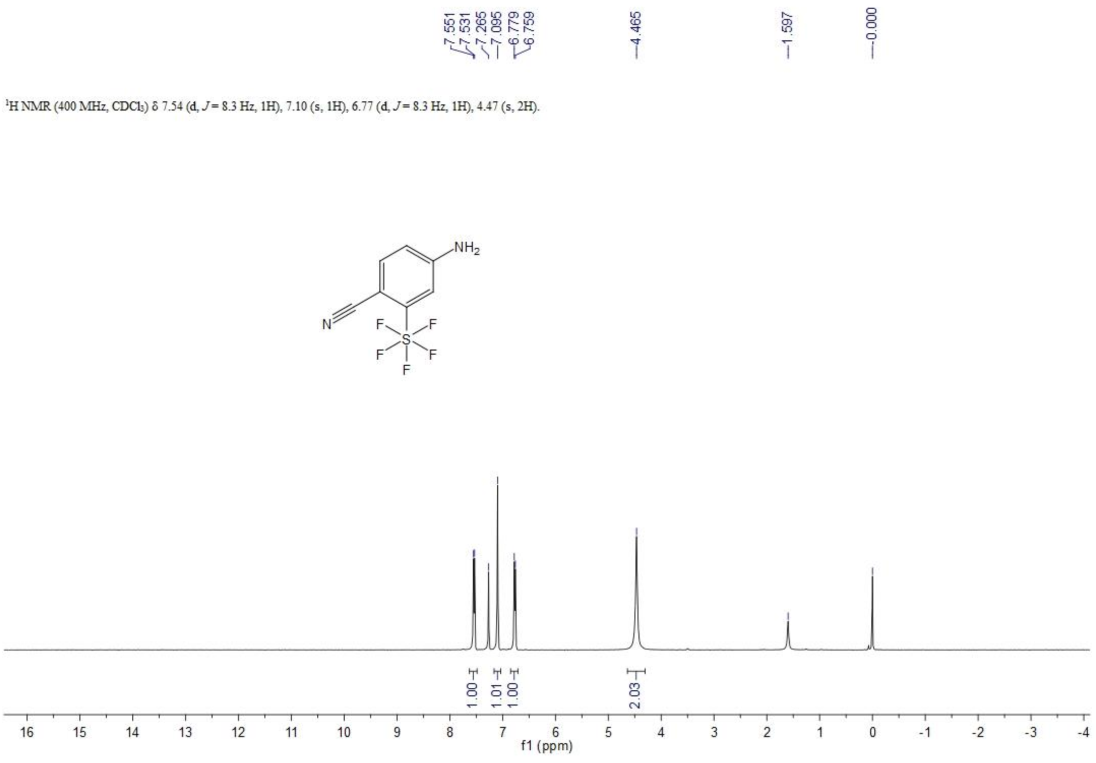

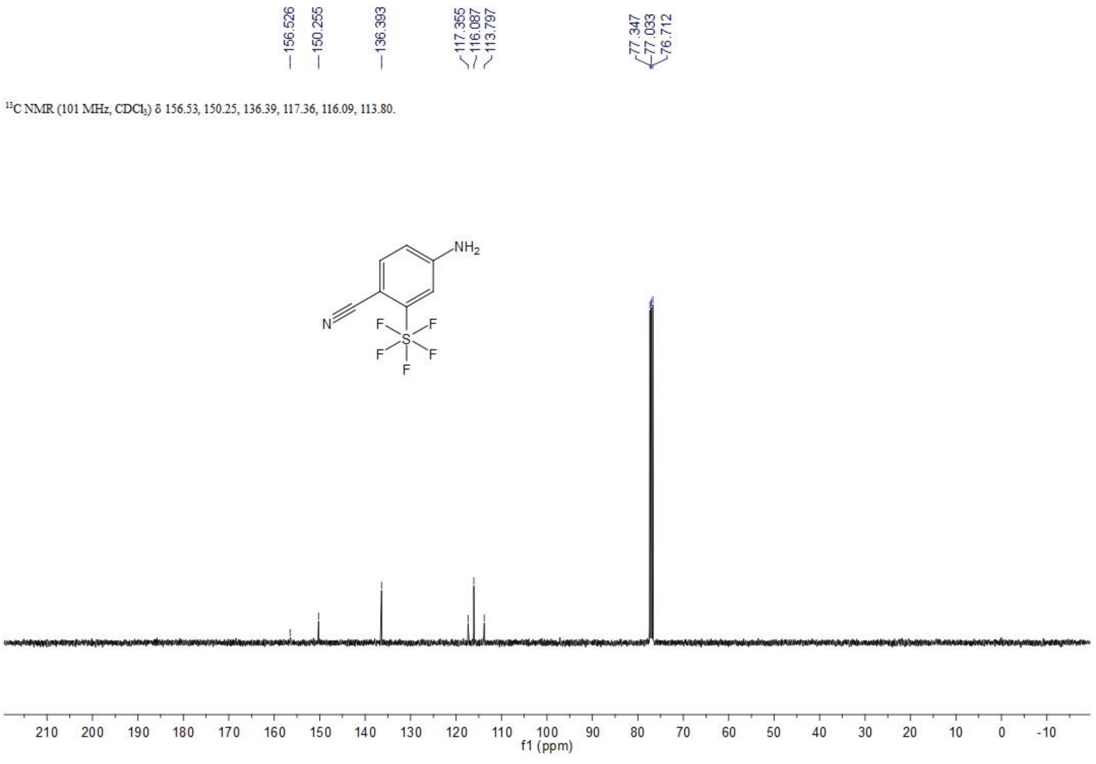

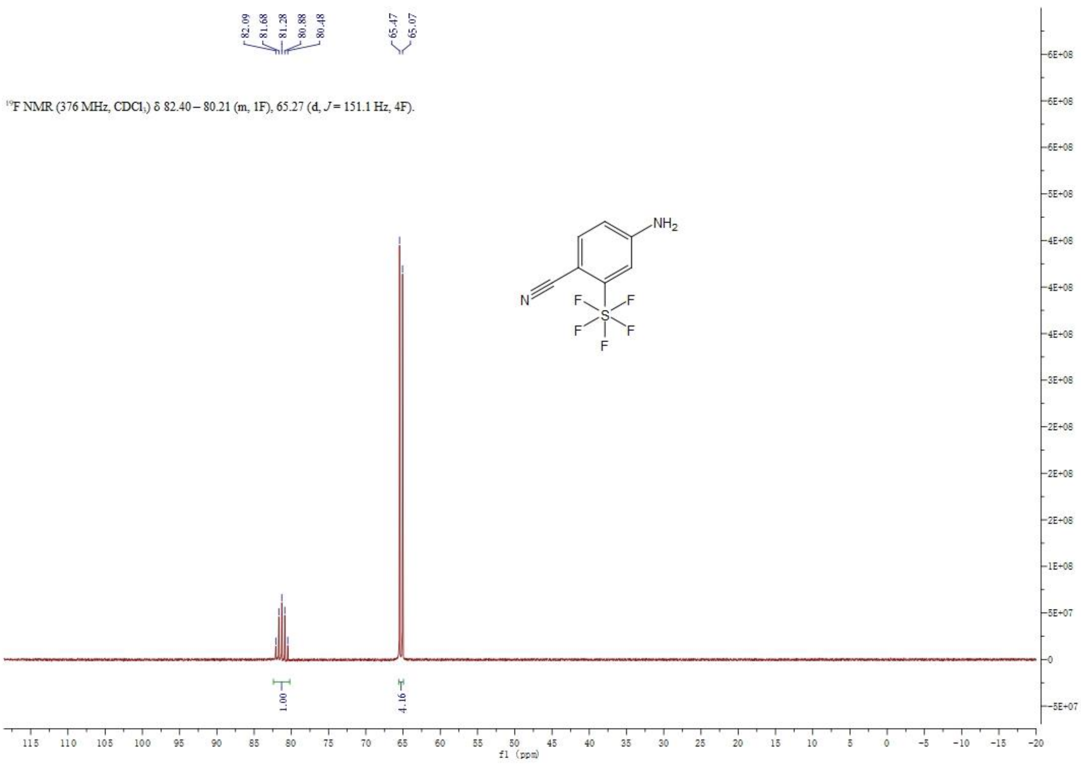
82.09
81.68
81.28
80.88
80.48
65.47
65.07
^{19}F NMR (376 MHz, CDCl$_3$) δ 82.40 – 80.21 (m, 1F), 65.27 (d, J = 151.1 Hz, 4F).
NH$_2$
N
F
S
F
F
F
F
1.00
4.16
f1 (ppm)

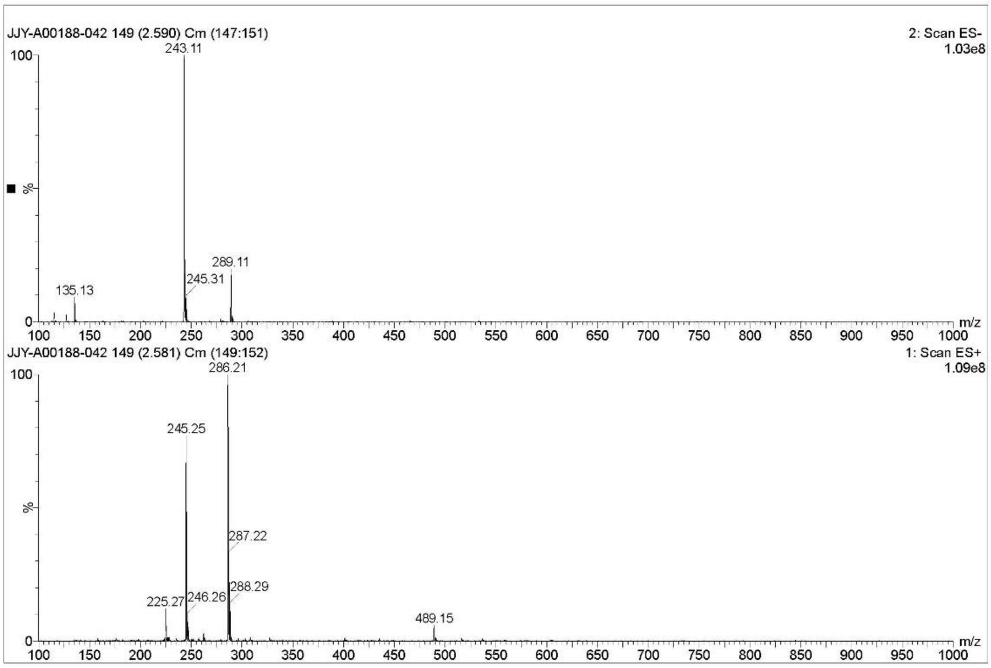
JJY-A00188-042 149 (2.590) Cm (147:151)
2: Scan ES-
1.03e8
243.11
289.11
245.31
135.13
m/z
JJY-A00188-042 149 (2.581) Cm (149:152)
1: Scan ES+
1.09e8
286.21
245.25
287.22
288.29
225.27
246.26
489.15
m/z

¹H and ¹³C NMR spectra of *compound* ***7***

^{1}H NMR (400 MHz, DMSO) δ 4.77 – 4.66 (m, 1H), 4.03 (d, *J* = 11.5 Hz, 1H), 3.87 (d, *J* = 11.3 Hz, 1H), 3.52 (dd, *J* = 18.7, 9.2 Hz, 1H), 3.40 (t, *J* = 10.3 Hz, 1H), 2.27 (dd, *J* = 17.3, 6.7 Hz, 1H), 1.95 (dt, *J* = 17.5, 8.0 Hz, 2H), 1.82 (dd, *J* = 19.0, 10.4 Hz, 1H), 1.58 (s, 3H).

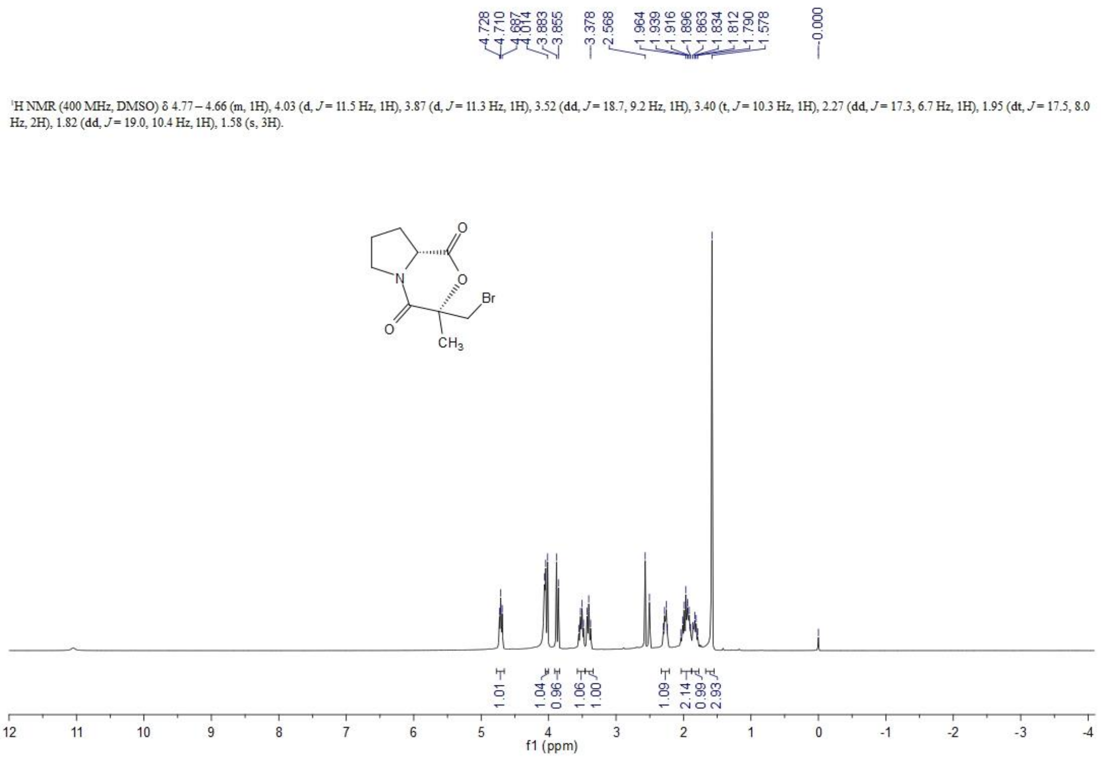

^{13}C NMR (101 MHz, DMSO) δ 167.26, 163.07, 83.88, 57.22, 45.41, 37.82, 29.02, 22.92, 21.58.

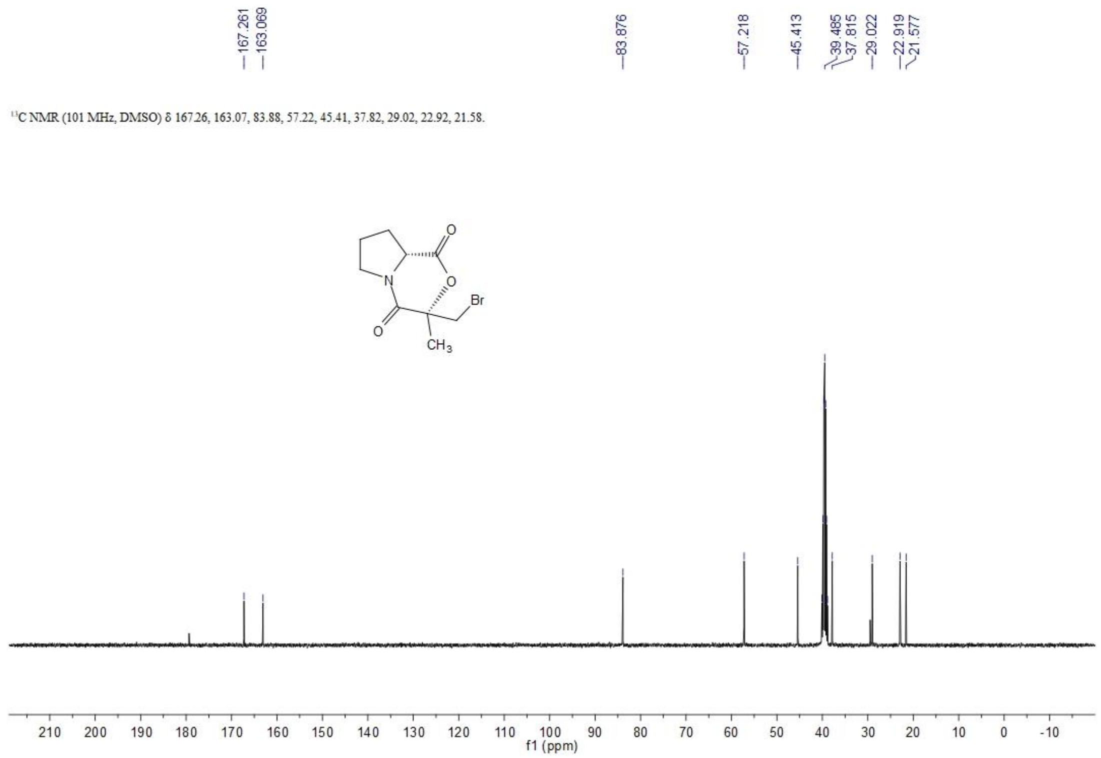

^{1}H and ^{13}C NMR spectra of *compound* **8**

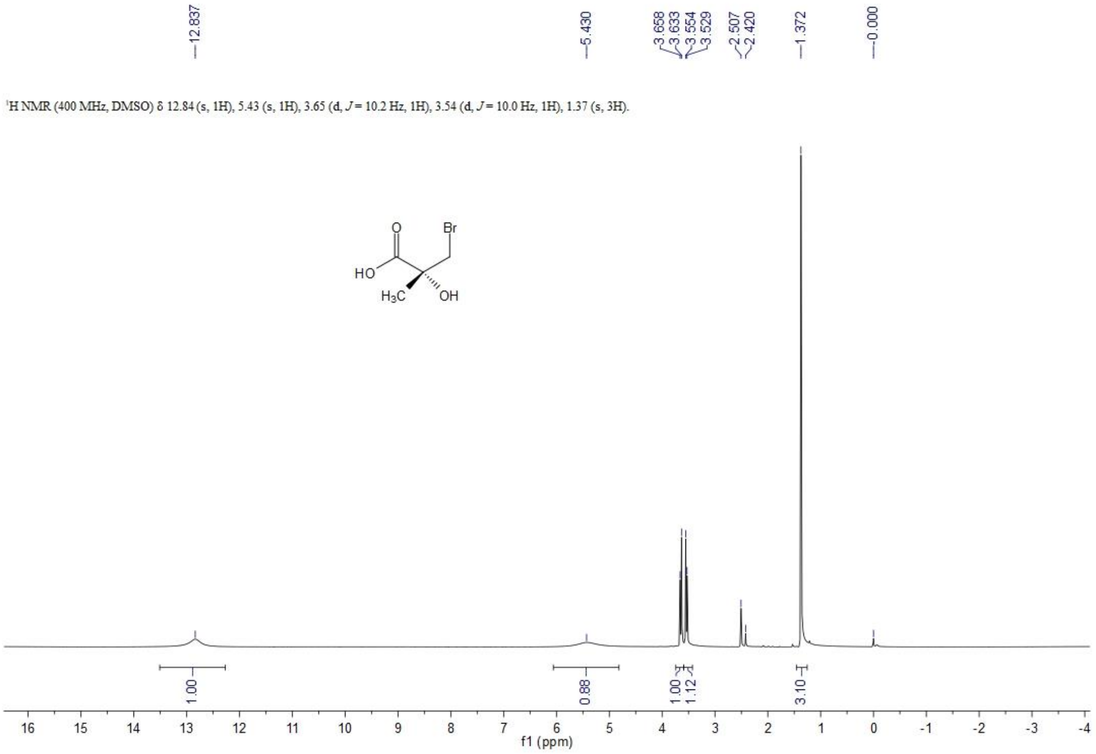

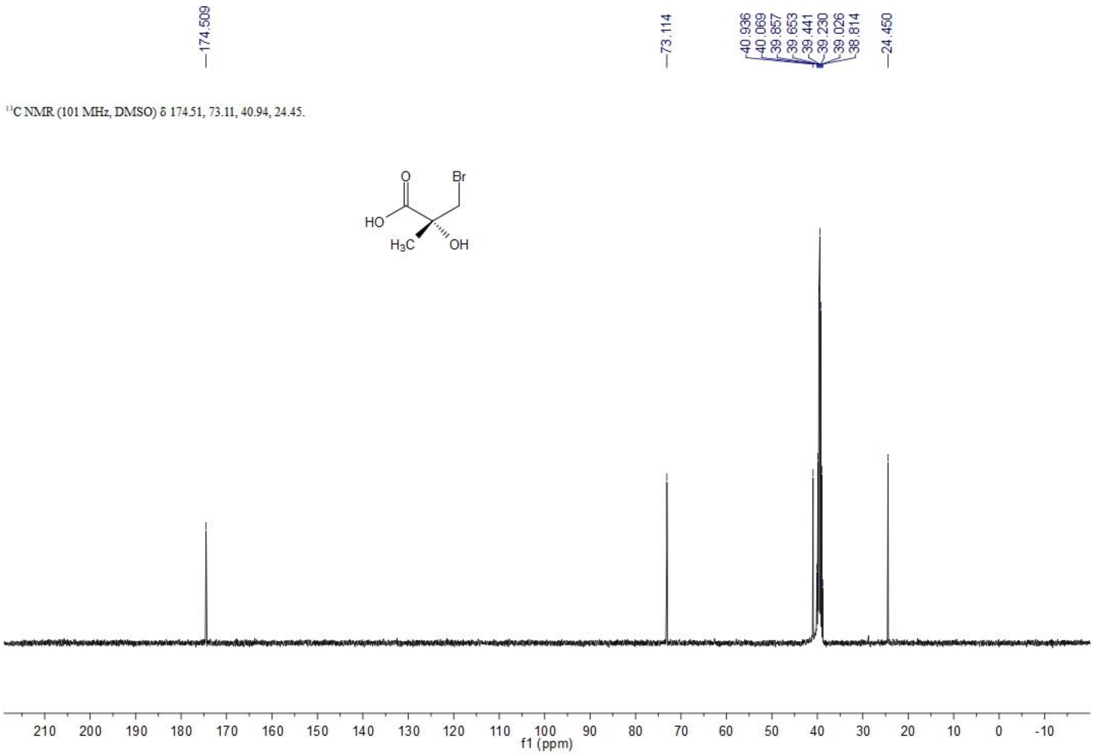

^{1}H, ^{13}C, ^{19}F NMR and MS spectra of *compound* **9**

^{1}H NMR (400 MHz, $CDCl_3$) δ 8.77 (s, 1H), 8.03 (s, 1H), 7.74 (d, *J* = 7.7 Hz, 1H), 7.51 (d, *J* = 8.0 Hz, 1H), 7.42 (t, *J* = 7.9 Hz, 1H), 4.01 (d, *J* = 10.4 Hz, 1H), 3.59 (d, *J* = 10.4 Hz, 1H), 3.01 (s, 1H), 1.61 (s, 3H).

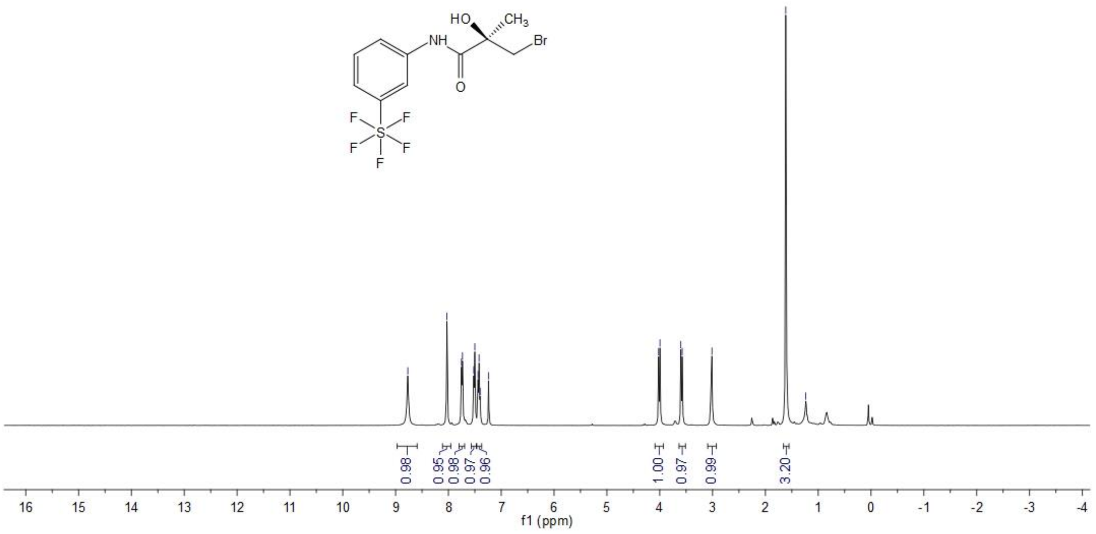

^{13}C NMR (101 MHz, $CDCl_3$) δ 171.12, 154.28, 137.42, 129.28, 122.63, 122.04, 117.46, 75.39, 41.50, 24.79.

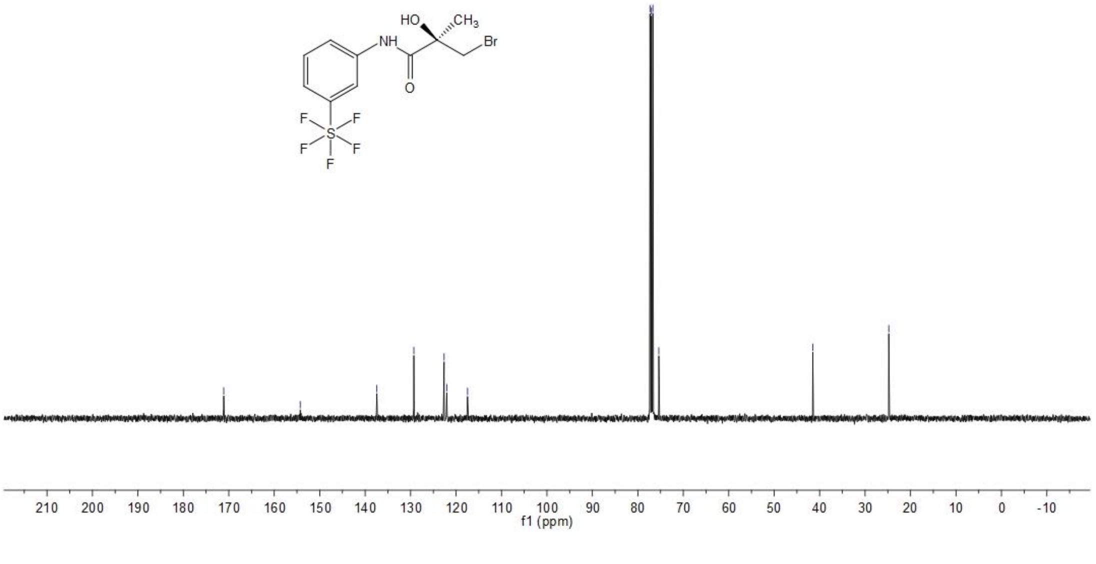

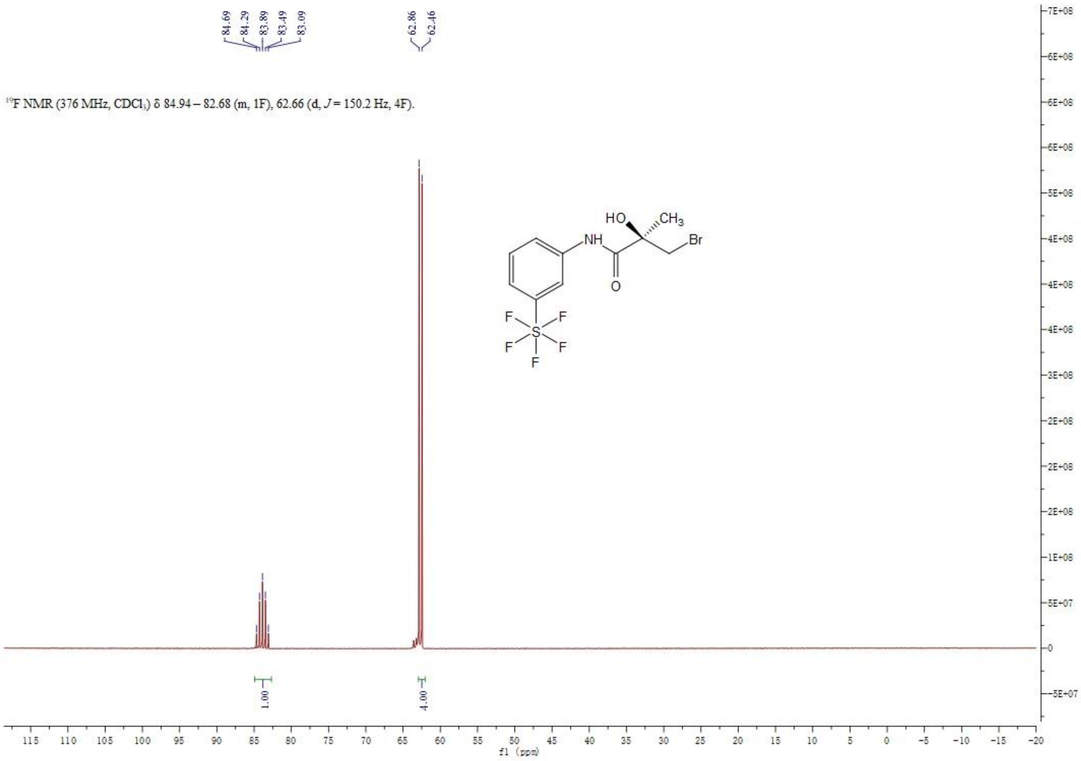

84.69
84.29
83.89
83.49
83.09
62.86
62.46
^{19}F NMR (376 MHz, CDCl3) δ 84.94 – 82.68 (m, 1F), 62.66 (d, J = 150.2 Hz, 4F).
HO
CH3
NH
Br
O
F
S
1.00
4.00
f1 (ppm)

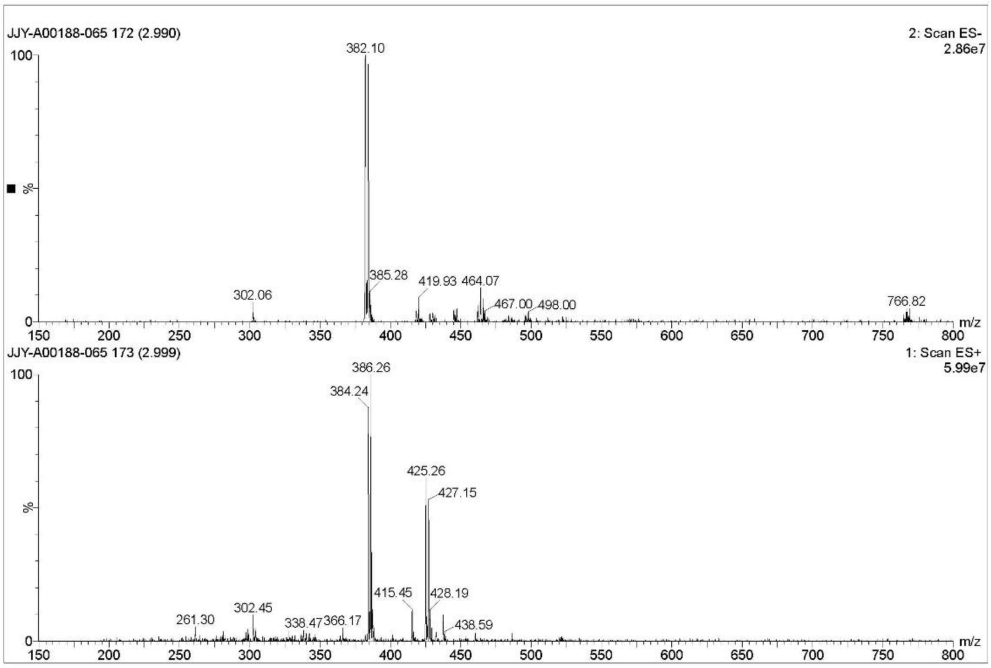

JJY-A00188-065 172 (2.990)
2: Scan ES-
2.86e7
382.10
385.28
419.93
464.07
467.00
498.00
302.06
766.82
m/z
%
JJY-A00188-065 173 (2.999)
1: Scan ES+
5.99e7
386.26
384.24
425.26
427.15
415.45
428.19
438.59
302.45
261.30
338.47
366.17
m/z

^{1}H, ^{13}C, ^{19}F NMR and MS spectra of *compound* **10**

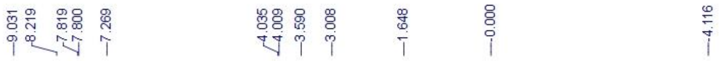

^{1}H NMR (400 MHz, $CDCl_3$) δ 9.03 (s, 1H), 8.22 (s, 1H), 7.97 (d, J = 8.3 Hz, 1H), 7.81 (d, J = 7.9 Hz, 1H), 4.02 (d, J = 10.5 Hz, 1H), 3.60 (d, J = 10.5 Hz, 1H), 3.01 (s, 1H), 1.65 (s, 3H).

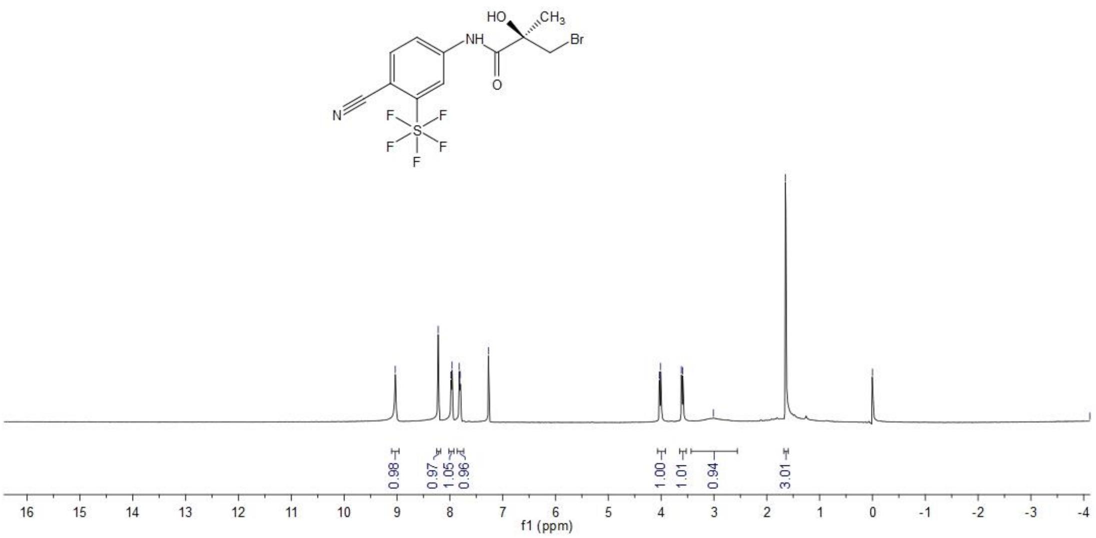

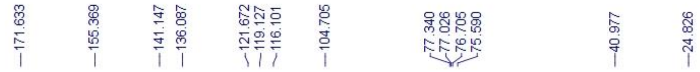

^{13}C NMR (101 MHz, $CDCl_3$) δ 171.63, 155.37, 141.15, 136.09, 121.67, 119.13, 116.10, 104.70, 75.59, 40.98, 24.83.

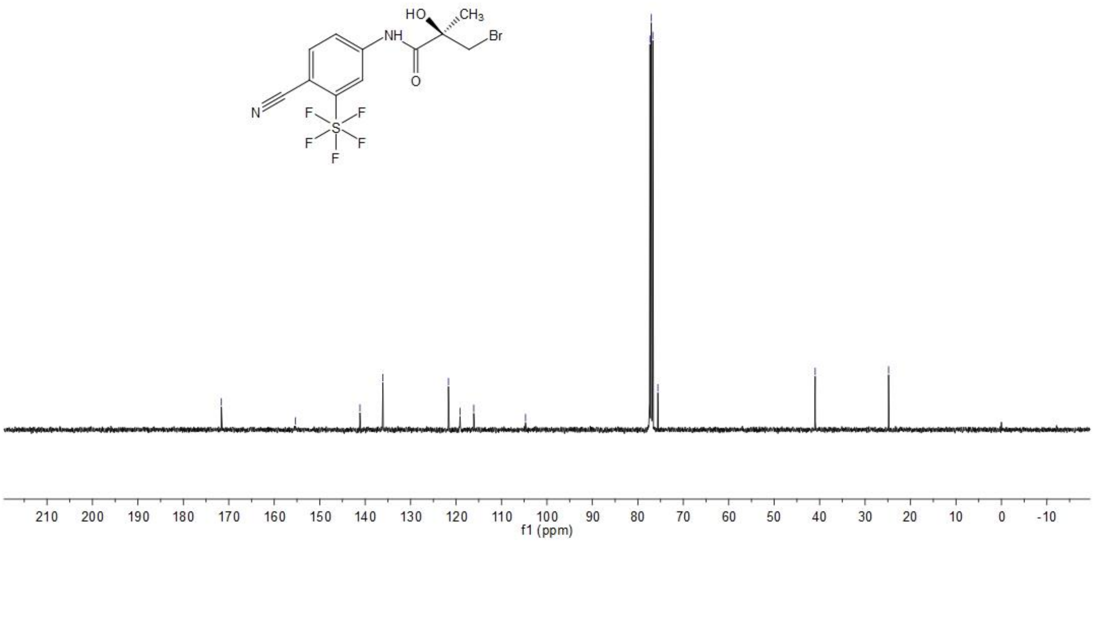

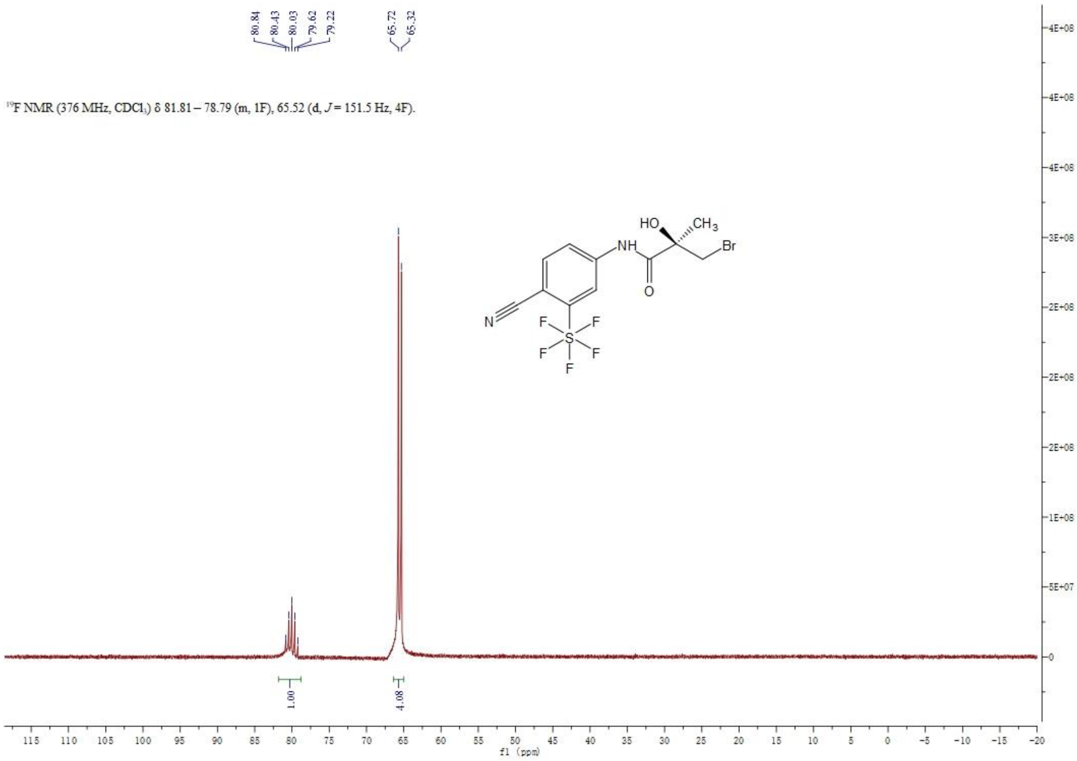
80.84
80.43
80.03
79.62
79.22
65.72
65.32
19F NMR (376 MHz, CDCl3) δ 81.81 – 78.79 (m, 1F), 65.52 (d, J = 151.5 Hz, 4F).
HO
CH3
NH
Br
O
N
F
S
1.00
4.08
f1 (ppm)

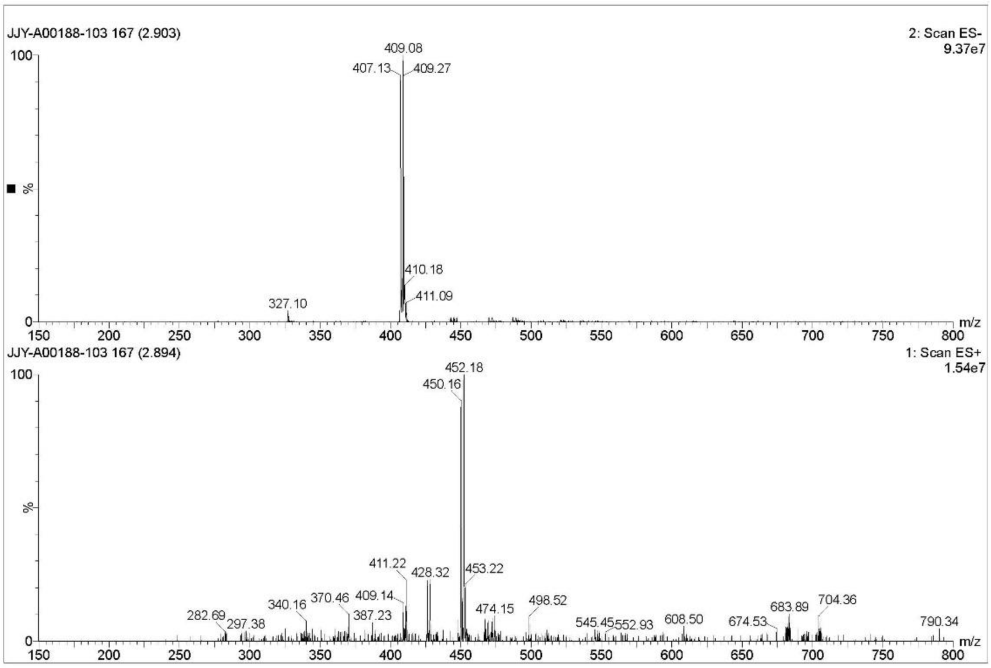
JJY-A00188-103 167 (2.903)
2: Scan ES-
9.37e7
409.08
407.13
409.27
410.18
411.09
327.10
%
m/z
JJY-A00188-103 167 (2.894)
1: Scan ES+
1.54e7
452.18
450.16
411.22
428.32
453.22
340.16
370.46
409.14
387.23
282.69
297.38
474.15
498.52
545.45
552.93
608.50
674.53
683.89
704.36
790.34
%
m/z

^{1}H, ^{13}C, ^{19}F NMR and MS spectra of *compound* **15**

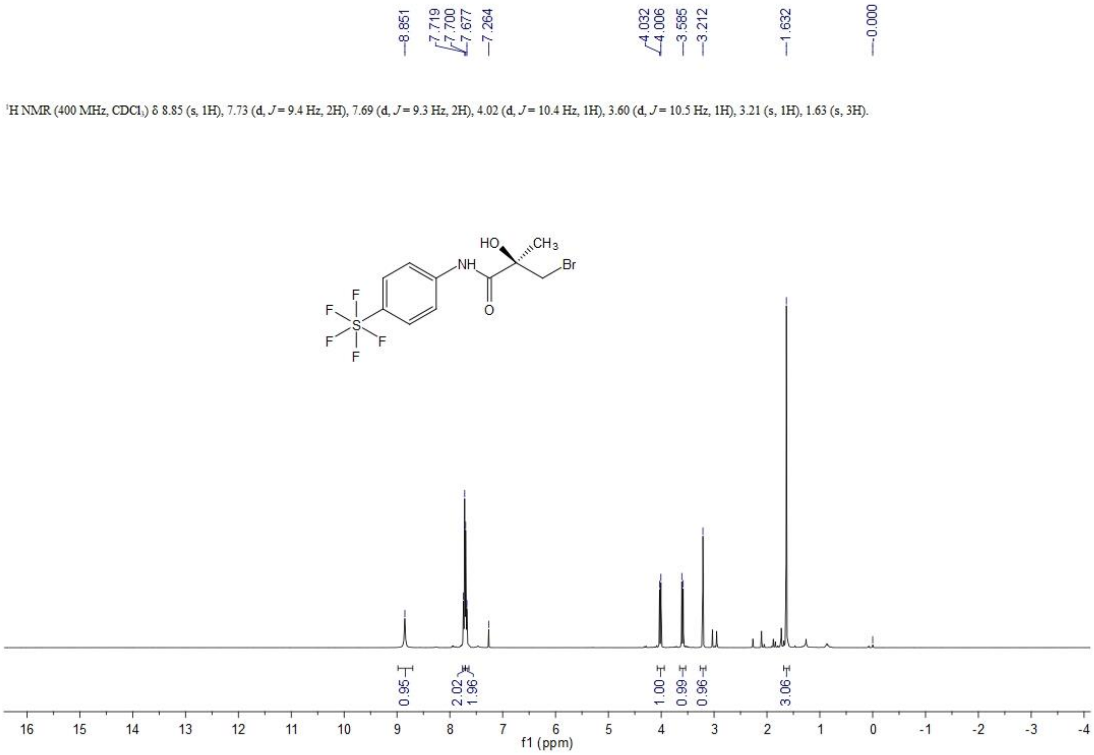

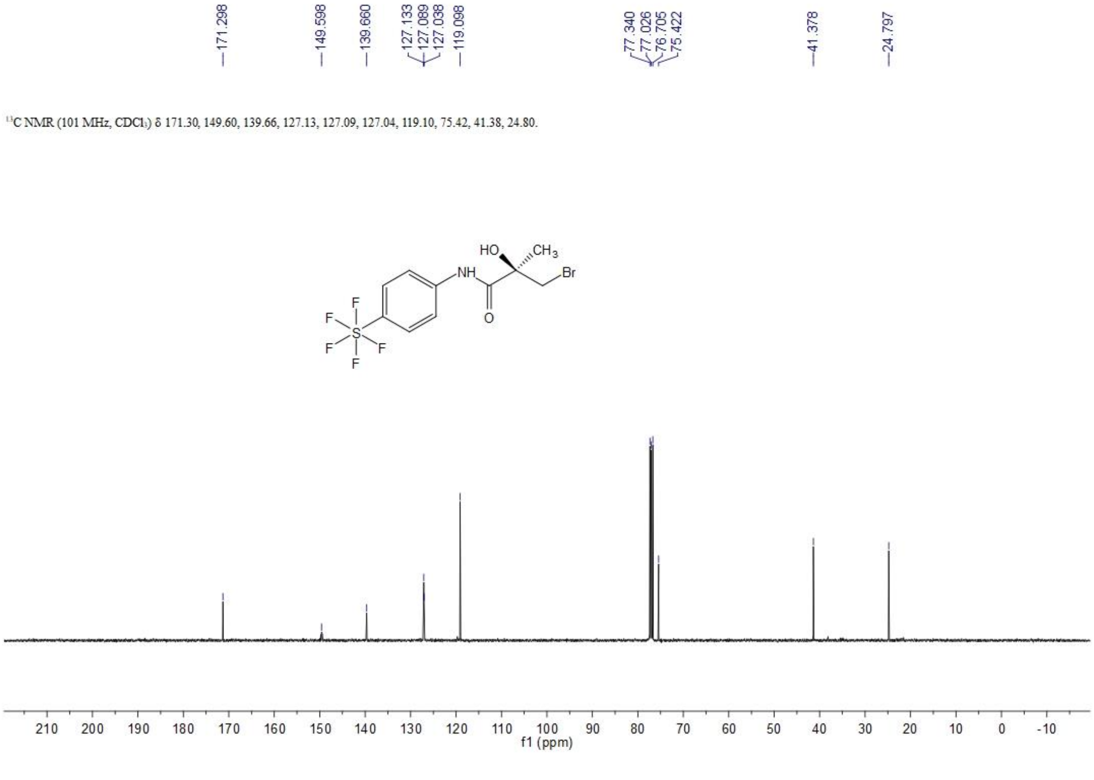

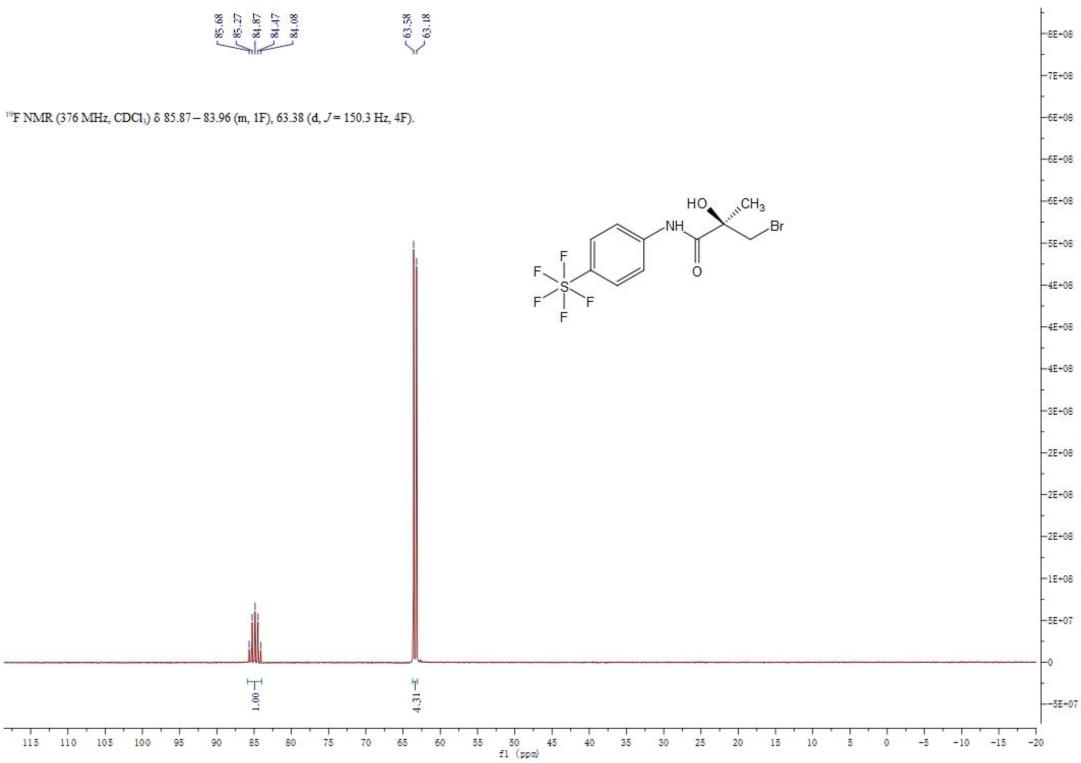

85.68
85.27
84.87
84.47
84.08
63.58
63.18
^{19}F NMR (376 MHz, CDCl$_3$) δ 85.87 – 83.96 (m, 1F), 63.38 (d, J = 150.3 Hz, 4F).
1.00
4.31
f1 (ppm)

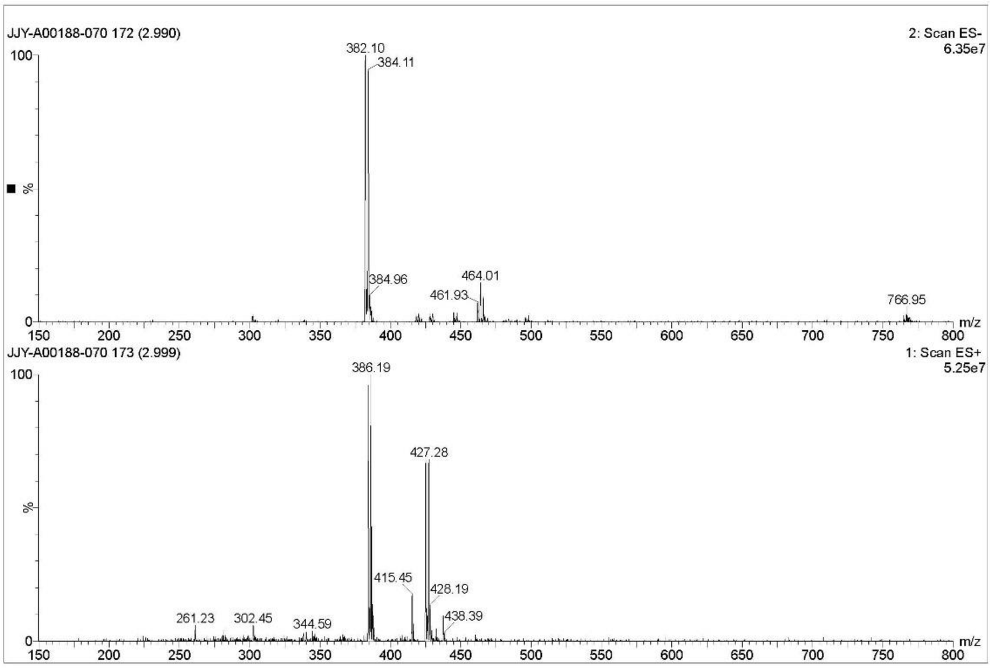

JJY-A00188-070 172 (2.990)
2: Scan ES-
6.35e7
382.10
384.11
384.96
464.01
461.93
766.95
m/z
JJY-A00188-070 173 (2.999)
1: Scan ES+
5.25e7
386.19
427.28
415.45
428.19
438.39
261.23
302.45
344.59
m/z

^{1}H, ^{13}C, ^{19}F NMR, HPLC and HRMS spectra of *compound* **12a**

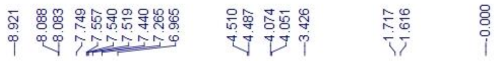

^{1}H NMR (400 MHz, $CDCl_3$) δ 8.92 (s, 1H), 8.09 (t, *J* = 2.0 Hz, 1H), 7.76 (d, *J* = 8.0 Hz, 1H), 7.57 (d, *J* = 8.8 Hz, 2H), 7.53 (dd, *J* = 8.4, 1.3 Hz, 1H), 7.44 (t, *J* = 8.2 Hz, 1H), 6.98 (d, *J* = 8.9 Hz, 2H), 4.50 (d, *J* = 9.2 Hz, 1H), 4.06 (d, *J* = 9.2 Hz, 1H), 3.43 (s, 1H), 1.62 (s, 3H).

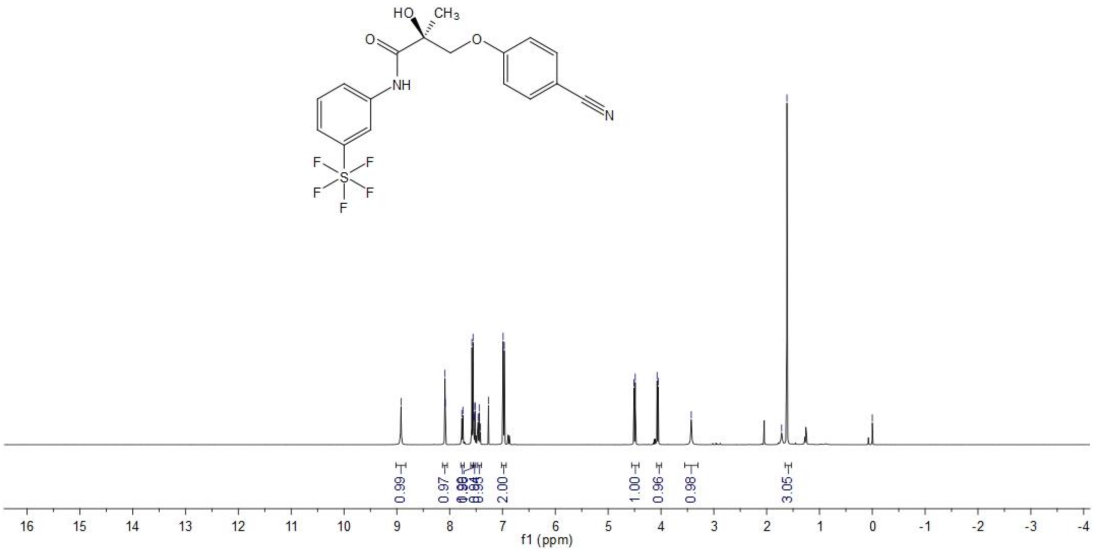

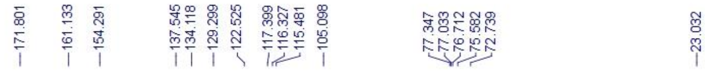

^{13}C NMR (101 MHz, $CDCl_3$) δ 171.80, 161.13, 154.29, 137.55, 134.12, 129.30, 122.52, 122.00, 118.80, 117.40, 116.33, 115.48, 105.10, 75.58, 72.74, 23.03.

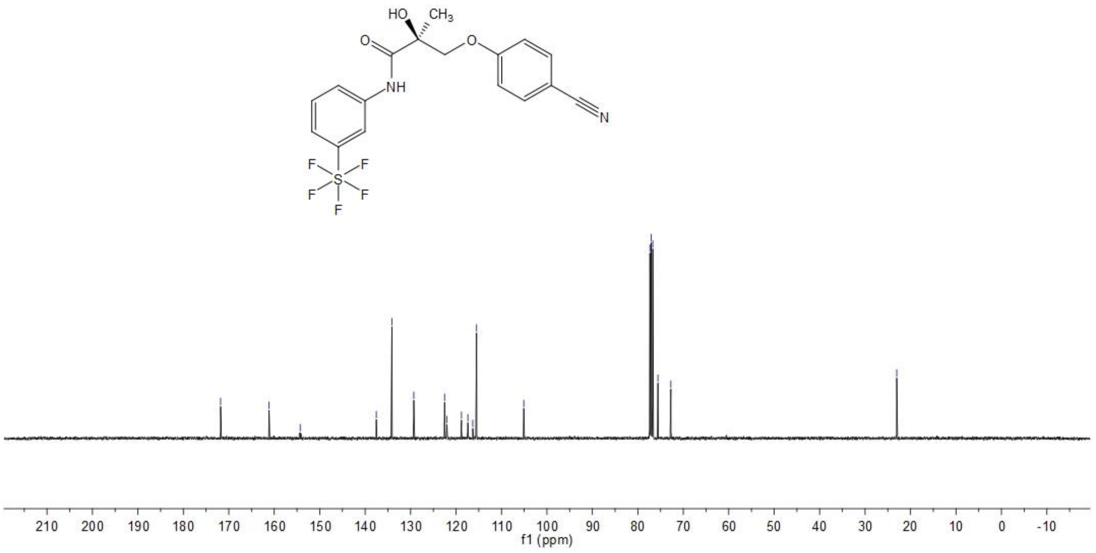

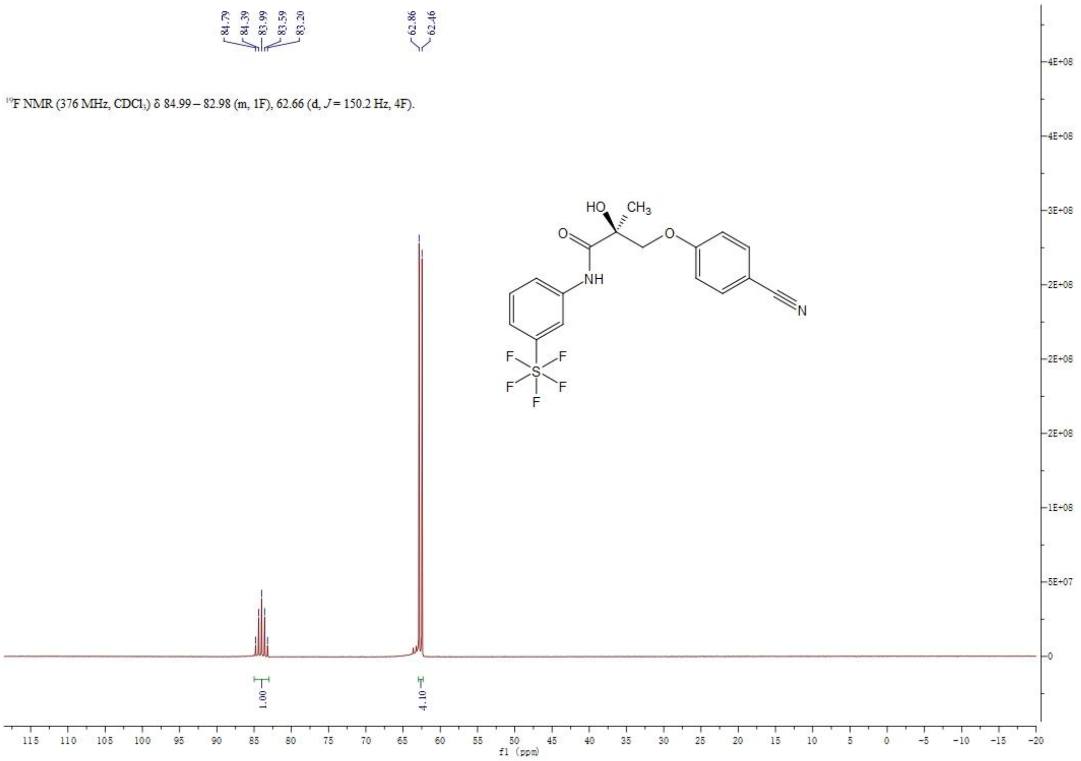

Elemental Composition Report

Page 1

Single Mass Analysis

Tolerance = 100.0 PPM / DBE: min = -1.5, max = 50.0
Element prediction: Off

Monoisotopic Mass, Even Electron Ions
1 formula(e) evaluated with 1 results within limits (up to 50 closest results for each mass)
Elements Used:
C: 17-17 H: 16-16 N: 2-2 O: 3-3 F: 5-5 S: 1-1
JJY-A00188-067-5 36 (0.727)
1: TOF MS ES+

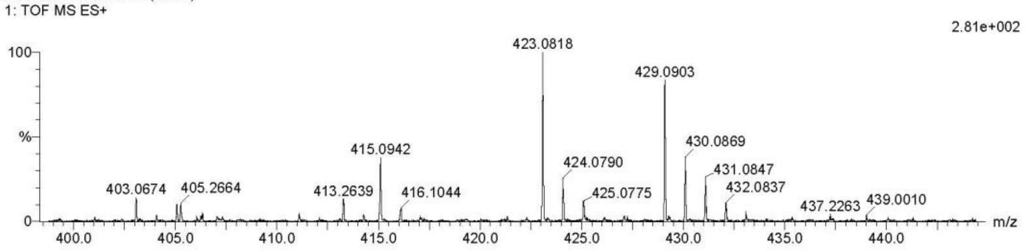

Minimum:				-1.5	
Maximum:		5.0	100.0	50.0	
Mass	Calc. Mass	mDa	PPM	DBE	Formula
423.0818	423.0802	1.6	3.8	8.5	C17 H16 N2 O3 F5 S

数据文件：C:\CHEM32\1\DATA\JJY\LW000040.D
样品名称：JJY-A00188-067

===

操作者 : spx
仪器 : 仪器 1 位置 : 样品瓶 1
进样日期 : 2019/11/14 17:19:21
进样量 : 没有进样
采集方法 : C:\CHEM32\1\METHODS\JJY-15MIN.M
最后修改 : 2019/11/14 17:17:02 : spx
(调用后修改)
分析方法 : C:\CHEM32\1\METHODS\JJY-15MIN.M
最后修改 : 2019/11/4 16:29:42 : CYT
附加信息：峰已手动积分

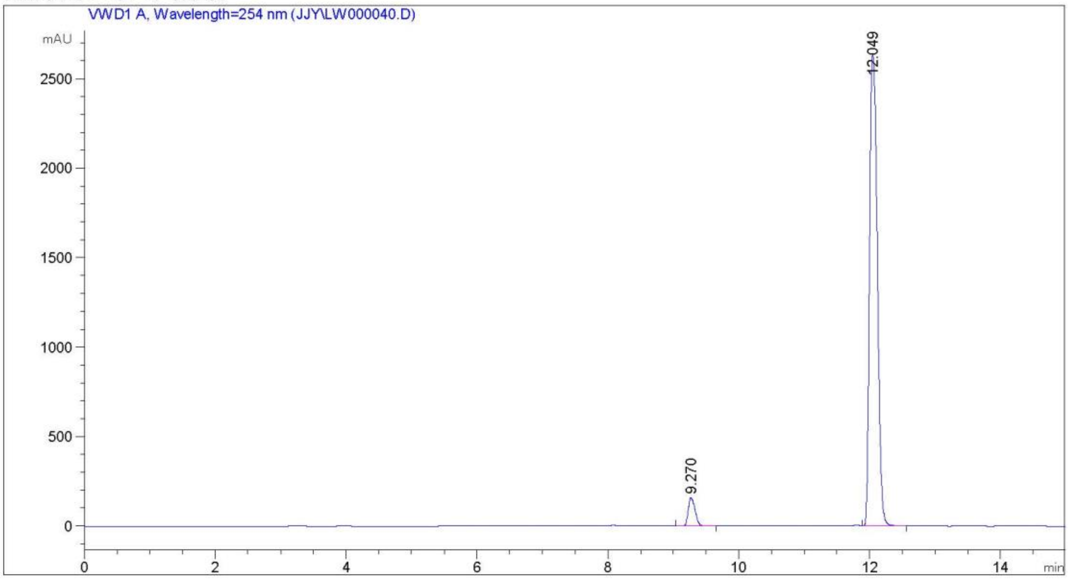

===

面积百分比报告

===

排序 : 信号
乘积因子: : 1.0000
稀释因子: : 1.0000
内标使用乘积因子和稀释因子

信号 1: VWD1 A, Wavelength=254 nm

峰 #	保留时间 [min]	类型	峰宽 [min]	峰面积 [mAU*s]	峰高 [mAU]	峰面积 %
1	9.270	BB	0.1147	1124.54578	158.61719	4.9355
2	12.049	VB	0.1337	2.16604e4	2635.83203	95.0645

总量 : 2.27849e4 2794.44922

===

^{1}H, ^{13}C, ^{19}F NMR, HPLC and HRMS spectra of *compound* **12b**

^{1}H NMR (400 MHz, $CDCl_3$) δ 8.94 (s, 1H), 8.08 (t, J = 1.9 Hz, 1H), 7.74 (d, J = 8.2 Hz, 1H), 7.50 (dd, J = 8.3, 1.2 Hz, 1H), 7.40 (t, J = 8.2 Hz, 1H), 6.94 (dt, J = 11.9, 3.0 Hz, 2H), 6.87 – 6.78 (m, 2H), 4.40 (d, J = 9.0 Hz, 1H), 3.94 (d, J = 9.0 Hz, 1H), 3.58 (s, 1H), 1.57 (s, 3H).

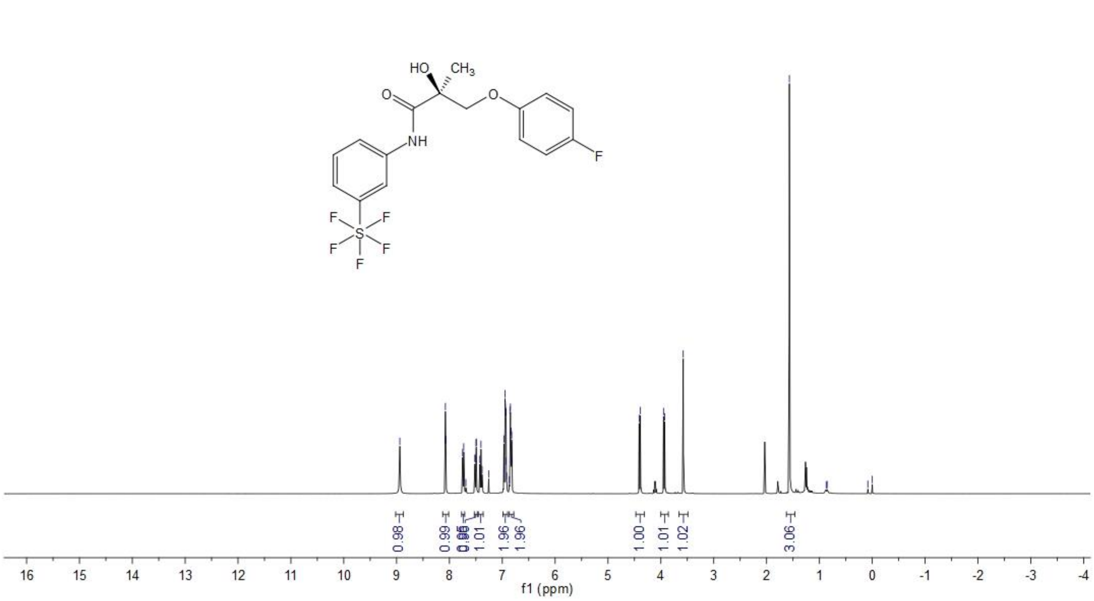

^{13}C NMR (101 MHz, $CDCl_3$) δ 172.39, 159.06, 156.67, 153.98, 137.67, 129.23, 122.54, 121.85, 117.38, 116.14, 116.06, 115.98, 115.90, 75.62, 73.32, 22.94.

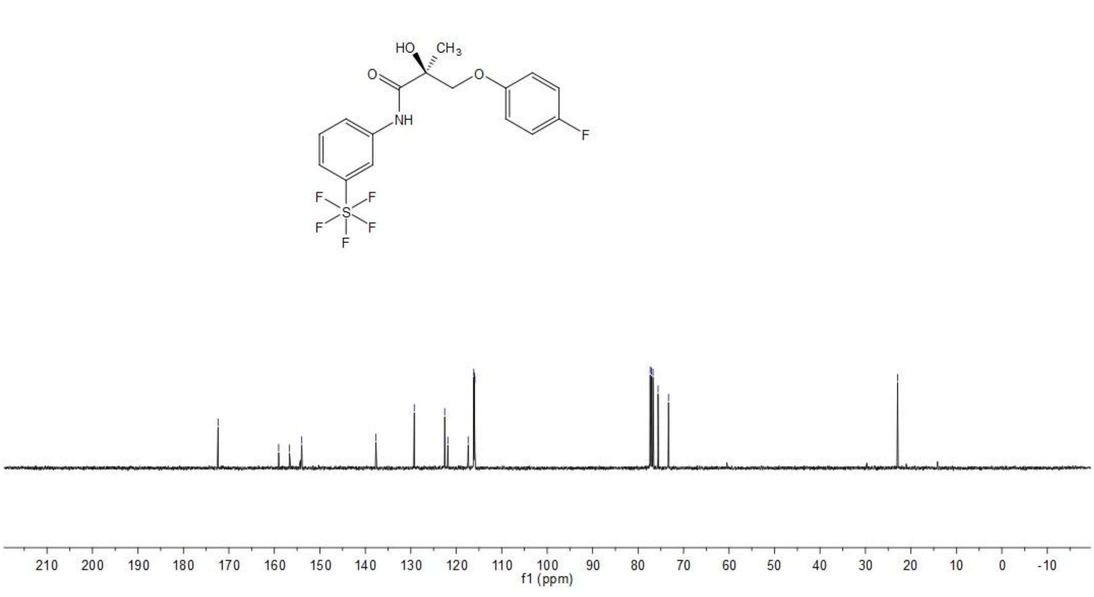

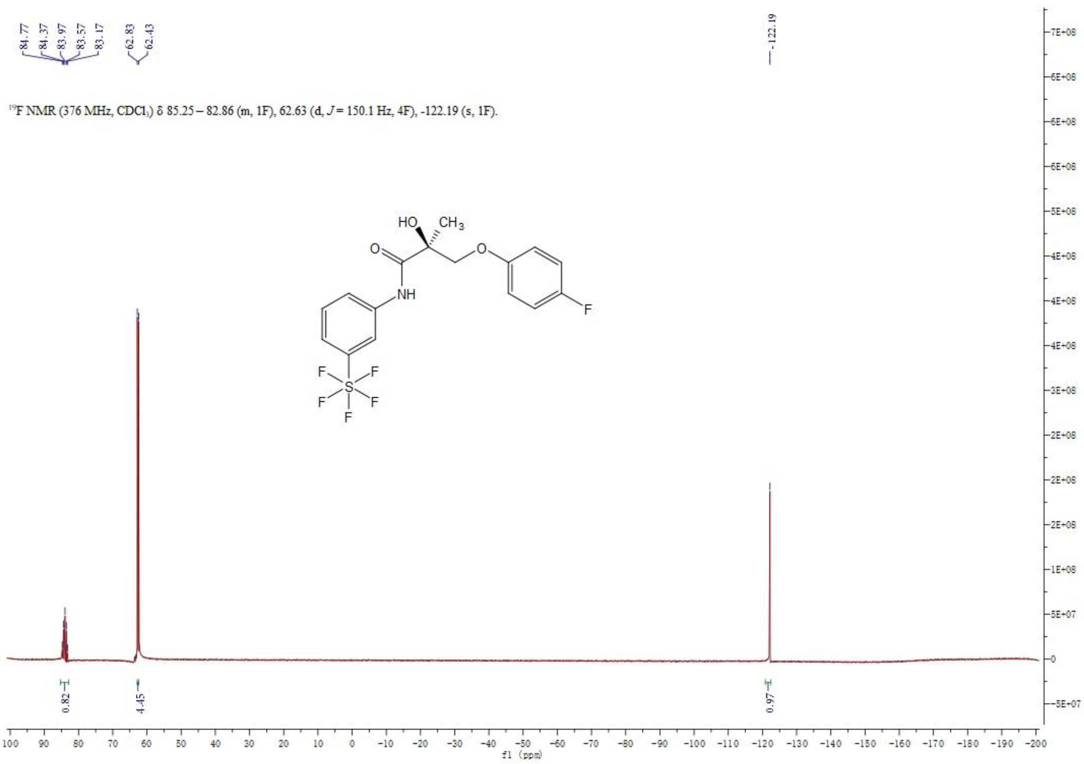

Elemental Composition Report

Page 1

Single Mass Analysis

Tolerance = 100.0 PPM / DBE: min = -1.5, max = 50.0
Element prediction: Off

Monoisotopic Mass, Even Electron Ions
1 formula(e) evaluated with 1 results within limits (up to 50 closest results for each mass)
Elements Used:
C: 16-16 H: 16-16 N: 1-1 O: 3-3 F: 6-6 S: 1-1
JJY-A00188-088 30 (0.605)
1: TOF MS ES+

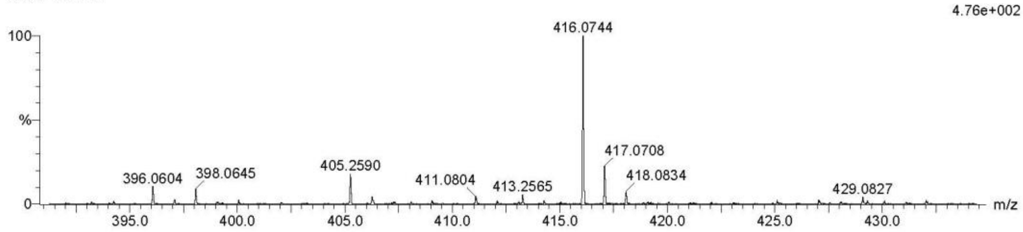

Mass	Calc. Mass	mDa	PPM	DBE	Formula
Minimum:				-1.5	
Maximum:		5.0	100.0	50.0	
416.0744	416.0755	-1.1	-2.6	6.5	C16 H16 N O3 F6 S

Data File D:\AGILENT DATA\DATA\JJY\JJY20160203 2016-03-17 13-17-41\JJY0000002.D
Sample Name: JJY-A00188-088

===

Acq. Operator : JJY Seq. Line : 2
Acq. Instrument : Instrument 1 Location : Vial 82
Injection Date : 3/17/2016 1:40:56 PM Inj : 1
Inj Volume : 10.0 µl
Acq. Method : D:\AGILENT DATA\DATA\JJY\JJY20160203 2016-03-17 13-17-41\JJY-0.1TFA-CH3CN-15MIN.M
Last changed : 3/17/2016 12:29:03 PM by JJY
Analysis Method : D:\AGILENT DATA\METHOD\JJY-0.1TFA-CH3CN-15MIN.M
Last changed : 3/17/2016 12:29:03 PM by JJY
Additional Info : Peak(s) manually integrated

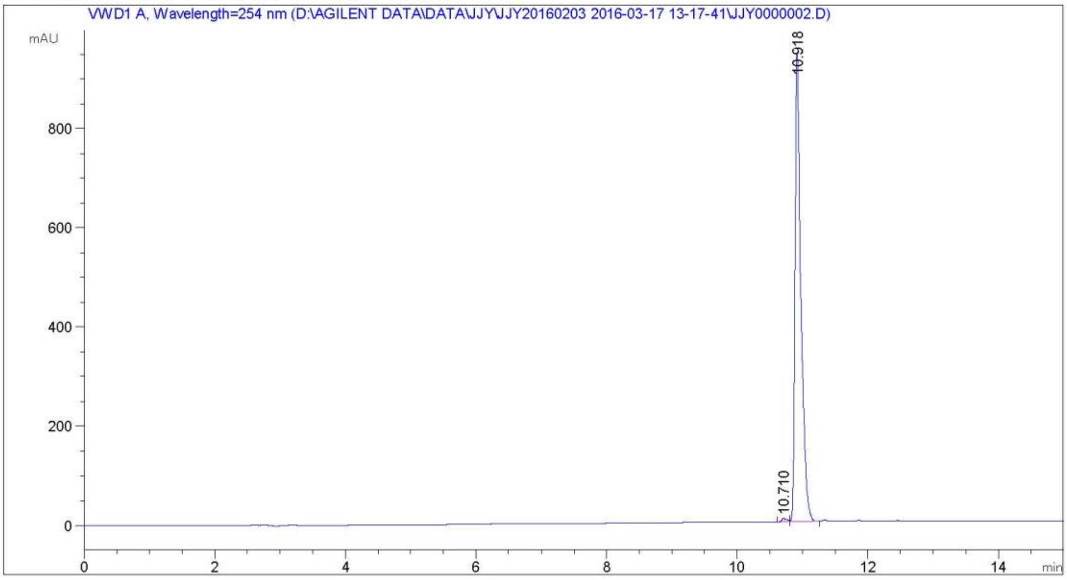

===

Area Percent Report

===

Sorted By : Signal
Multiplier : 1.0000
Dilution : 1.0000
Use Multiplier & Dilution Factor with ISTDs

Signal 1: VWD1 A, Wavelength=254 nm

Peak #	RetTime [min]	Type	Width [min]	Area mAU *s	Height [mAU]	Area %
1	10.710	BV	0.0880	42.52554	7.03860	0.7185
2	10.918	VB	0.0886	5876.13037	944.83765	99.2815

Totals : 5918.65591 951.87624

===

Instrument 1 3/17/2016 2:10:06 PM JJY Page 1 of 1

^{1}H, ^{13}C, ^{19}F NMR, HPLC and HRMS spectra of *compound* **12c**

^{1}H NMR (400 MHz, $CDCl_3$) δ 8.93 (s, 1H), 8.08 (t, *J* = 2.0 Hz, 1H), 7.72 (d, *J* = 8.0 Hz, 1H), 7.50 (dd, *J* = 8.3, 1.4 Hz, 1H), 7.40 (t, *J* = 8.2 Hz, 1H), 7.23 – 7.17 (m, 2H), 6.86 – 6.78 (m, 2H), 4.39 (d, *J* = 9.0 Hz, 1H), 3.94 (d, *J* = 9.2 Hz, 1H), 3.58 (s, 1H), 1.57 (s, 3H).

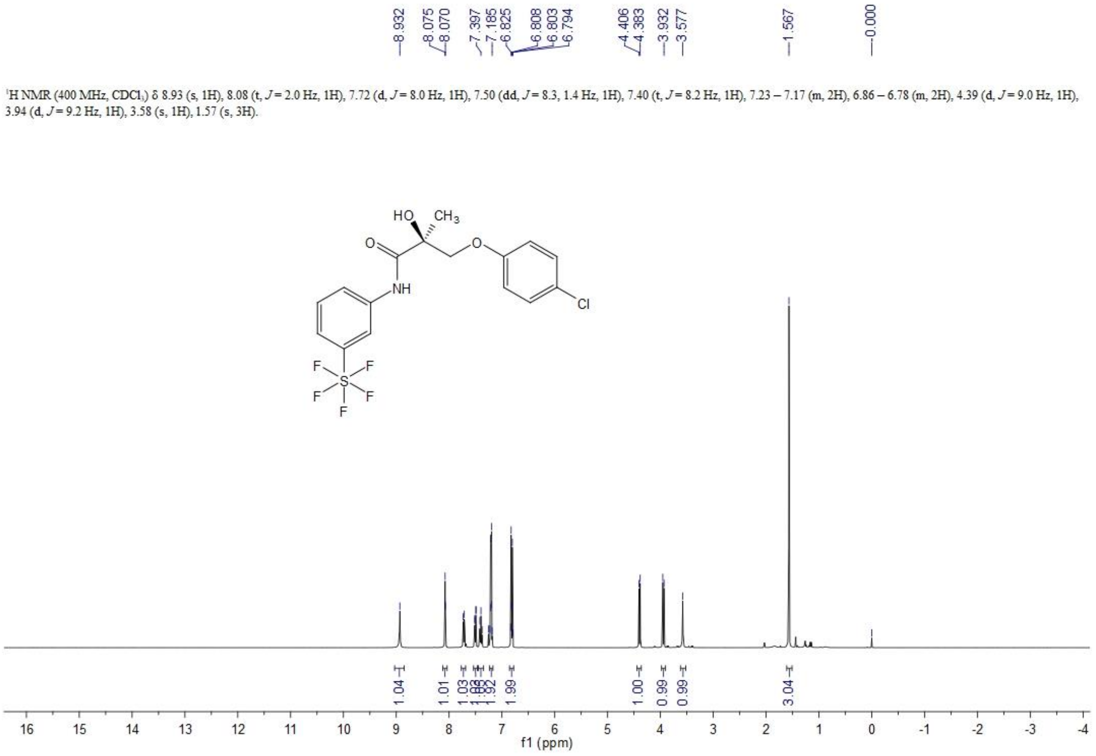

^{13}C NMR (101 MHz, $CDCl_3$) δ 172.30, 156.50, 154.25, 137.63, 129.50, 129.24, 126.84, 122.56, 121.90, 117.39, 116.11, 75.59, 72.91, 22.95.

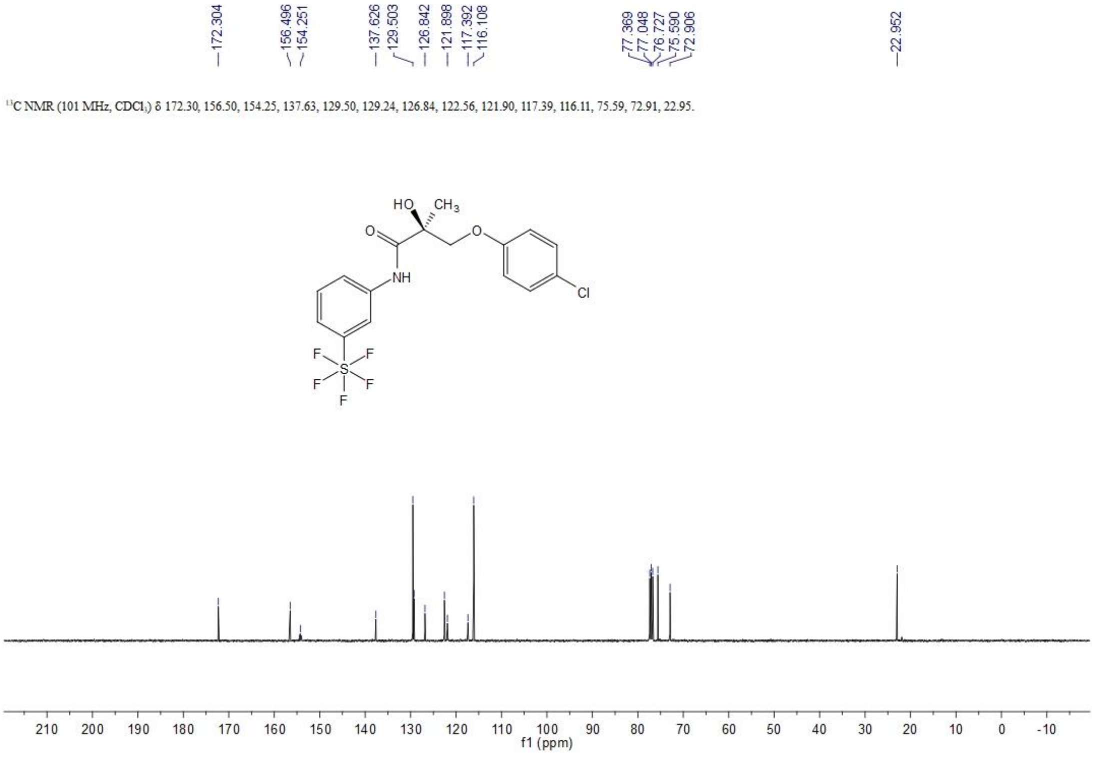

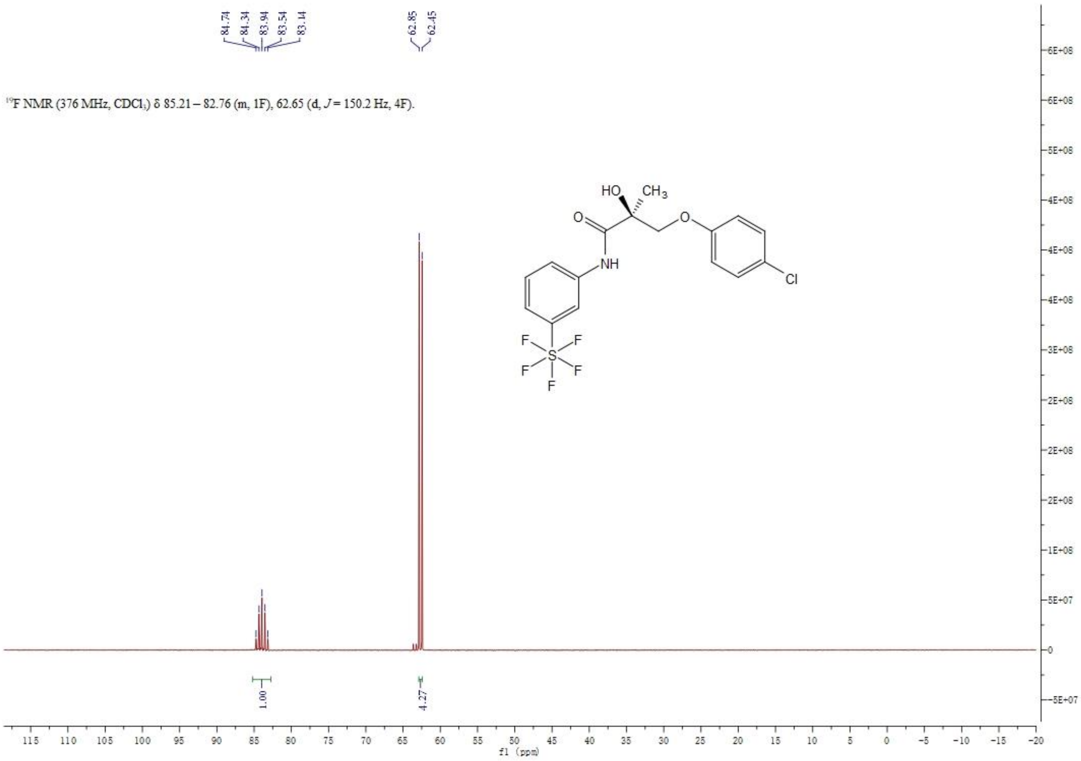

Elemental Composition Report **Page 1**

Single Mass Analysis
Tolerance = 5.0 mDa / DBE: min = -1.5, max = 50.0
Element prediction: Off

Monoisotopic Mass, Even Electron Ions
3 formula(e) evaluated with 1 results within limits (up to 50 closest results for each mass)
Elements Used:
C: 16-16 H: 16-16 N: 1-1 O: 3-3 F: 5-8 S: 1-1 Cl: 1-1
JJY-A00188-090-1 27 (0.554)
1: TOF MS ES+

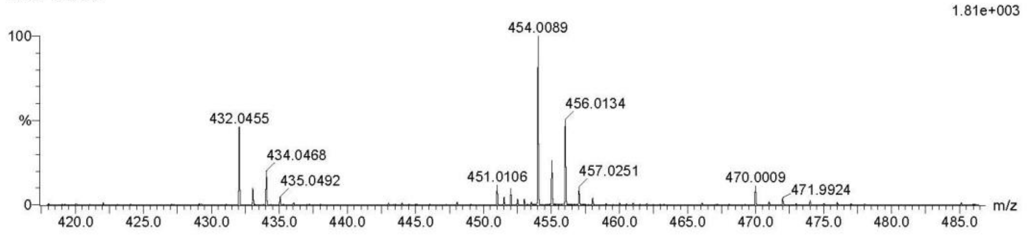

Minimum:				-1.5	
Maximum:		5.0	10.0	50.0	
Mass	Calc. Mass	mDa	PPM	DBE	Formula
432.0455	432.0460	-0.5	-1.2	6.5	C16 H16 N O3 F5 S Cl

Data File D:\AGILENT DATA\DATA\JJY\JJY20160203 2016-03-18 14-58-22\JJY0000002.D
Sample Name: JJY-A00188-090

===

Acq. Operator	: JJY	Seq. Line :	2
Acq. Instrument	: Instrument 1	Location :	Vial 82
Injection Date	: 3/18/2016 3:21:11 PM	Inj :	1
		Inj Volume :	10.0 µl

Acq. Method : D:\AGILENT DATA\DATA\JJY\JJY20160203 2016-03-18 14-58-22\JJY-0.1TFA-CH3CN-15MIN.M
Last changed : 3/17/2016 12:29:03 PM by JJY
Analysis Method : D:\AGILENT DATA\METHOD\JJY-0.1TFA-CH3CN-15MIN.M
Last changed : 3/17/2016 12:29:03 PM by JJY
Additional Info : Peak(s) manually integrated

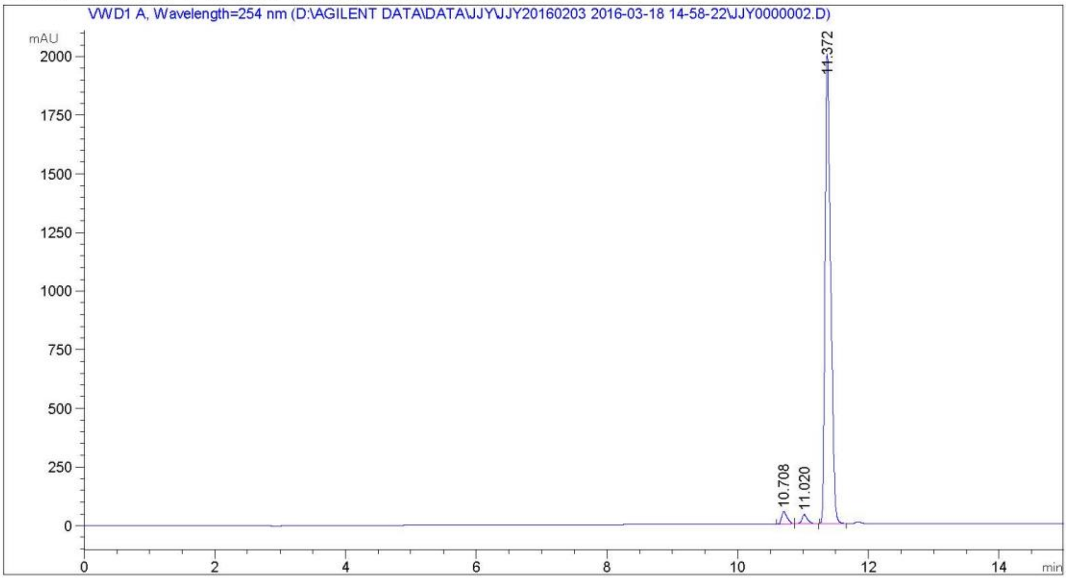

===

Area Percent Report

===

Sorted By : Signal
Multiplier : 1.0000
Dilution : 1.0000
Use Multiplier & Dilution Factor with ISTDs

Signal 1: VWD1 A, Wavelength=254 nm

Peak #	RetTime [min]	Type	Width [min]	Area mAU *s	Height [mAU]	Area %
1	10.708	BV	0.0870	312.26282	52.38766	2.5299
2	11.020	VB	0.0878	238.30028	39.50487	1.9307
3	11.372	BB	0.0862	1.17921e4	2002.54834	95.5394
Totals :				1.23427e4	2094.44088	

^{1}H, ^{13}C, ^{19}F NMR, HPLC and HRMS spectra of *compound* **12d**

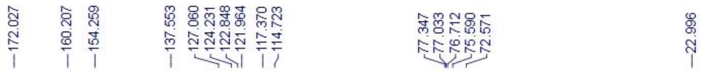

^{13}C NMR (101 MHz, $CDCl_3$) δ 172.03, 160.21, 154.26, 137.55, 129.28, 127.10, 127.06, 125.54, 124.23, 123.90, 122.85, 122.51, 121.96, 117.37, 114.72,75.59, 72.57, 23.00.

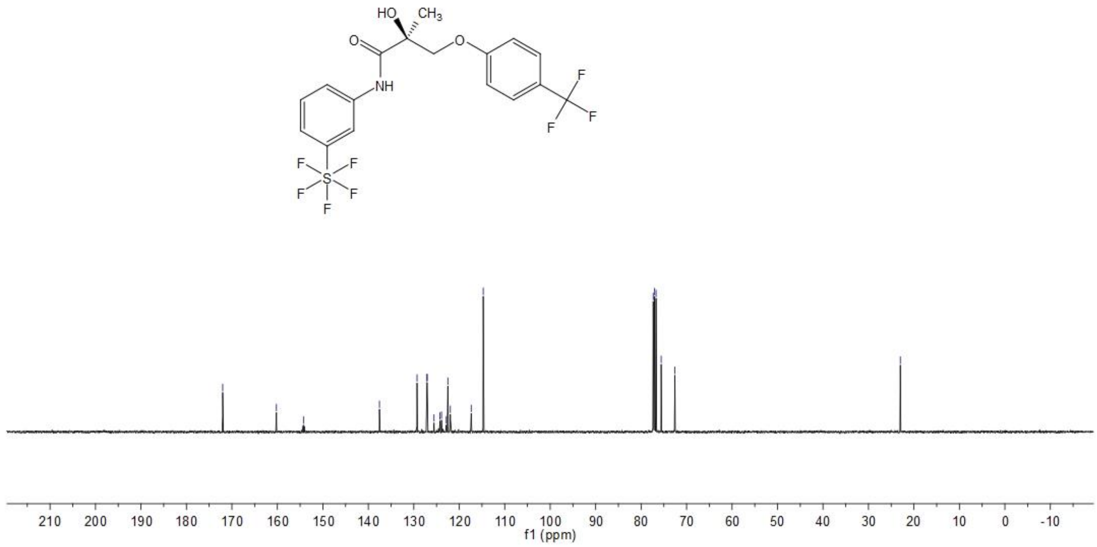

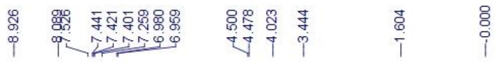

^{1}H NMR (400 MHz, $CDCl_3$) δ 8.93 (s, 1H), 8.09 (s, 1H), 7.74 (d, J = 8.0 Hz, 1H), 7.53 (t, J = 6.8 Hz, 3H), 7.42 (t, J = 8.2 Hz, 1H), 6.97 (d, J = 8.4 Hz, 2H), 4.49 (d, J = 9.0 Hz, 1H), 4.03 (d, J = 9.0 Hz, 1H), 3.44 (s, 1H), 1.60 (s, 3H).

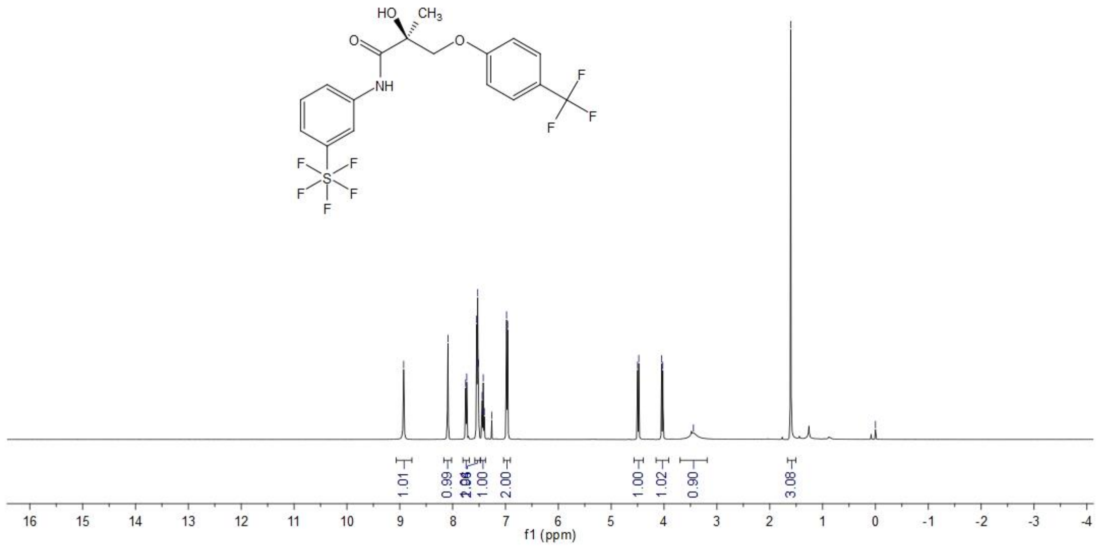

^{19}F NMR (376 MHz, $CDCl_3$) δ 84.86 – 82.96 (m, 1F), 62.64 (d, *J* = 150.1 Hz, 4F), -61.69 (s, 3F).

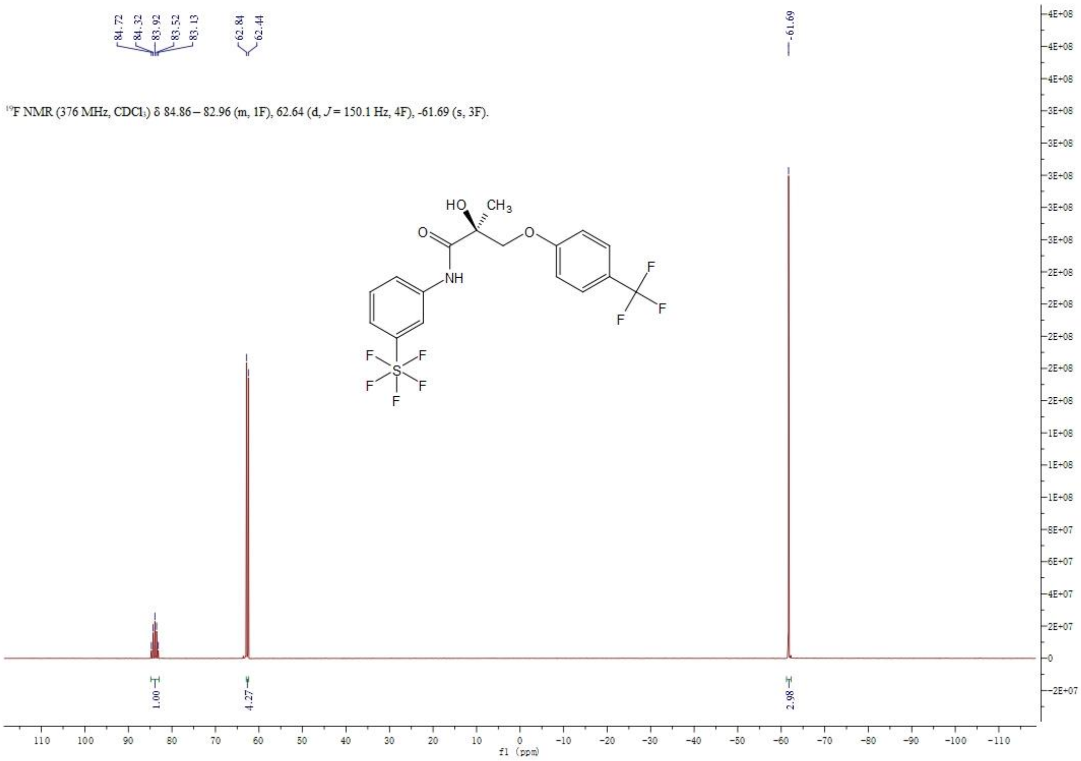

Elemental Composition Report

Page 1

Single Mass Analysis

Tolerance = 100.0 PPM / DBE: min = -1.5, max = 50.0
Element prediction: Off

Monoisotopic Mass, Even Electron Ions
1 formula(e) evaluated with 1 results within limits (up to 50 closest results for each mass)
Elements Used:
C: 17-17 H: 15-15 N: 1-1 O: 3-3 F: 8-8 Na: 0-1 S: 1-1
JJY-A00188-095 61 (1.211)
1: TOF MS ES+

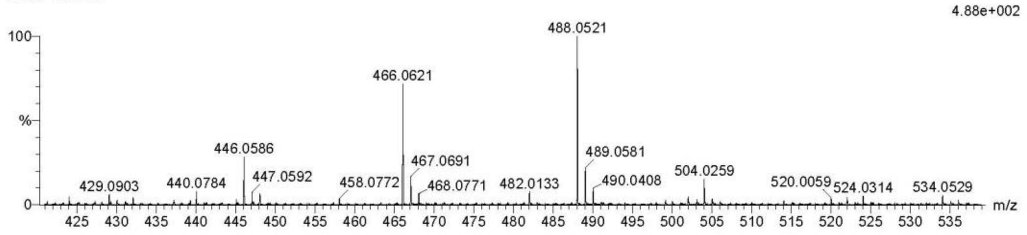

Minimum:				-1.5	
Maximum:		5.0	100.0	50.0	
Mass	Calc. Mass	mDa	PPM	DBE	Formula
488.0521	488.0543	-2.2	-4.5	6.5	C17 H15 N O3 F8 Na S

数据文件: C:\CHEM32\1\DATA\JJY\LW000036.D
样品名称: JJY-A00188-095

===

操作者 : spx
仪器 : 仪器 1 位置 : 样品瓶 1
进样日期 : 2019/11/14 15:45:55
进样量 : 没有进样
采集方法 : C:\CHEM32\1\METHODS\JJY-15MIN.M
最后修改 : 2019/11/14 15:42:28 : spx
(调用后修改)
分析方法 : C:\CHEM32\1\METHODS\JJY-15MIN.M
最后修改 : 2019/11/4 16:29:42 : CYT
附加信息: 峰已手动积分

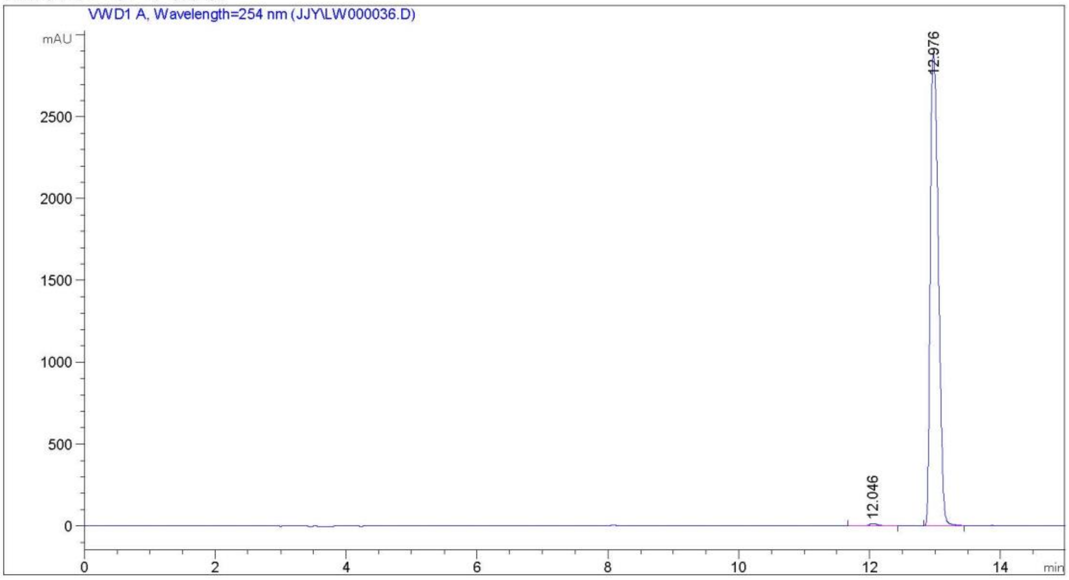

===

面积百分比报告

===

排序 : 信号
乘积因子: : 1.0000
稀释因子: : 1.0000
内标使用乘积因子和稀释因子

信号 1: VWD1 A, Wavelength=254 nm

峰 #	保留时间 [min]	类型	峰宽 [min]	峰面积 [mAU*s]	峰高 [mAU]	峰面积 %
1	12.046	BB	0.1280	127.06351	15.44123	0.5078
2	12.976	VV	0.1416	2.48932e4	2881.67456	99.4922

总量 : 2.50203e4 2897.11579

===

^{1}H, ^{13}C, ^{19}F NMR, HPLC and HRMS spectra of *compound* **12e**

^{1}H NMR (400 MHz, DMSO) δ 10.19 (s, 1H), 9.76 (s, 1H), 8.53 (s, 1H), 8.06 (d, J = 7.5 Hz, 1H), 7.57 (p, J = 8.3 Hz, 2H), 7.45 (d, J = 8.9 Hz, 2H), 6.86 (d, J = 8.9 Hz, 2H), 6.13 (s, 1H), 4.17 (d, J = 9.5 Hz, 1H), 3.93 (d, J = 9.5 Hz, 1H), 2.00 (s, 3H), 1.43 (s, 3H).

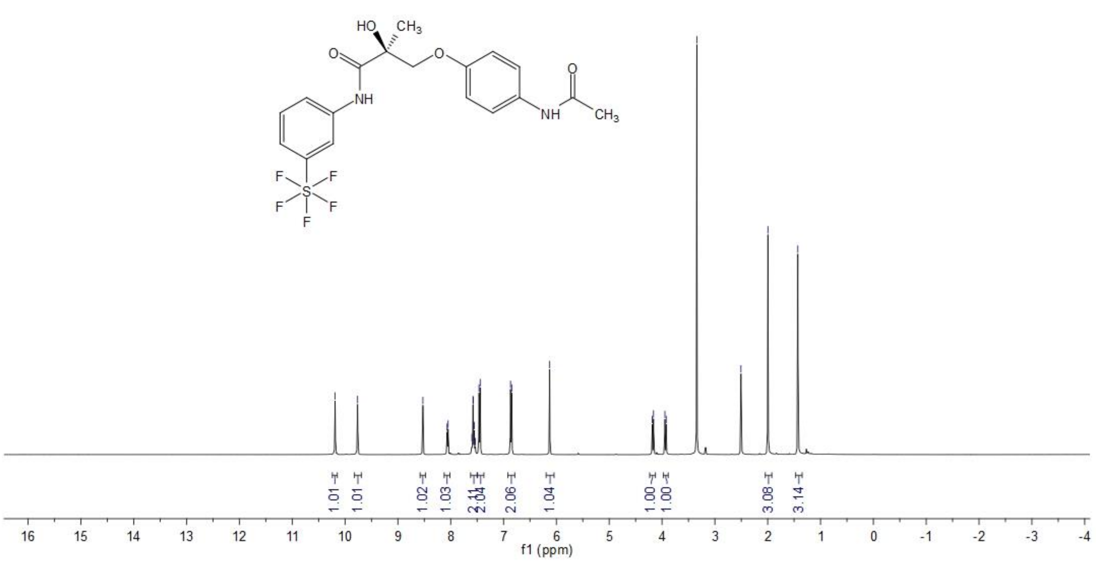

^{13}C NMR (101 MHz, DMSO) δ 173.81, 167.68, 154.23, 152.87, 139.30, 132.74, 129.49, 123.32, 120.33, 116.73, 114.55, 74.67, 73.73, 23.76, 23.04.

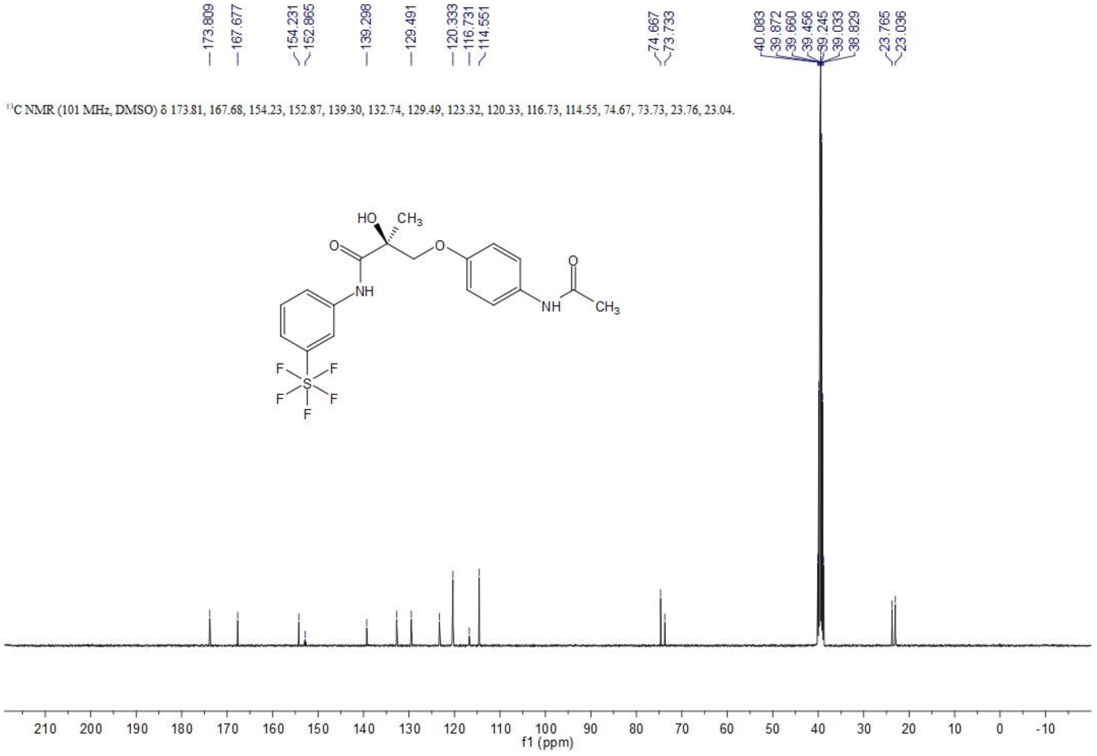

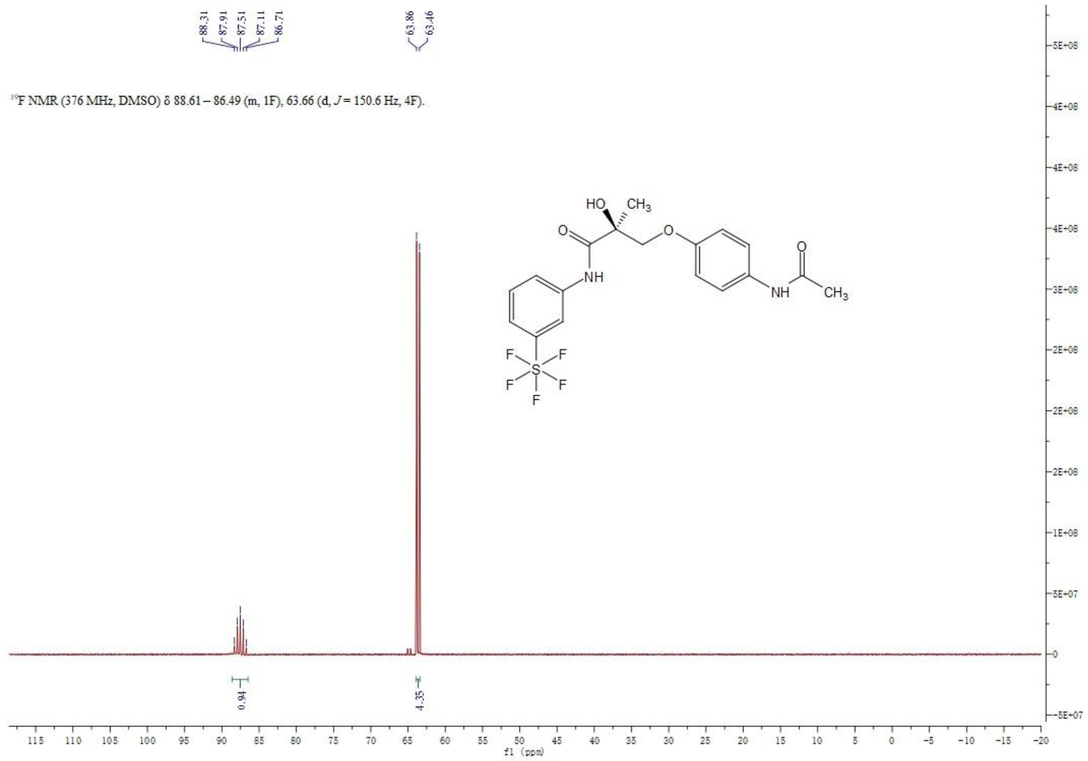

Elemental Composition Report **Page 1**

Single Mass Analysis
Tolerance = 500.0 PPM / DBE: min = -1.5, max = 50.0
Element prediction: Off

Monoisotopic Mass, Even Electron Ions
1 formula(e) evaluated with 1 results within limits (up to 50 closest results for each mass)
Elements Used:
C: 18-18 H: 20-20 N: 2-2 O: 4-4 F: 5-5 S: 1-1
JJY-A00188-086_2 32 (0.639)
1: TOF MS ES+

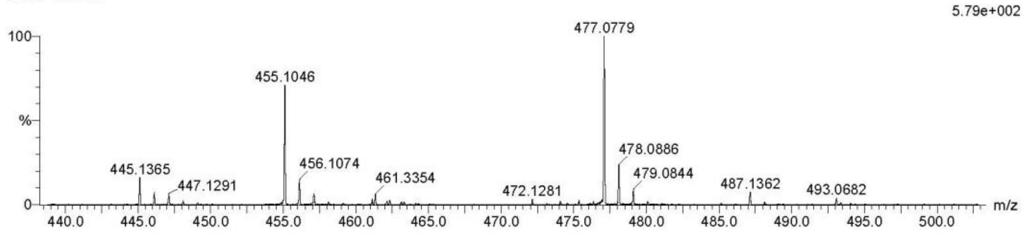

Minimum:				-1.5	
Maximum:		5.0	500.0	50.0	
Mass	Calc. Mass	mDa	PPM	DBE	Formula
455.1046	455.1064	-1.8	-4.0	7.5	C18 H20 N2 O4 F5 S

Data File D:\AGILENT DATA\DATA\JJY\JJY20160203 2016-03-15 13-28-32\JJY0000003.D
Sample Name: JJY-A00188-086

===

Acq. Operator : JJY   Seq. Line : 3
Acq. Instrument : Instrument 1   Location : Vial 83
Injection Date : 3/15/2016 2:13:00 PM   Inj : 1
                                                      Inj Volume : 10.0 µl
Different Inj Volume from Sequence !   Actual Inj Volume : 5.0 µl
Acq. Method : D:\AGILENT DATA\DATA\JJY\JJY20160203 2016-03-15 13-28-32\JJY-0.1TFA-CH3CN-15MIN.M
Last changed : 3/14/2016 12:04:47 PM by JJY
Analysis Method : D:\AGILENT DATA\METHOD\JJY-0.1TFA-CH3CN-15MIN.M
Last changed : 3/14/2016 12:04:47 PM by JJY
Additional Info : Peak(s) manually integrated

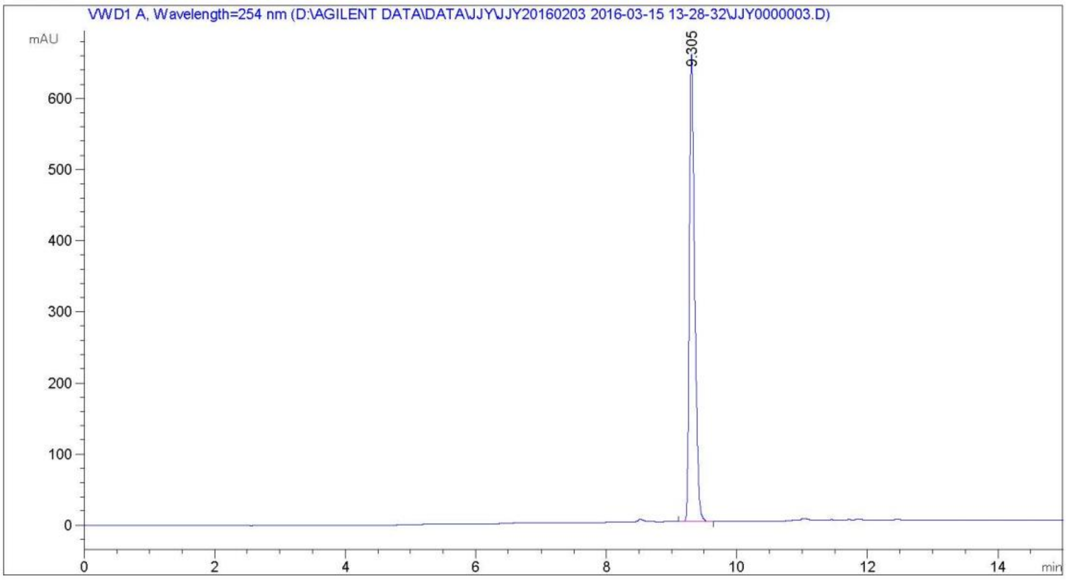

===

Area Percent Report

===

Sorted By : Signal
Multiplier : 1.0000
Dilution : 1.0000
Use Multiplier & Dilution Factor with ISTDs

Signal 1: VWD1 A, Wavelength=254 nm

Peak #	RetTime [min]	Type	Width [min]	Area mAU *s	Height [mAU]	Area %
1	9.305	BV	0.0848	3713.10205	657.75159	100.0000

Totals : 3713.10205 657.75159

===

^{1}H, ^{13}C, ^{19}F NMR, HPLC and HRMS spectra of *compound* **12f**·

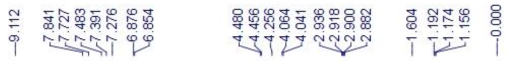

^{1}H NMR (400 MHz, $CDCl_3$) δ 9.11 (s, 1H), 8.12 (s, 1H), 7.85 (d, *J* = 8.7 Hz, 2H), 7.74 (d, *J* = 8.0 Hz, 1H), 7.49 (d, *J* = 8.3 Hz, 1H), 7.39 (t, *J* = 8.2 Hz, 1H), 6.86 (d, *J* = 8.5 Hz, 2H), 4.47 (d, *J* = 9.3 Hz, 1H), 4.26 (s, 1H), 4.05 (d, *J* = 9.2 Hz, 1H), 2.91 (q, *J* = 7.2 Hz, 2H), 1.60 (s, 3H), 1.17 (t, *J* = 7.3 Hz, 3H).

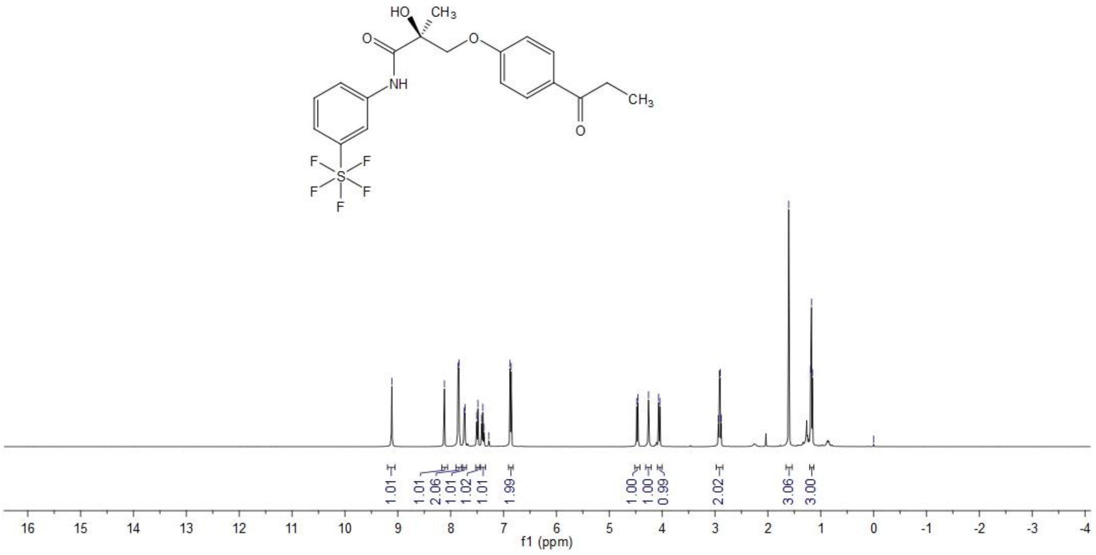

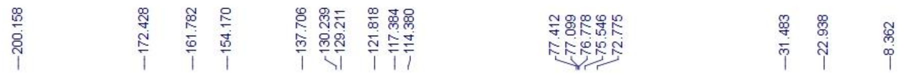

^{13}C NMR (101 MHz, $CDCl_3$) δ 200.16, 172.43, 161.78, 154.17, 137.71, 130.52, 130.24, 129.21, 122.60, 121.82, 117.38, 114.38, 75.55, 72.78, 31.48, 22.94, 8.36.

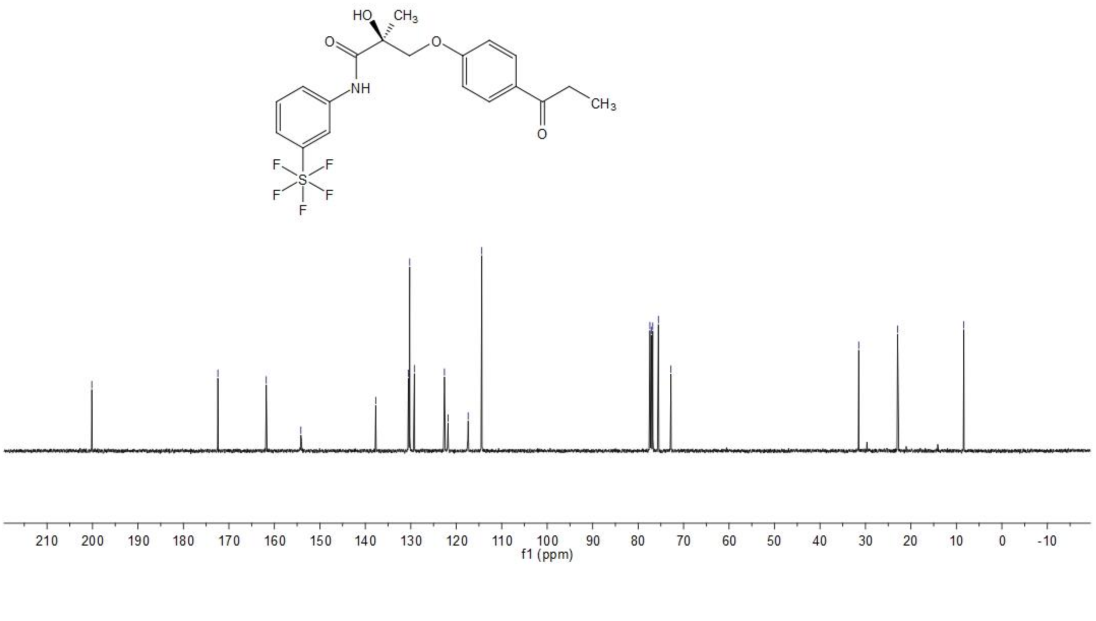

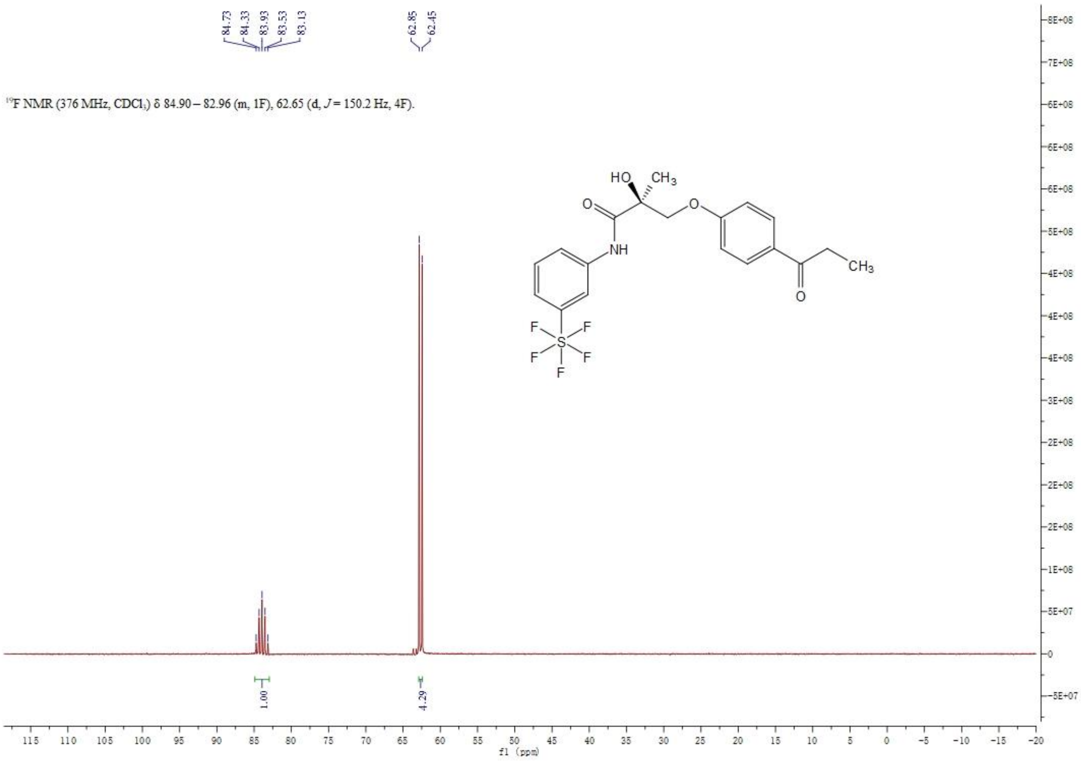

Elemental Composition Report

Page 1

Single Mass Analysis

Tolerance = 100.0 PPM / DBE: min = -1.5, max = 50.0
Element prediction: Off

Monoisotopic Mass, Even Electron Ions
1 formula(e) evaluated with 1 results within limits (up to 50 closest results for each mass)
Elements Used:
C: 19-19 H: 20-20 N: 1-1 O: 4-4 F: 5-5 S: 1-1 Na: 0-1

JJY-A00188-094 80 (1.573)
1: TOF MS ES+

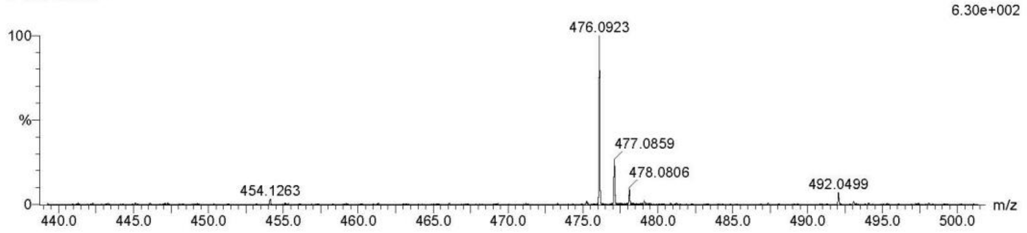

Mass	Calc. Mass	mDa	PPM	DBE	Formula
Minimum:				-1.5	
Maximum:		5.0	100.0	50.0	
476.0923	476.0931	-0.8	-1.7	7.5	C19 H20 N O4 F5 S Na

数据文件: C:\CHEM32\1\DATA\JJY\LW000038.D
样品名称: JJY-A00188-094

===

操作者 : spx
仪器 : 仪器 1 位置 : 样品瓶 1
进样日期 : 2019/11/14 16:34:18
进样量 : 没有进样
采集方法 : C:\CHEM32\1\METHODS\JJY-15MIN.M
最后修改 : 2019/11/14 16:33:32 : spx
(调用后修改)
分析方法 : C:\CHEM32\1\METHODS\JJY-15MIN.M
最后修改 : 2019/11/4 16:29:42 : CYT
附加信息: 峰已手动积分

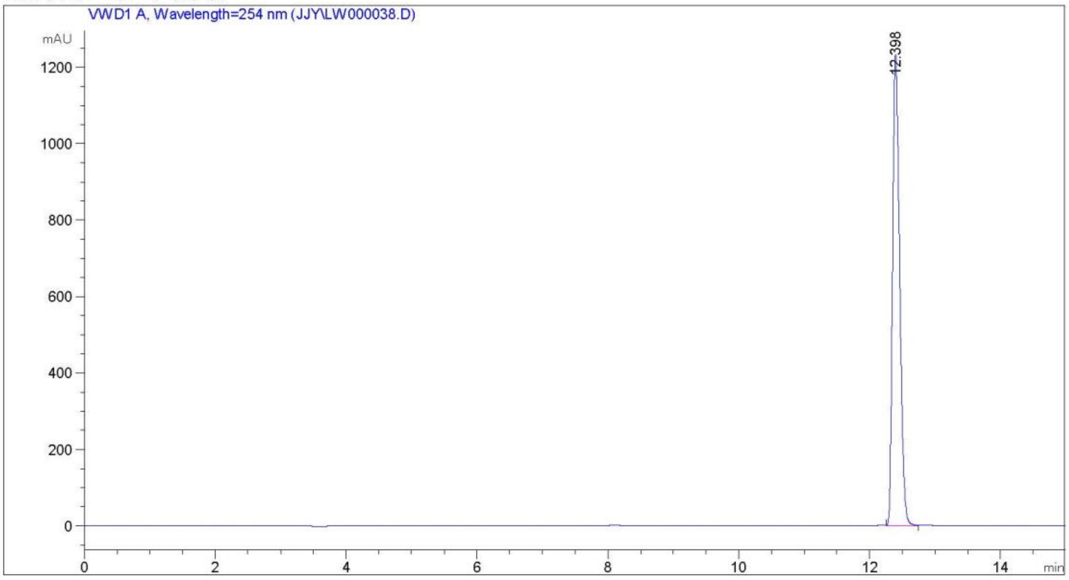

===

面积百分比报告

===

排序 : 信号
乘积因子: : 1.0000
稀释因子: : 1.0000
内标使用乘积因子和稀释因子

信号 1: VWD1 A, Wavelength=254 nm

峰 #	保留时间 [min]	类型	峰宽 [min]	峰面积 [mAU*s]	峰高 [mAU]	峰面积 %
1	12.398	VV	0.1164	9237.38672	1234.50305	100.0000

总量 : 9237.38672 1234.50305

===

*** 报告结束 ***

仪器 1 2019/11/14 18:06:55 spx 页 1/1

^{1}H, ^{13}C, ^{19}F NMR, HPLC and HRMS spectra of *compound* **12g**

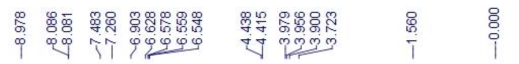

^{1}H NMR (400 MHz, $CDCl_3$) δ 8.98 (s, 1H), 8.09 (t, *J* = 2.0 Hz, 1H), 7.71 (d, *J* = 8.2 Hz, 1H), 7.49 (dd, *J* = 8.3, 1.4 Hz, 1H), 7.39 (t, *J* = 8.2 Hz, 1H), 6.93 (t, *J* = 9.2 Hz, 1H), 6.64 (dd, *J* = 12.6, 2.9 Hz, 1H), 6.56 (ddd, *J* = 9.0, 2.9, 1.5 Hz, 1H), 4.43 (d, *J* = 9.3 Hz, 1H), 3.97 (d, *J* = 9.2 Hz, 1H), 3.90 (s, 1H), 3.72 (s, 3H), 1.56 (s, 3H).

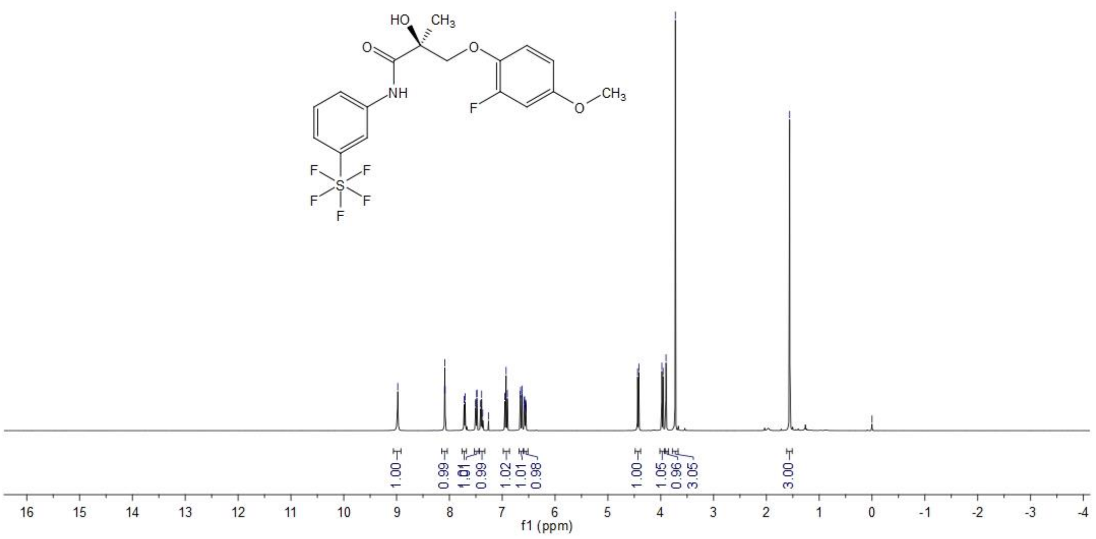

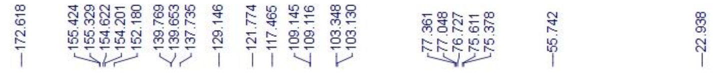

^{13}C NMR (101 MHz, $CDCl_3$) δ 172.62, 155.42, 155.33, 154.62, 154.20, 152.18, 139.77, 139.65, 137.74, 129.15, 122.63, 121.77, 118.00, 117.98, 117.46, 109.15, 109.12, 103.35, 103.13, 75.61, 75.38, 55.74, 22.94.

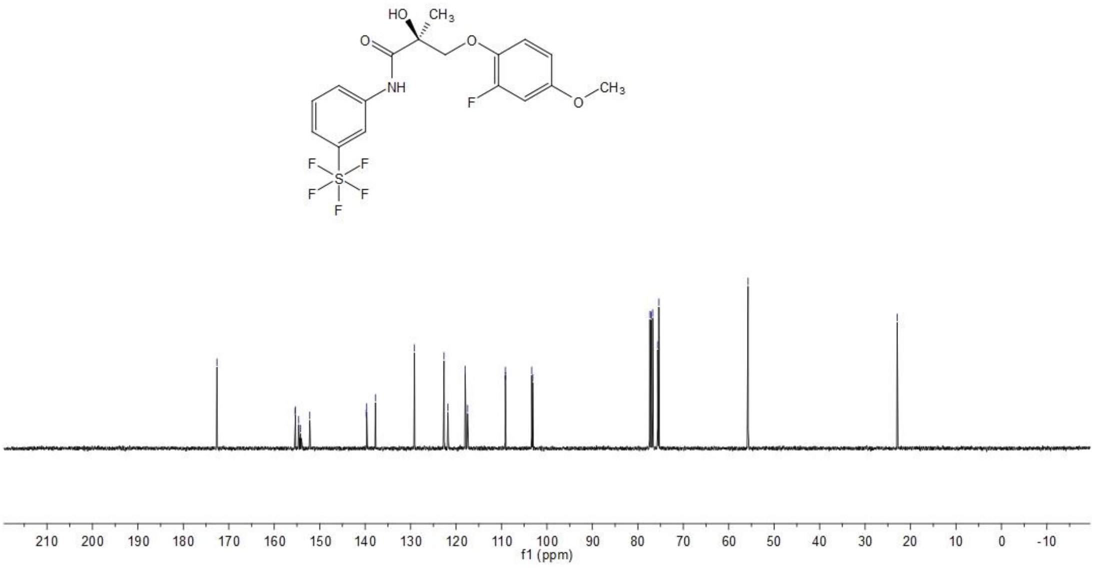

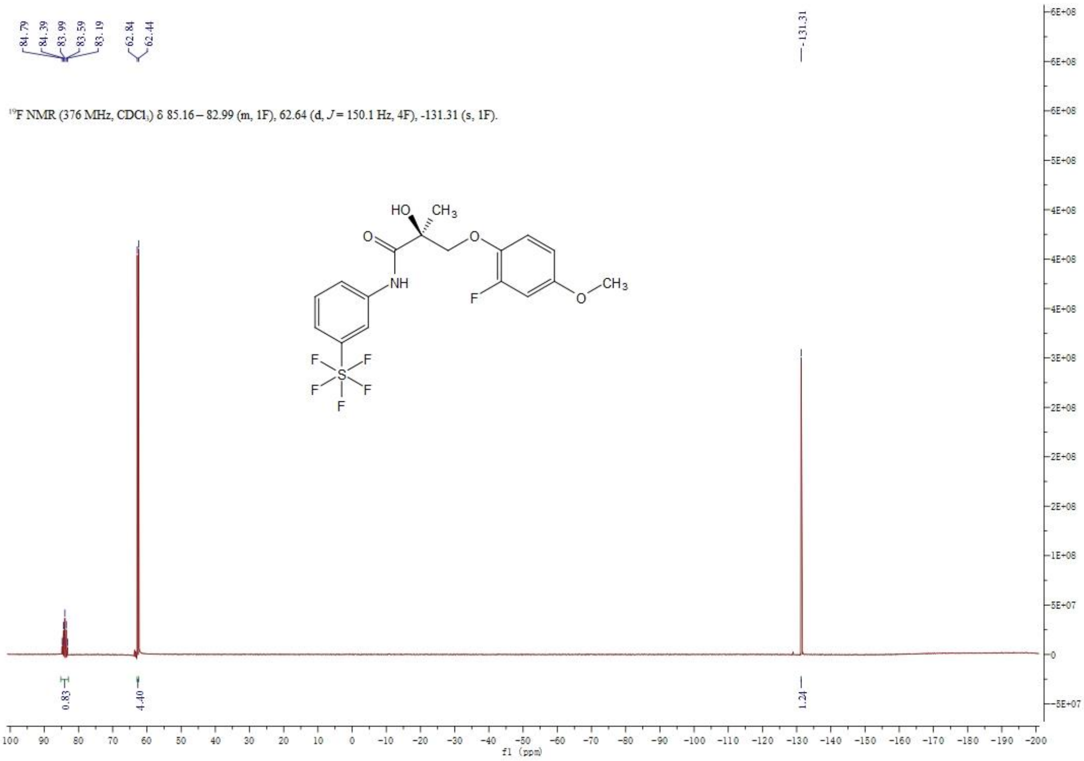

Elemental Composition Report

Page 1

Single Mass Analysis

Tolerance = 500.0 PPM / DBE: min = -1.5, max = 50.0
Element prediction: Off

Monoisotopic Mass, Even Electron Ions
1 formula(e) evaluated with 1 results within limits (up to 50 closest results for each mass)
Elements Used:
C: 17-17 H: 17-18 N: 1-1 O: 4-4 S: 1-1 F: 6-6 Na: 0-1

JJY-A00188-092-1 50 (1.004)
1: TOF MS ES+

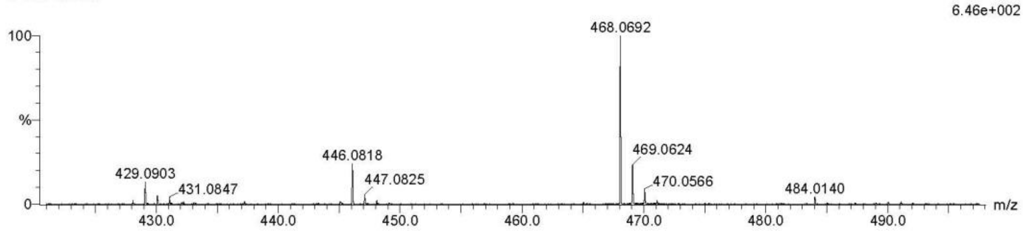

Minimum:				-1.5	
Maximum:		5.0	500.0	50.0	
Mass	Calc. Mass	mDa	PPM	DBE	Formula
468.0692	468.0680	1.2	2.6	6.5	C17 H17 N O4 S F6 Na

数据文件: C:\CHEM32\1\DATA\JJY\LW000037.D
样品名称: JJY-A00188-092

===

操作者 : spx
仪器 : 仪器 1 位置 : 样品瓶 1
进样日期 : 2019/11/14 16:13:34
进样量 : 没有进样
采集方法 : C:\CHEM32\1\METHODS\JJY-15MIN.M
最后修改 : 2019/11/14 16:05:53 : spx
(调用后修改)
分析方法 : C:\CHEM32\1\METHODS\JJY-15MIN.M
最后修改 : 2019/11/4 16:29:42 : CYT
附加信息: 峰已手动积分

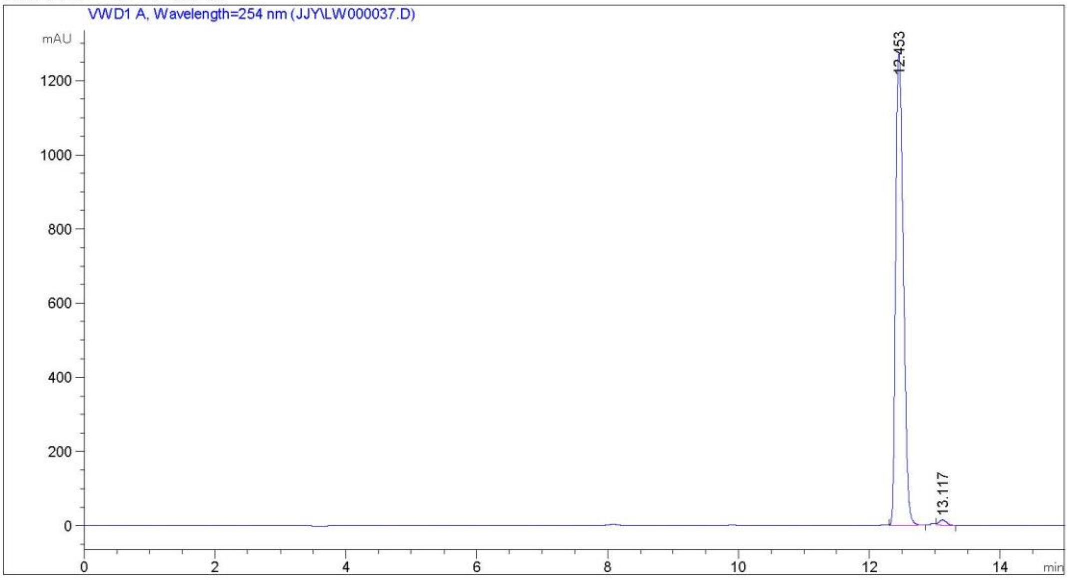

===

面积百分比报告

===

排序 : 信号
乘积因子: : 1.0000
稀释因子: : 1.0000
内标使用乘积因子和稀释因子

信号 1: VWD1 A, Wavelength=254 nm

峰 #	保留时间 [min]	类型	峰宽 [min]	峰面积 [mAU*s]	峰高 [mAU]	峰面积 %
1	12.453	VB	0.1320	1.04140e4	1270.64783	98.8690
2	13.117	VB	0.1248	119.13281	14.75413	1.1310

总量 : 1.05331e4 1285.40196

===

仪器 1 2019/11/14 18:07:30 spx 页 1/1

^{1}H, ^{13}C, ^{19}F NMR, HPLC and HRMS spectra of *compound* **13a**

^{1}H NMR (400 MHz, $CDCl_3$) δ 9.20 (s, 1H), 8.27 (s, 1H), 7.96 (d, J = 8.4 Hz, 1H), 7.78 (d, J = 8.5 Hz, 1H), 7.57 (d, J = 8.0 Hz, 2H), 6.98 (d, J = 8.2 Hz, 2H), 4.50 (d, J = 9.2 Hz, 1H), 4.07 (d, J = 9.0 Hz, 1H), 3.53 (s, 1H), 1.63 (s, 3H).

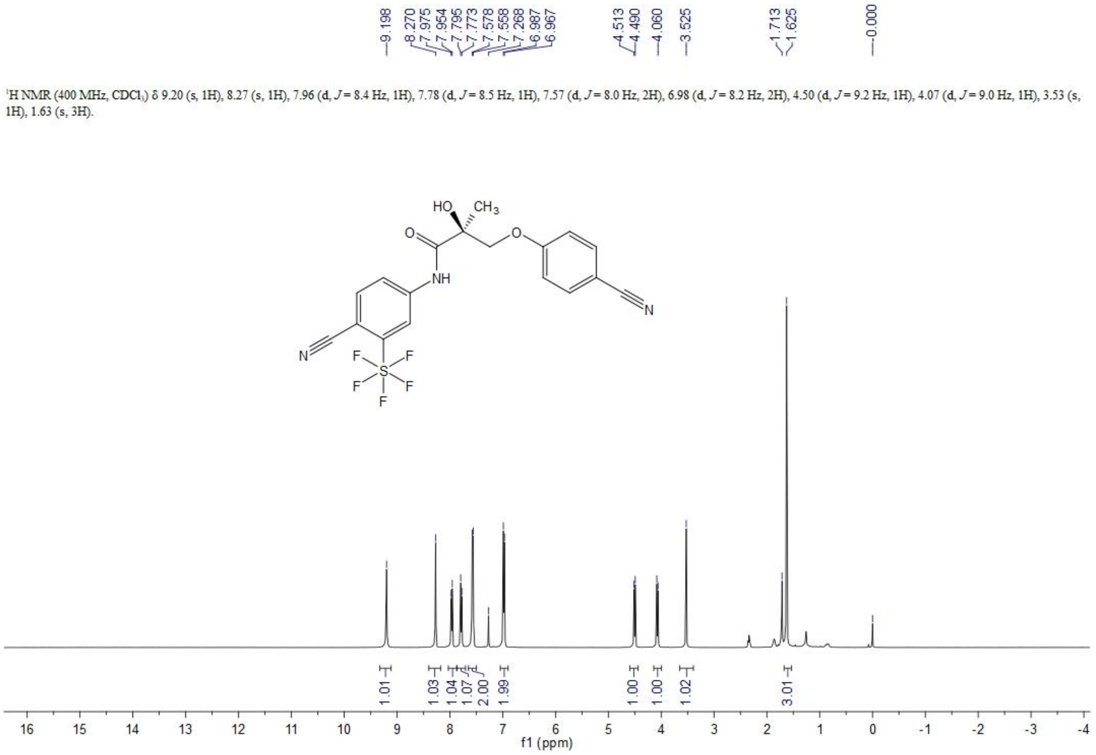

^{13}C NMR (101 MHz, $CDCl_3$) δ 172.36, 161.02, 155.55, 141.42, 136.05, 134.13, 121.61, 119.10, 118.76, 116.13, 115.46, 105.18, 104.46, 75.79, 72.60, 22.99.

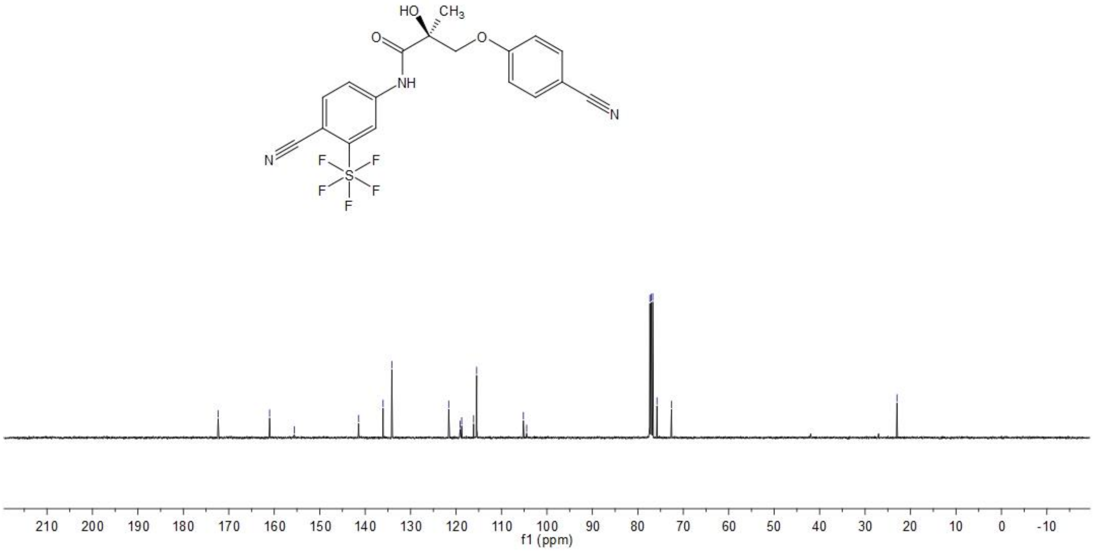

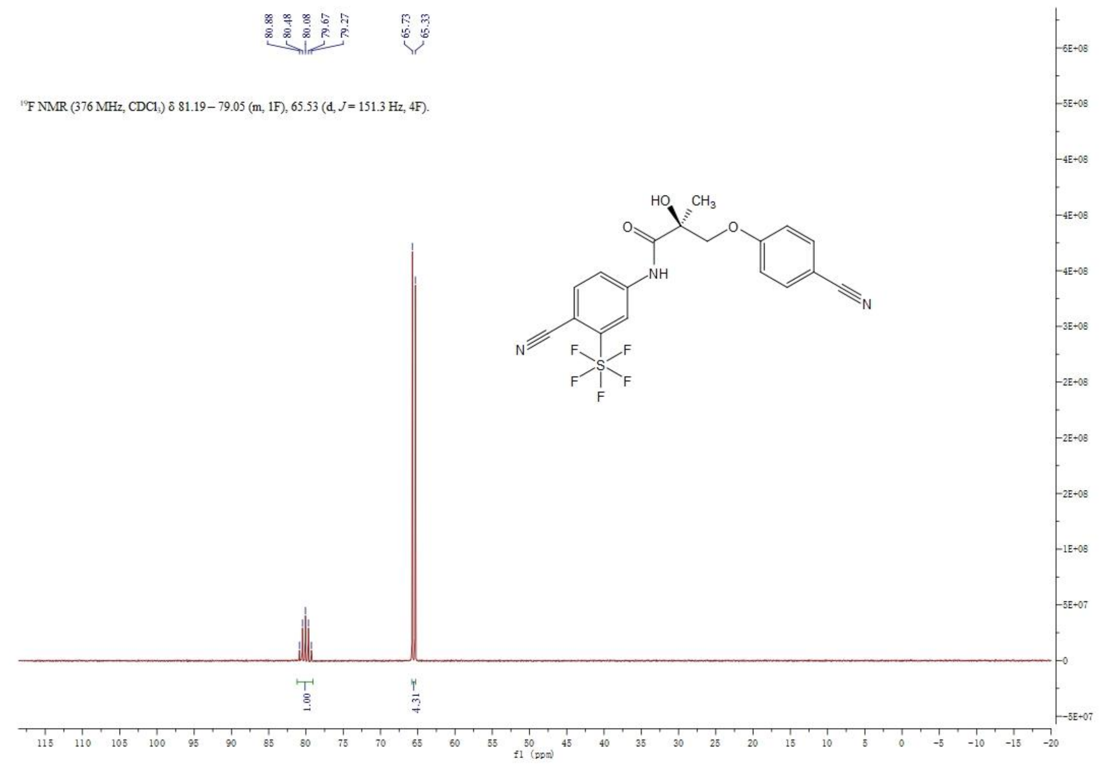

Elemental Composition Report **Page 1**

Single Mass Analysis
Tolerance = 100.0 PPM / DBE: min = -1.5, max = 50.0
Element prediction: Off

Monoisotopic Mass, Even Electron Ions
1 formula(e) evaluated with 1 results within limits (up to 50 closest results for each mass)
Elements Used:
C: 18-18 H: 14-14 N: 3-3 O: 3-3 F: 5-5 Na: 0-1 S: 1-1
JJY-A00188-044 32 (0.639)
1: TOF MS ES+

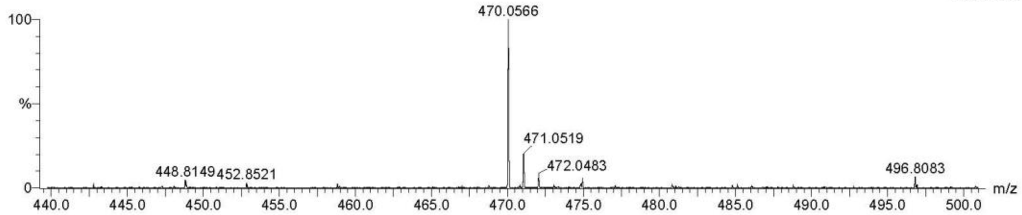

Minimum:				-1.5	
Maximum:		5.0	100.0	50.0	
Mass	Calc. Mass	mDa	PPM	DBE	Formula
470.0566	470.0574	-0.8	-1.7	10.5	C18 H14 N3 O3 F5 Na S

Data File D:\AGILENT DATA\DATA\HYS\JJY20160126 2016-10-26 14-53-07\031-0401.D
Sample Name: JJY-A00188-044

===

Acq. Operator	: HYS	Seq. Line :	4
Acq. Instrument	: Instrument 1	Location :	Vial 31
Injection Date	: 10/26/2016 3:58:13 PM	Inj :	1
		Inj Volume :	20.0 µl

Acq. Method : D:\AGILENT DATA\DATA\HYS\JJY20160126 2016-10-26 14-53-07\JJY-0.1TFA-CH3CN-15MIN.M
Last changed : 10/26/2016 3:48:25 PM by HYS
(modified after loading)
Analysis Method : D:\AGILENT DATA\METHOD\JJY-0.1TFA-CH3CN-15MIN-1.M
Last changed : 10/26/2016 9:54:22 AM by WS
Additional Info : Peak(s) manually integrated

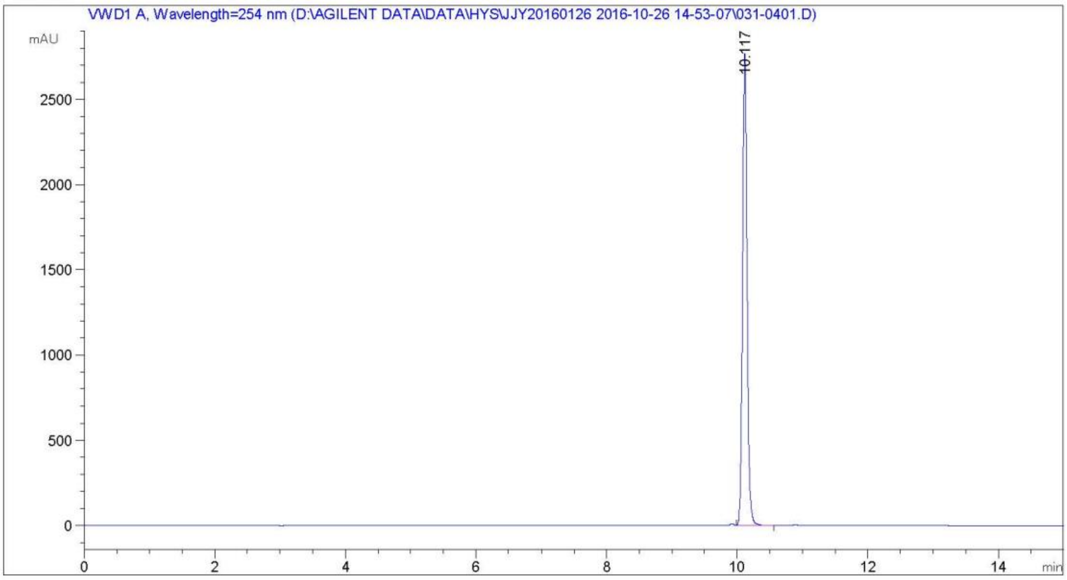

===

Area Percent Report

===

Sorted By : Signal
Multiplier : 1.0000
Dilution : 1.0000
Use Multiplier & Dilution Factor with ISTDs

Signal 1: VWD1 A, Wavelength=254 nm

Peak #	RetTime [min]	Type	Width [min]	Area mAU *s	Height [mAU]	Area %
1	10.117	VV	0.0743	1.35201e4	2775.39331	100.0000

Totals : 1.35201e4 2775.39331

===

^{1}H, ^{13}C, ^{19}F NMR, HPLC and HRMS spectra of *compound* **13b**

^{1}H NMR (400 MHz, $CDCl_3$) δ 9.16 (s, 1H), 8.25 (s, 1H), 7.96 (d, J = 8.5 Hz, 1H), 7.79 (d, J = 8.4 Hz, 1H), 6.98 (t, J = 8.4 Hz, 2H), 6.86 (dd, J = 8.8, 4.1 Hz, 2H), 4.43 (d, J = 9.0 Hz, 1H), 3.95 (d, J = 9.0 Hz, 1H), 3.46 (s, 1H), 1.59 (s, 3H).

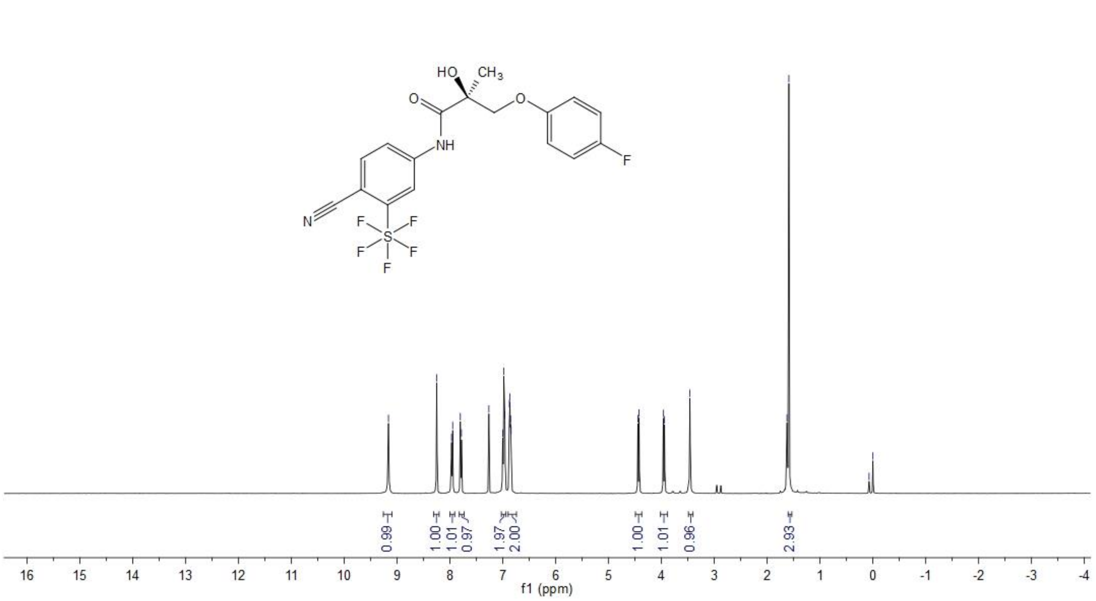

^{13}C NMR (101 MHz, $CDCl_3$) δ 172.76, 159.14, 156.76, 155.58, 153.75, 141.41, 136.07, 121.55, 119.04, 116.25, 116.15, 116.04, 116.02, 115.96, 104.48, 75.89, 73.05, 22.92.

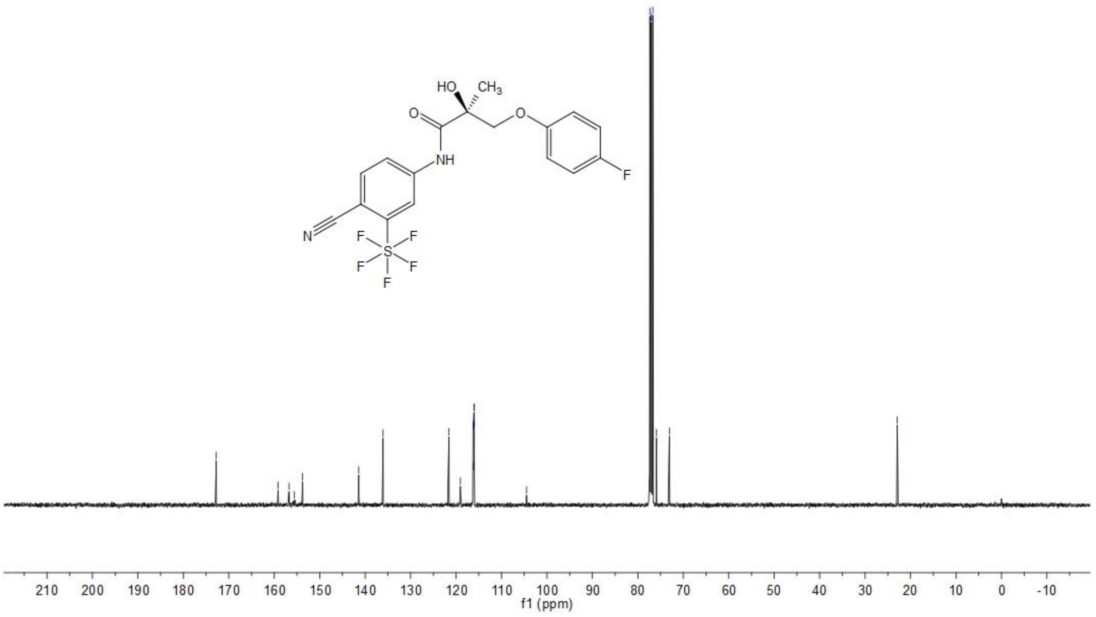

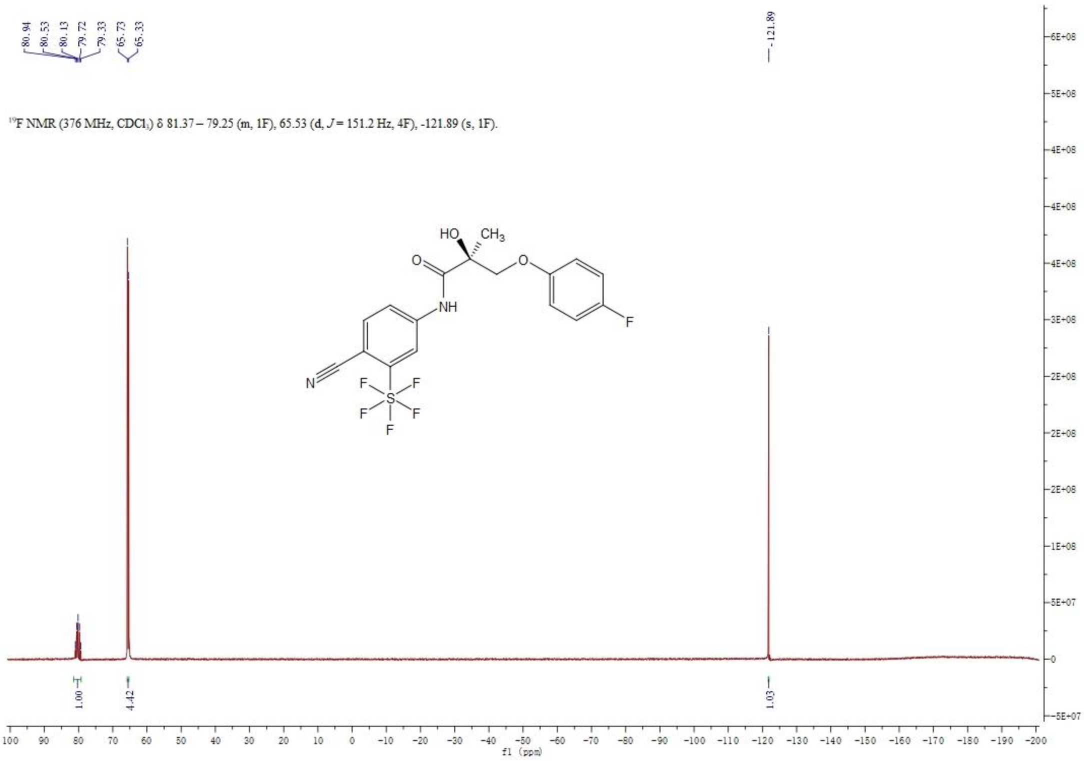

Elemental Composition Report **Page 1**

Single Mass Analysis
Tolerance = 100.0 PPM / DBE: min = -1.5, max = 50.0
Element prediction: Off

Monoisotopic Mass, Even Electron Ions
1 formula(e) evaluated with 1 results within limits (up to 50 closest results for each mass)
Elements Used:
C: 17-17 H: 15-15 N: 2-2 O: 3-3 F: 6-6 S: 1-1
JJY-A00188-105 25 (0.520)
1: TOF MS ES+

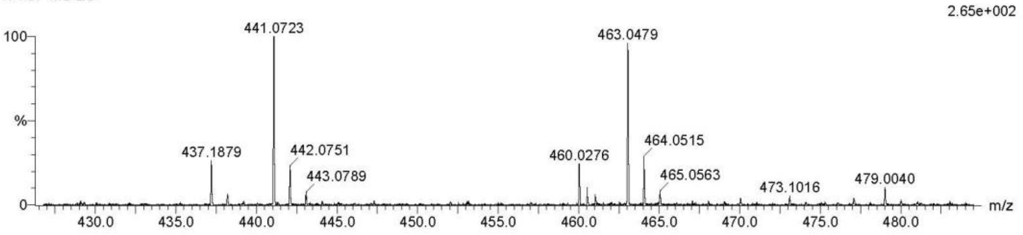

Minimum:				-1.5	
Maximum:		5.0	100.0	50.0	
Mass	Calc. Mass	mDa	PPM	DBE	Formula
441.0723	441.0708	1.5	3.4	8.5	C17 H15 N2 O3 F6 S

数据文件: C:\CHEM32\1\DATA\JJY\LW000027.D
样品名称: JJY-A00188-105

===

操作者	: spx		
仪器	: 仪器 1	位置	: 样品瓶 1
进样日期	: 2019/11/14 12:22:12		
		进样量	: 没有进样
采集方法	: C:\CHEM32\1\METHODS\JJY-15MIN.M		
最后修改	: 2019/11/14 12:20:42 : spx<br>(调用后修改)		
分析方法	: C:\CHEM32\1\METHODS\JJY-15MIN.M		
最后修改	: 2019/11/4 16:29:42 : CYT		

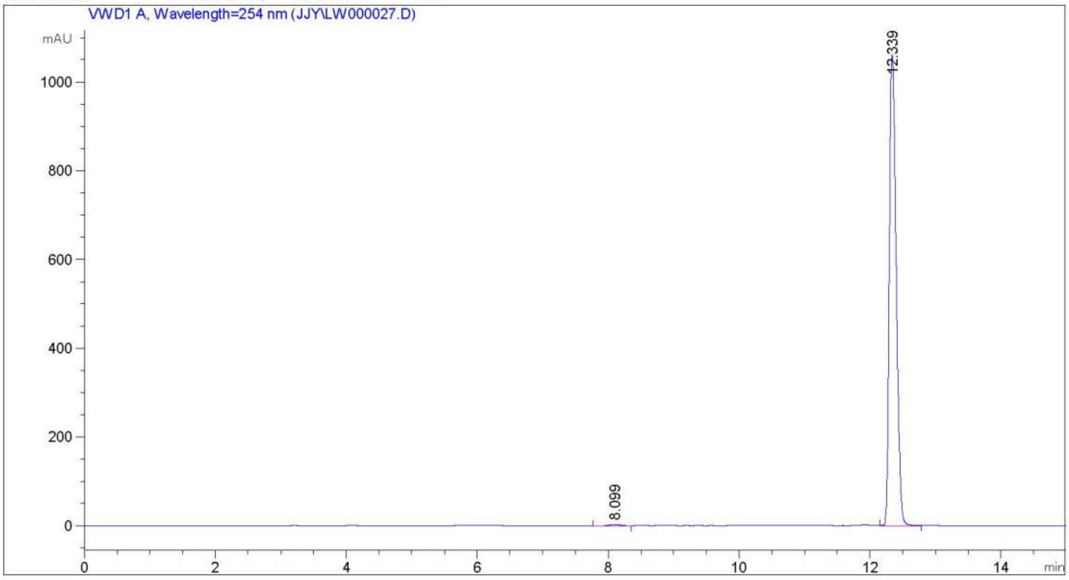

===

面积百分比报告

===

排序	:	信号
乘积因子:	:	1.0000
稀释因子:	:	1.0000

内标使用乘积因子和稀释因子

信号 1: VWD1 A, Wavelength=254 nm

峰 #	保留时间 [min]	类型	峰宽 [min]	峰面积 [mAU*s]	峰高 [mAU]	峰面积 %
1	8.099	BB	0.1456	24.87596	2.55602	0.3206
2	12.339	VV	0.1140	7735.18408	1063.18188	99.6794

总量 : 7760.06004 1065.73790

===

*** 报告结束 ***

^{1}H, ^{13}C, ^{19}F NMR, HPLC and HRMS spectra of *compound* **13c**

^{1}H NMR (400 MHz, $CDCl_3$) δ 9.14 (s, 1H), 8.24 (s, 1H), 7.95 (d, J = 8.5 Hz, 1H), 7.79 (d, J = 8.5 Hz, 1H), 7.25 (d, J = 9.0 Hz, 2H), 6.84 (d, J = 8.8 Hz, 2H), 4.44 (d, J = 9.0 Hz, 1H), 3.97 (d, J = 9.0 Hz, 1H), 3.40 (s, 1H), 1.59 (s, 3H).

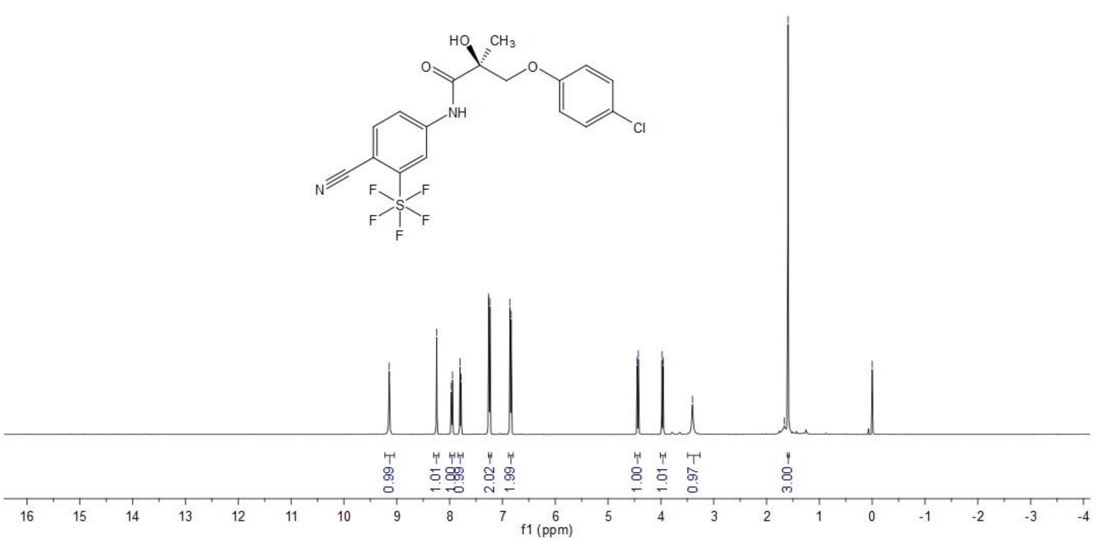

^{13}C NMR (101 MHz, $CDCl_3$) δ 172.61, 156.23, 155.58, 141.36, 136.07, 129.60, 127.10, 121.55, 119.04, 116.14, 116.07, 104.53, 75.85, 72.60, 22.96.

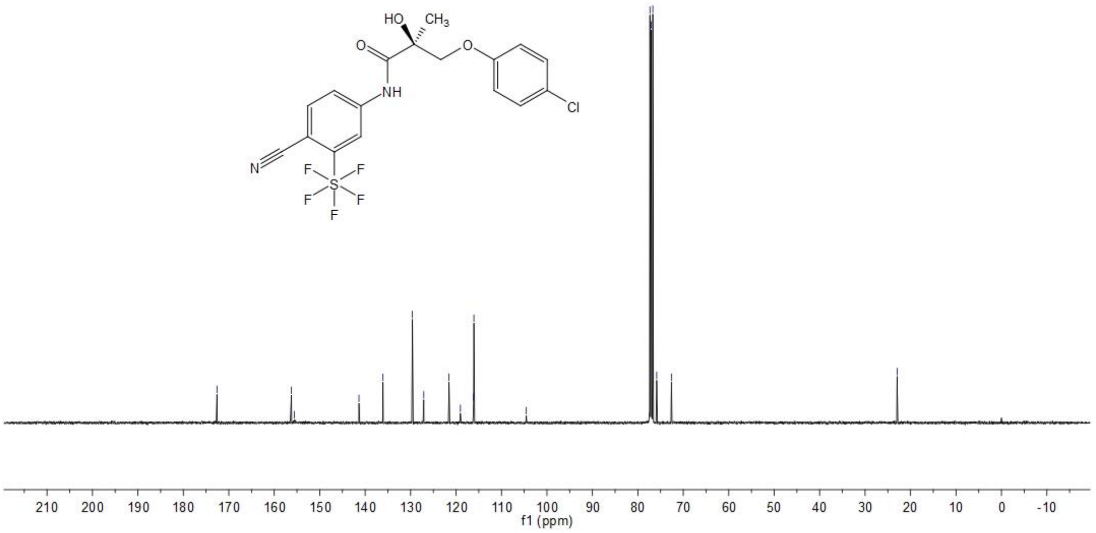

^{19}F NMR (376 MHz, CDCl$_3$) δ 81.55 – 78.71 (m, 1F), 65.49 (d, *J* = 151.3 Hz, 4F).

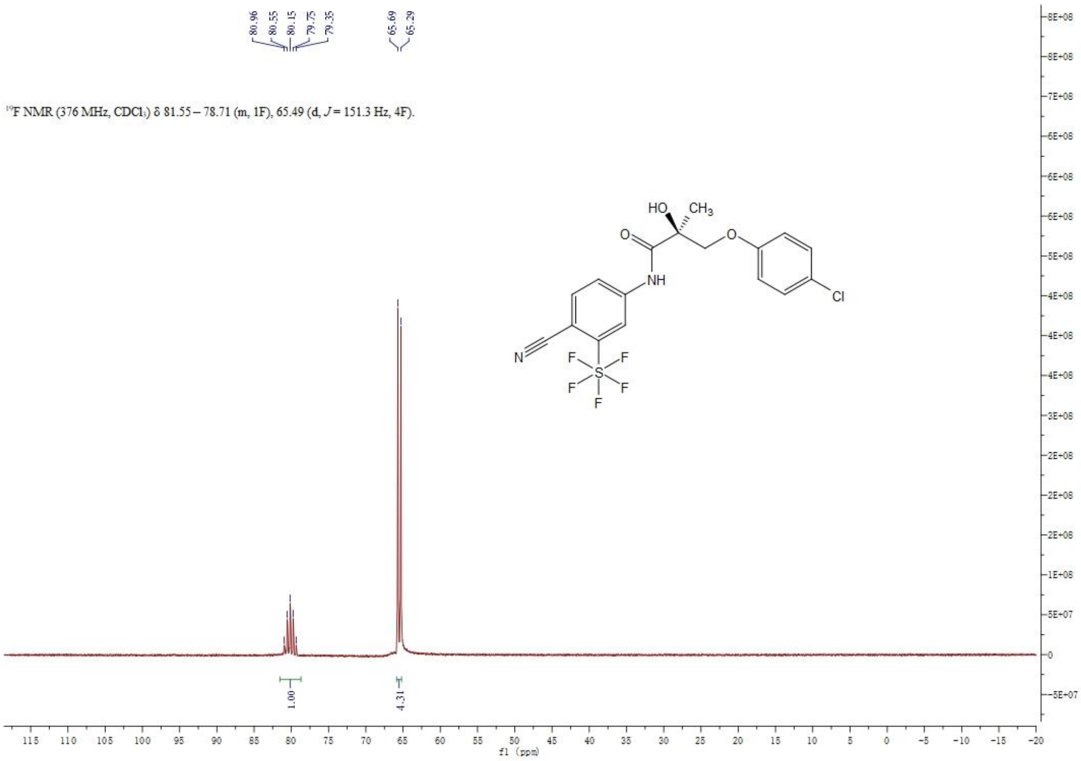

Elemental Composition Report **Page 1**

Single Mass Analysis
Tolerance = 10.0 PPM / DBE: min = -1.5, max = 50.0
Element prediction: Off

Monoisotopic Mass, Even Electron Ions
2 formula(e) evaluated with 1 results within limits (up to 50 closest results for each mass)
Elements Used:
C: 17-17 H: 15-15 N: 2-2 O: 3-3 F: 5-5 Na: 0-1 S: 1-1 Cl: 1-1
JJY-A00188-106 26 (0.537)
1: TOF MS ES+

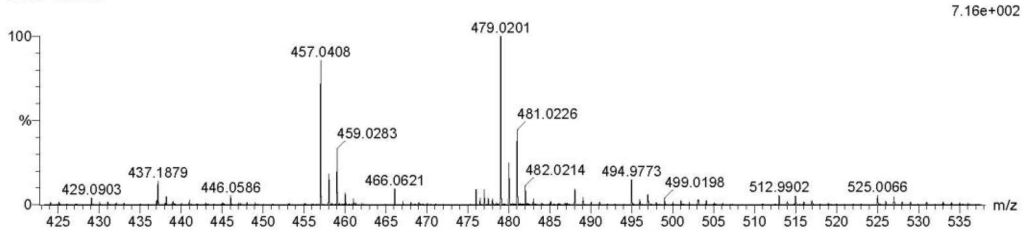

Mass	Calc. Mass	mDa	PPM	DBE	Formula
Minimum:				-1.5	
Maximum:		5.0	10.0	50.0	
457.0408	457.0412	-0.4	-0.9	8.5	C17 H15 N2 O3 F5 S Cl

数据文件: C:\CHEM32\1\DATA\JJY\LW000029.D
样品名称: JJY-A00188-106

===

操作者	: spx		
仪器	: 仪器 1	位置	: 样品瓶 1
进样日期	: 2019/11/14 13:05:12		
		进样量	: 没有进样
采集方法	: C:\CHEM32\1\METHODS\JJY-15MIN.M		
最后修改	: 2019/11/14 13:03:42 : spx (调用后修改)		
分析方法	: C:\CHEM32\1\METHODS\JJY-15MIN.M		
最后修改	: 2019/11/4 16:29:42 : CYT		

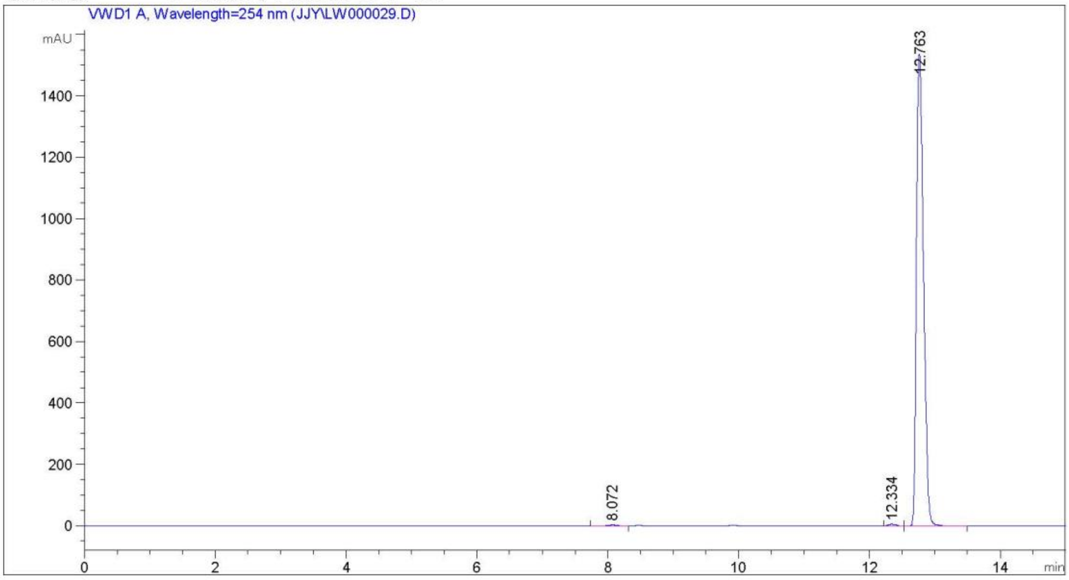

===

面积百分比报告

===

排序 : 信号
乘积因子: : 1.0000
稀释因子: : 1.0000
内标使用乘积因子和稀释因子

信号 1: VWD1 A, Wavelength=254 nm

峰 #	保留时间 [min]	类型	峰宽 [min]	峰面积 [mAU*s]	峰高 [mAU]	峰面积 %
1	8.072	BB	0.1435	23.23422	2.46337	0.2022
2	12.334	VV	0.1132	37.70036	5.23289	0.3281
3	12.763	VB	0.1159	1.14291e4	1536.36047	99.4697

总量 : 1.14900e4 1544.05674

===

仪器 1 2019/11/14 18:11:02 spx 页 1/1

^{1}H, ^{13}C, ^{19}F NMR, HPLC and HRMS spectra of *compound* **13d**

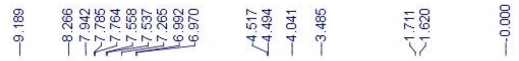

^{1}H NMR (400 MHz, $CDCl_3$) δ 9.19 (s, 1H), 8.27 (s, 1H), 7.95 (d, *J* = 8.3 Hz, 1H), 7.77 (d, *J* = 8.5 Hz, 1H), 7.55 (d, *J* = 8.4 Hz, 2H), 6.98 (d, *J* = 8.5 Hz, 2H), 4.51 (d, *J* = 9.2 Hz, 1H), 4.05 (d, *J* = 9.0 Hz, 1H), 3.49 (s, 1H), 1.62 (s, 3H).

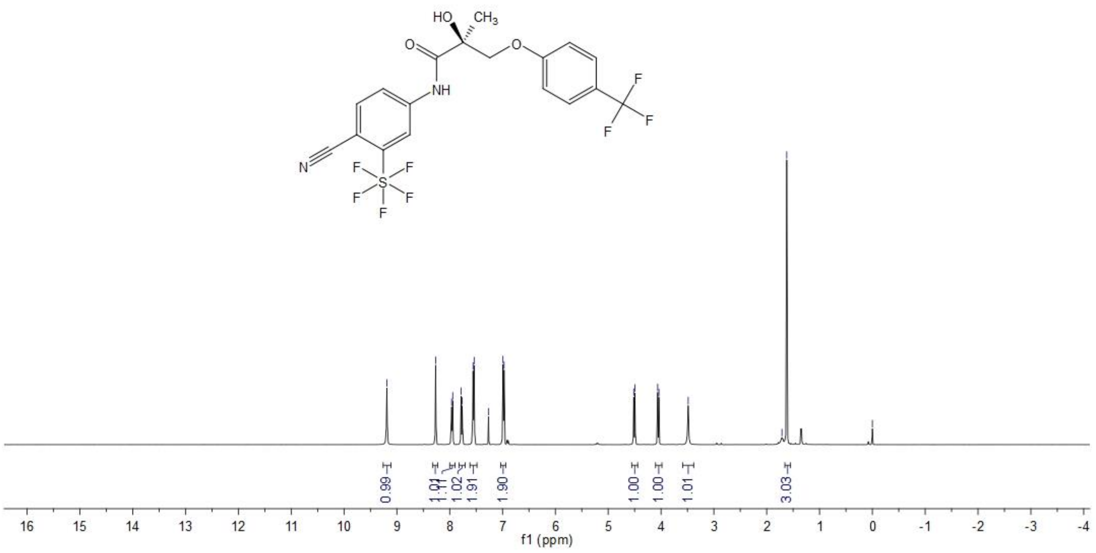

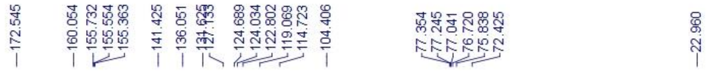

^{13}C NMR (101 MHz, $CDCl_3$) δ 172.54, 160.05, 155.73, 155.55, 155.36, 141.42, 136.05, 131.62, 127.13, 127.10, 127.06, 125.50, 124.69, 124.36, 124.03, 123.71, 122.80, 121.58, 119.07, 116.17, 114.72, 104.41, 75.84, 72.43, 22.96.

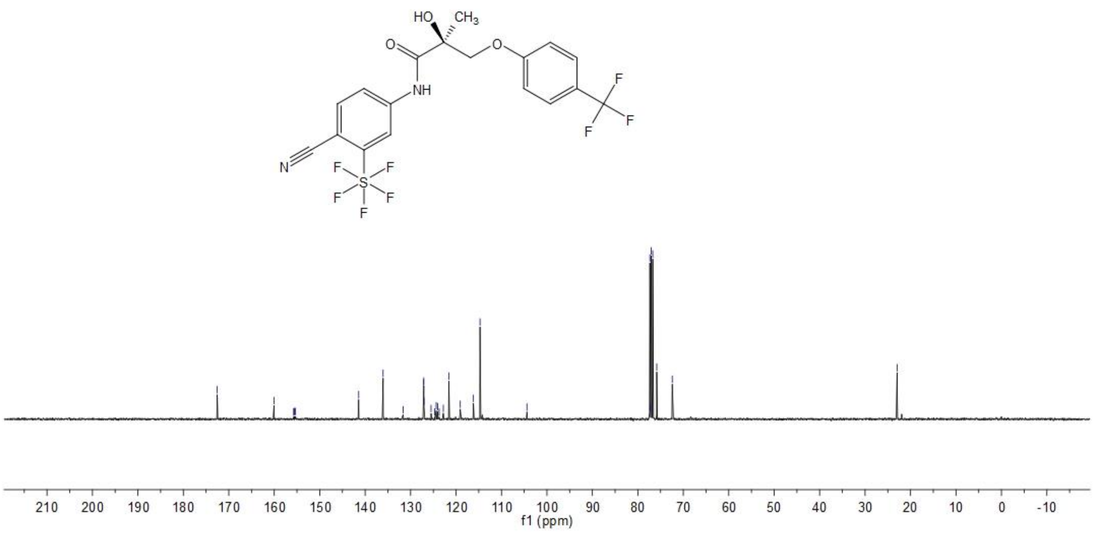

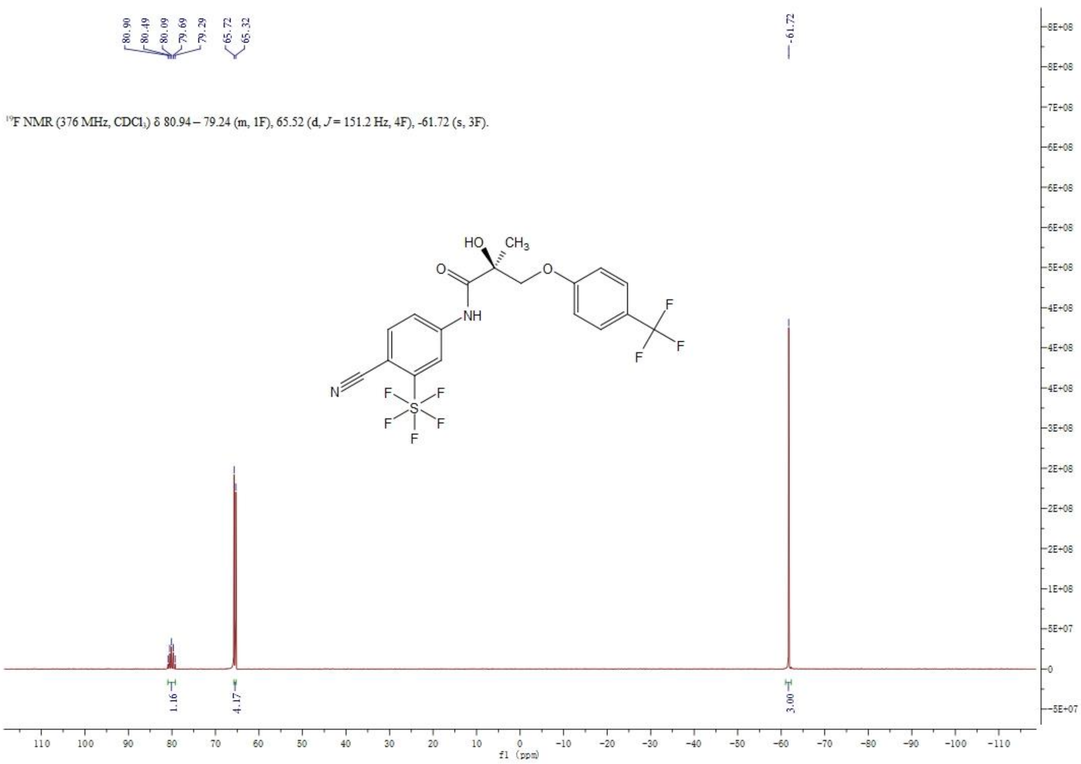

Elemental Composition Report

Page 1

Single Mass Analysis

Tolerance = 100.0 PPM / DBE: min = -1.5, max = 50.0
Element prediction: Off

Monoisotopic Mass, Even Electron Ions
1 formula(e) evaluated with 1 results within limits (up to 50 closest results for each mass)
Elements Used:
C: 18-18 H: 14-14 N: 2-2 O: 3-3 F: 8-8 S: 1-1 Na: 0-1

JJY-A00188-116 24 (0.484)
1: TOF MS ES+

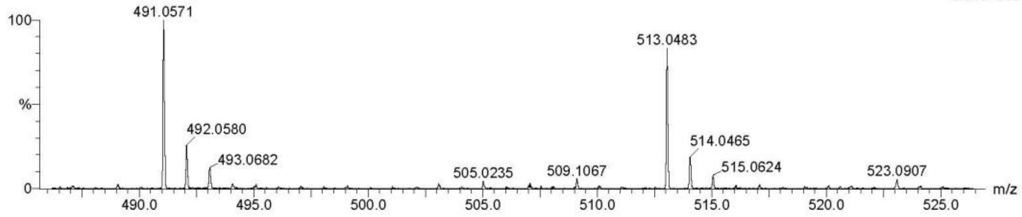

Minimum:				-1.5	
Maximum:		5.0	100.0	50.0	
Mass	Calc. Mass	mDa	PPM	DBE	Formula
513.0483	513.0495	-1.2	-2.3	8.5	C18 H14 N2 O3 F8 S Na

数据文件: C:\CHEM32\1\DATA\JJY\LW000030.D
样品名称: JJY-A00188-116

===

操作者 : spx
仪器 : 仪器 1 位置 : 样品瓶 1
进样日期 : 2019/11/14 13:52:02
进样量 : 没有进样
采集方法 : C:\CHEM32\1\METHODS\JJY-15MIN.M
最后修改 : 2019/11/14 13:50:21 : spx
(调用后修改)
分析方法 : C:\CHEM32\1\METHODS\JJY-15MIN.M
最后修改 : 2019/11/4 16:29:42 : CYT

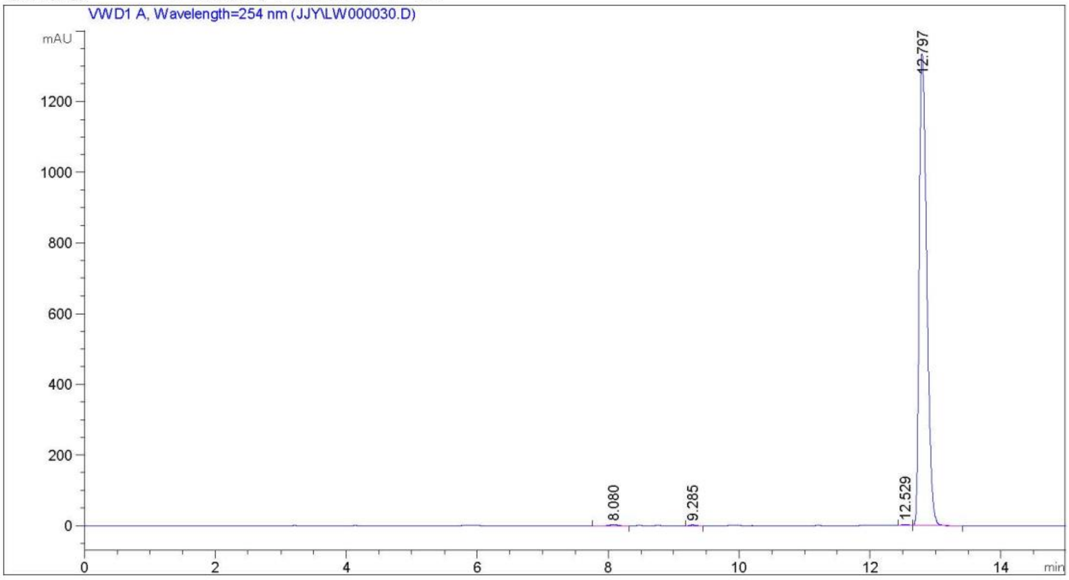

===

面积百分比报告

===

排序 : 信号
乘积因子: : 1.0000
稀释因子: : 1.0000
内标使用乘积因子和稀释因子

信号 1: VWD1 A, Wavelength=254 nm

峰 #	保留时间 [min]	类型	峰宽 [min]	峰面积 [mAU*s]	峰高 [mAU]	峰面积 %
1	8.080	BB	0.1369	23.28030	2.58923	0.2174
2	9.285	BB	0.1003	10.83080	1.73947	0.1011
3	12.529	BV	0.1121	18.02388	2.48911	0.1683
4	12.797	VB	0.1266	1.06574e4	1335.09741	99.5132

总量 : 1.07096e4 1341.91522

^{1}H, ^{13}C, ^{19}F NMR, HPLC and HRMS spectra of *compound* **13e**

^{1}H NMR (400 MHz, DMSO) δ 10.69 (s, 1H), 9.78 (s, 1H), 8.80 (s, 1H), 8.35 (d, J = 8.7 Hz, 1H), 8.12 (d, J = 8.7 Hz, 1H), 7.45 (d, J = 9.0 Hz, 2H), 6.85 (d, J = 9.0 Hz, 2H), 6.29 (s, 1H), 4.18 (d, J = 9.7 Hz, 1H), 3.95 (d, J = 9.6 Hz, 1H), 1.99 (s, 3H), 1.44 (s, 3H).

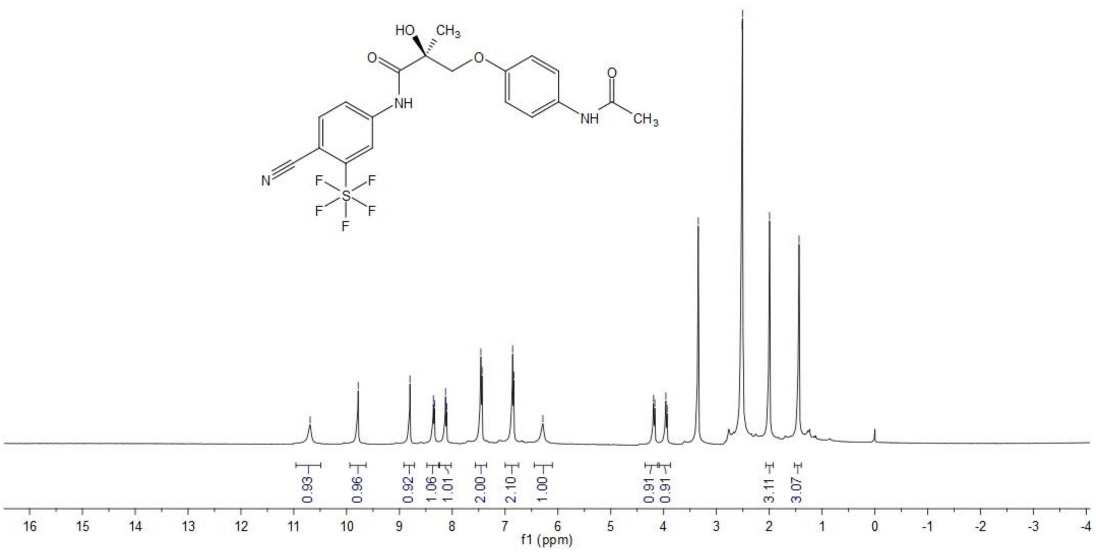

^{13}C NMR (101 MHz, DMSO) δ 174.78, 167.68, 154.14, 143.36, 136.56, 132.80, 122.40, 120.33, 118.99, 116.37, 114.59, 101.46, 74.86, 73.74, 23.76, 22.97.

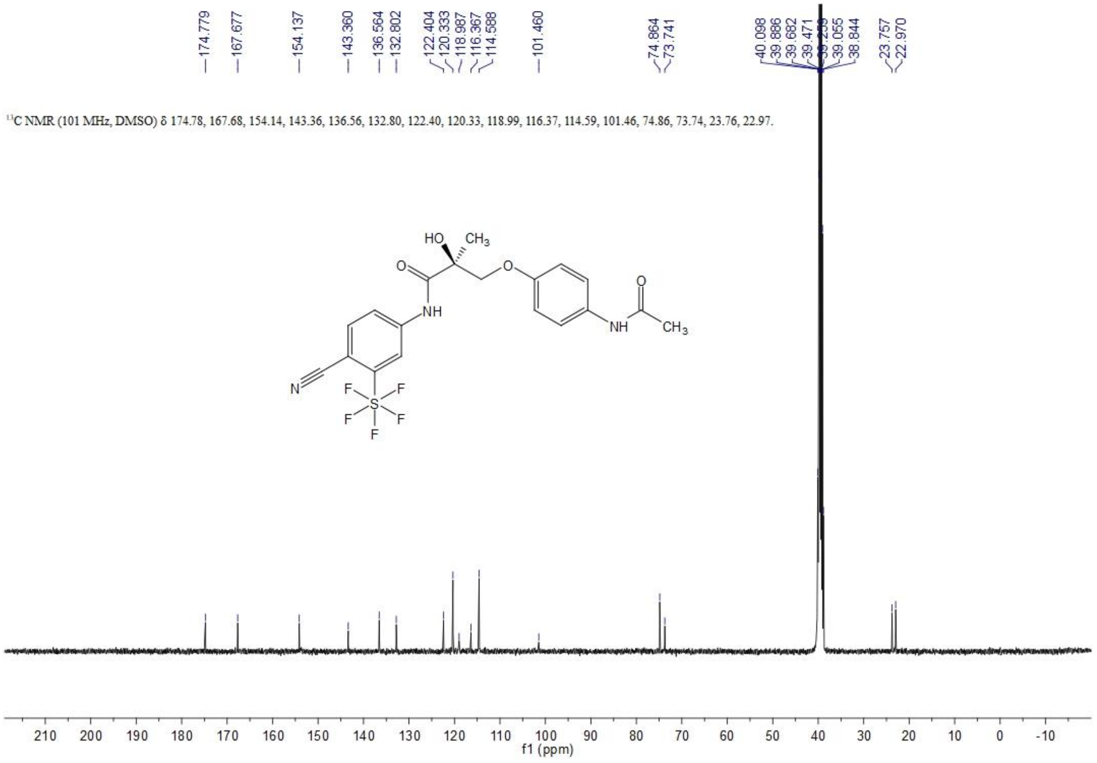

^{19}F NMR (376 MHz, DMSO) δ 83.68 (m, 1F), 66.17 (d, J = 152.0 Hz, 4F).

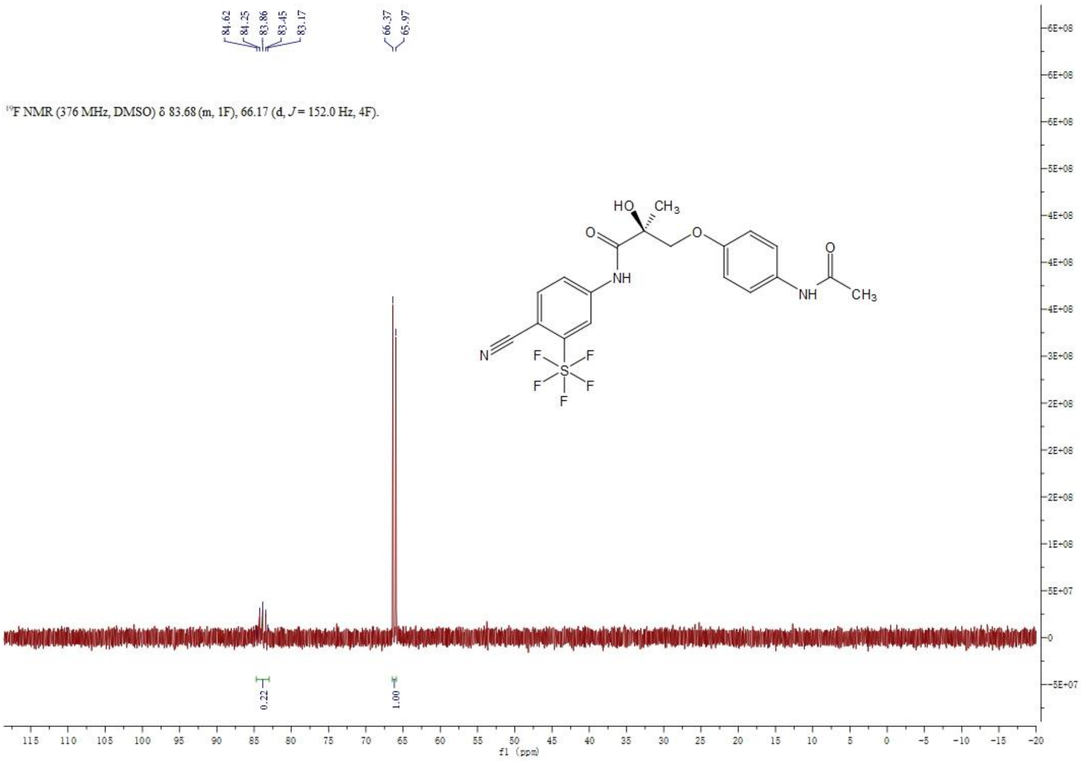

Elemental Composition Report

Page 1

Single Mass Analysis

Tolerance = 100.0 PPM / DBE: min = -1.5, max = 50.0
Element prediction: Off

Monoisotopic Mass, Even Electron Ions
1 formula(e) evaluated with 1 results within limits (up to 50 closest results for each mass)
Elements Used:
C: 19-19 H: 18-18 N: 3-3 O: 4-4 F: 5-5 Na: 0-1 S: 1-1
JJY-A00188-118 83 (1.644)
1: TOF MS ES+

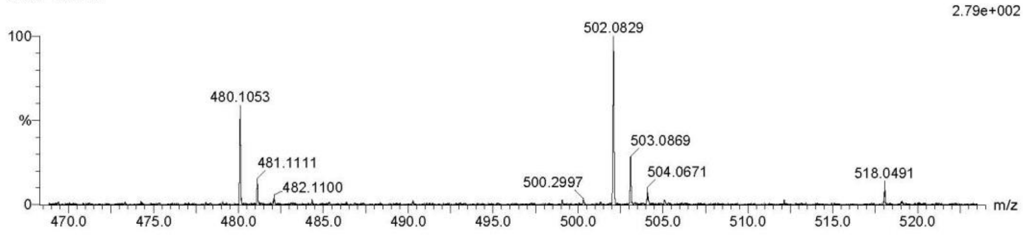

Minimum:				-1.5	
Maximum:		5.0	100.0	50.0	
Mass	Calc. Mass	mDa	PPM	DBE	Formula
502.0829	502.0836	-0.7	-1.4	9.5	C19 H18 N3 O4 F5 Na S

Data File D:\AGILENT DATA\DATA\JJY\JJY000038.D
Sample Name: JJY-A00188-118

===

Acq. Operator : JJY
Acq. Instrument : Instrument 1 Location : Vial 71
Injection Date : 4/29/2016 1:02:49 PM
Inj Volume : 15.0 µl
Acq. Method : D:\AGILENT DATA\METHOD\JJY-0.1TFA-CH3CN-15MIN.M
Last changed : 4/29/2016 1:00:53 PM by JJY
(modified after loading)
Analysis Method : D:\AGILENT DATA\METHOD\XYY-0.1TFA-CH3CN-30MIN-254NM-.M
Last changed : 4/22/2016 6:27:47 PM by XYY
Additional Info : Peak(s) manually integrated

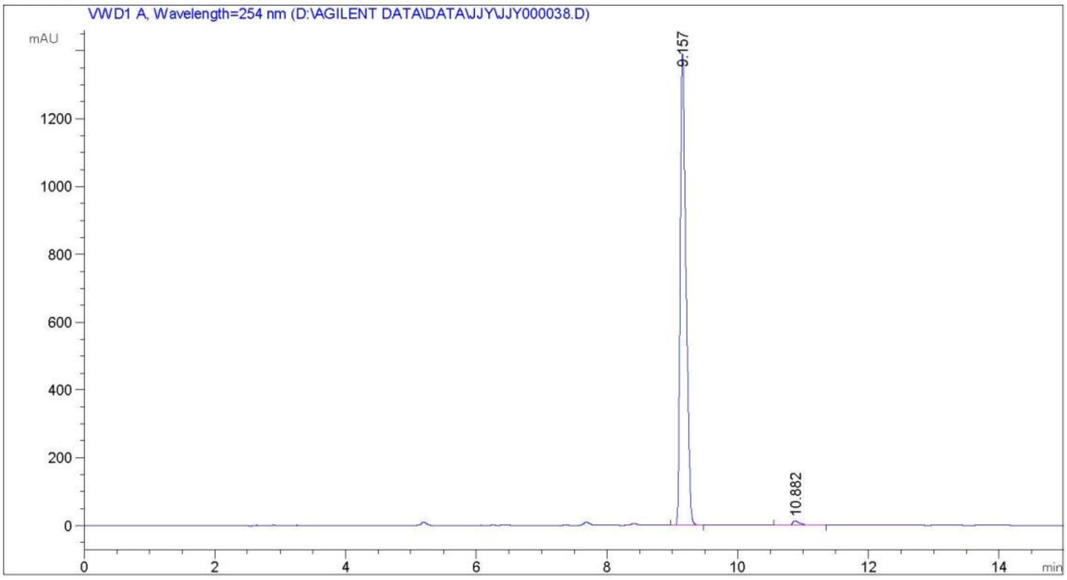

===

Area Percent Report

===

Sorted By : Signal
Multiplier : 1.0000
Dilution : 1.0000
Use Multiplier & Dilution Factor with ISTDs

Signal 1: VWD1 A, Wavelength=254 nm

Peak #	RetTime [min]	Type	Width [min]	Area mAU *s	Height [mAU]	Area %
1	9.157	BV	0.0917	8510.41406	1391.34851	98.8238
2	10.882	BV	0.1099	101.29104	13.41785	1.1762

Totals : 8611.70510 1404.76636

===

^{1}H, ^{13}C, ^{19}F NMR, HPLC and HRMS spectra of *compound* **13f**

^{1}H NMR (400 MHz, DMSO) δ 10.71 (s, 1H), 8.79 (d, J = 1.9 Hz, 1H), 8.35 (d, J = 8.7 Hz, 1H), 8.12 (d, J = 8.7 Hz, 1H), 7.91 (d, J = 8.9 Hz, 2H), 7.03 (d, J = 8.8 Hz, 2H), 6.36 (s, 1H), 4.33 (d, J = 9.9 Hz, 1H), 4.09 (d, J = 9.8 Hz, 1H), 2.96 (q, J = 7.2 Hz, 2H), 1.46 (s, 3H), 1.06 (t, J = 7.2 Hz, 3H).

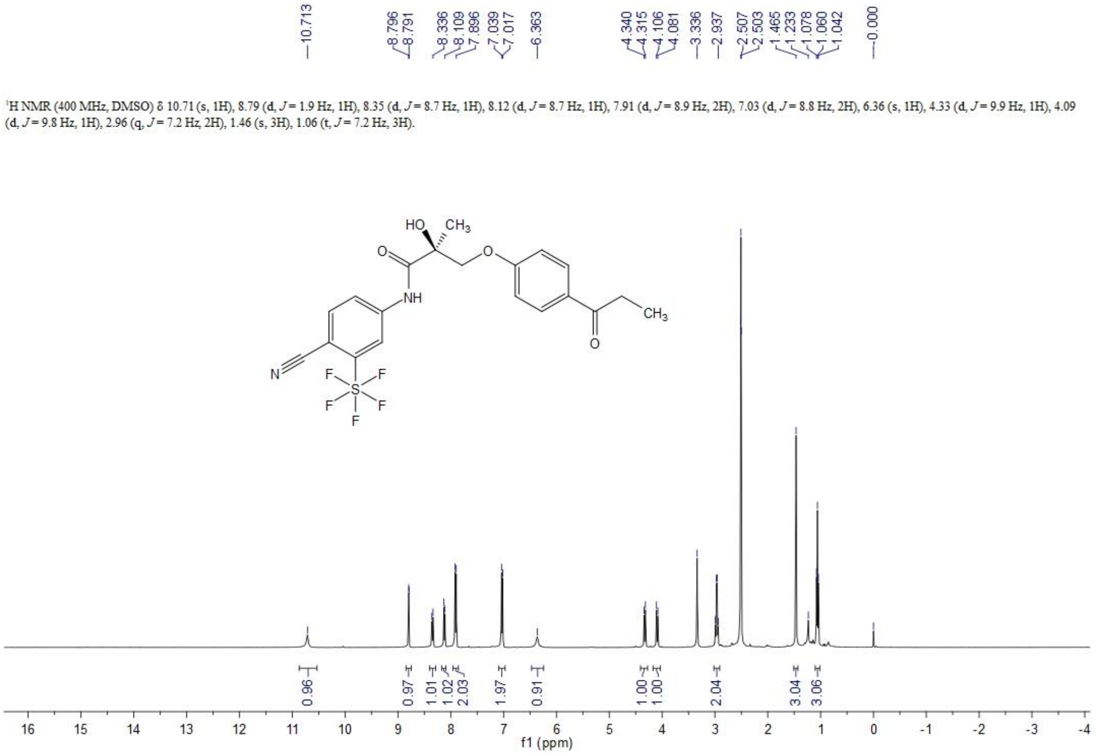

^{13}C NMR (101 MHz, DMSO) δ 198.75, 174.59, 162.06, 143.31, 136.56, 129.99, 129.71, 122.45, 119.04, 116.34, 114.41, 101.52, 74.75, 73.59, 30.75, 22.92, 8.23.

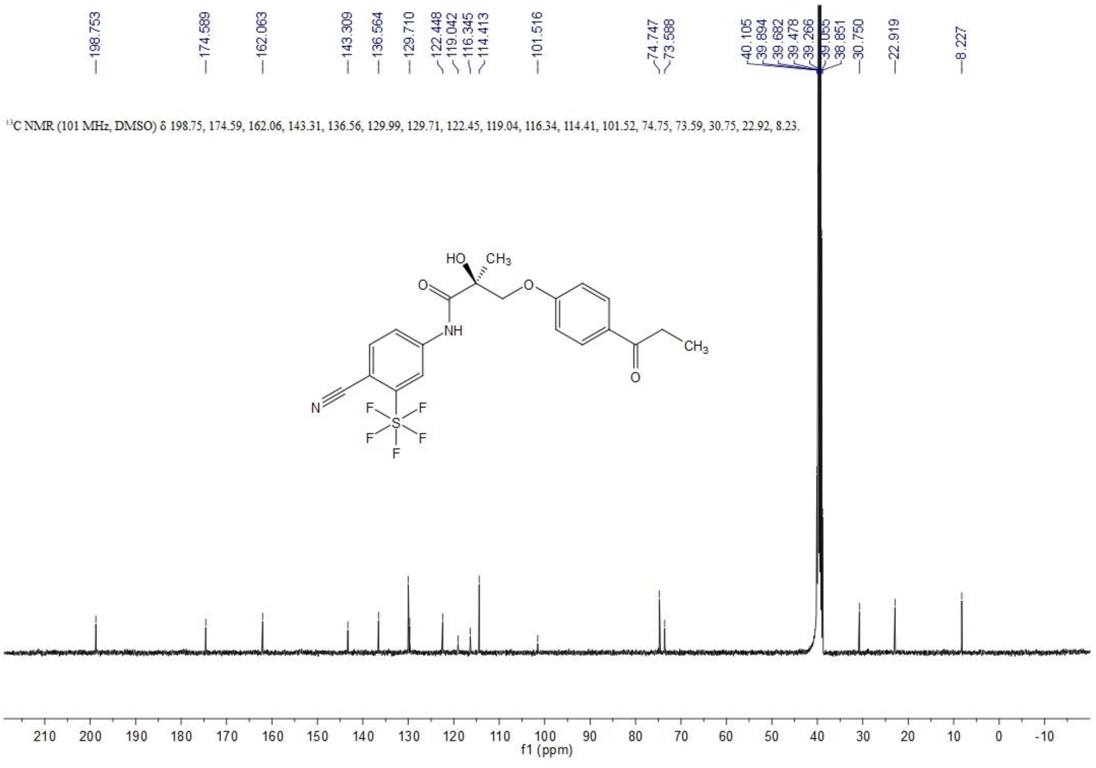

^{19}F NMR (376 MHz, DMSO) δ 83.81 (p, J = 153.6 Hz, 1F), 66.16 (d, J = 151.9 Hz, 4F).

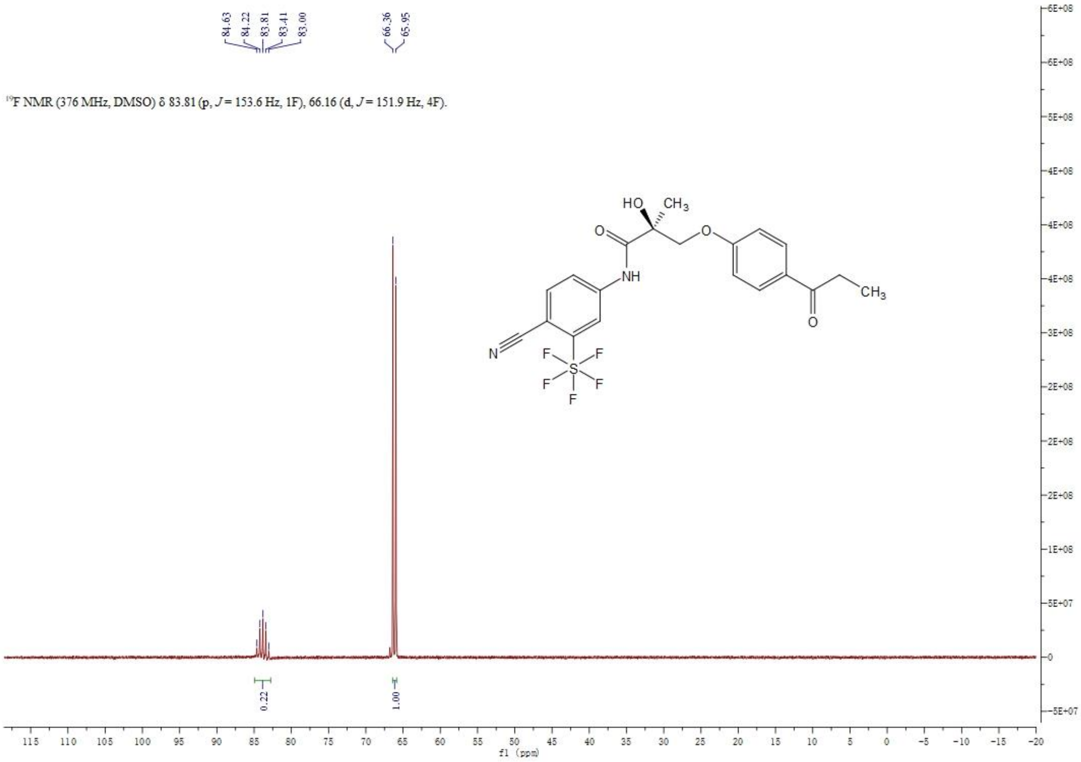

Elemental Composition Report **Page 1**

Single Mass Analysis
Tolerance = 100.0 PPM / DBE: min = -1.5, max = 50.0
Element prediction: Off

Monoisotopic Mass, Even Electron Ions
1 formula(e) evaluated with 1 results within limits (up to 50 closest results for each mass)
Elements Used:
C: 20-20 H: 19-19 N: 2-2 O: 4-4 F: 5-5 Na: 0-1 S: 1-1
JJY-A00188-110 70 (1.384)
1: TOF MS ES+

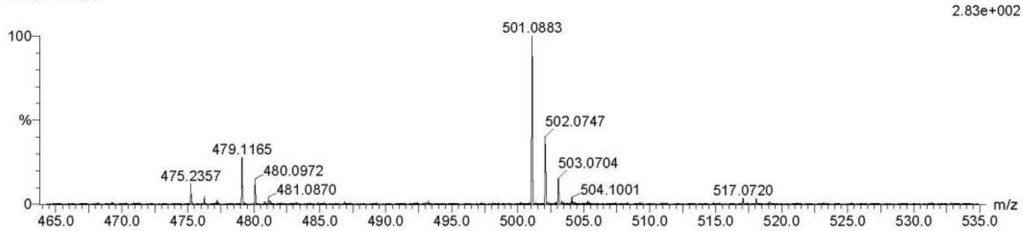

Minimum:				-1.5	
Maximum:		5.0	100.0	50.0	
Mass	Calc. Mass	mDa	PPM	DBE	Formula
501.0883	501.0883	0.0	0.0	9.5	C20 H19 N2 O4 F5 Na S

数据文件：C:\CHEM32\1\DATA\JJY\LW000034.D
样品名称：JJY-A00188-110

===

操作者　　　　　: spx
仪器　　　　　　: 仪器 1　　　　　　　　　　位置 : 样品瓶 1
进样日期　　　　: 2019/11/14 14:56:15
　　　　　　　　　　　　　　　　　　　　　　进样量 : 没有进样
采集方法　　　　: C:\CHEM32\1\METHODS\JJY-15MIN.M
最后修改　　　　: 2019/11/14 14:55:10 : spx
　　　　　　　　　(调用后修改)
分析方法　　　　: C:\CHEM32\1\METHODS\JJY-15MIN.M
最后修改　　　　: 2019/11/4 16:29:42 : CYT
附加信息：峰已手动积分

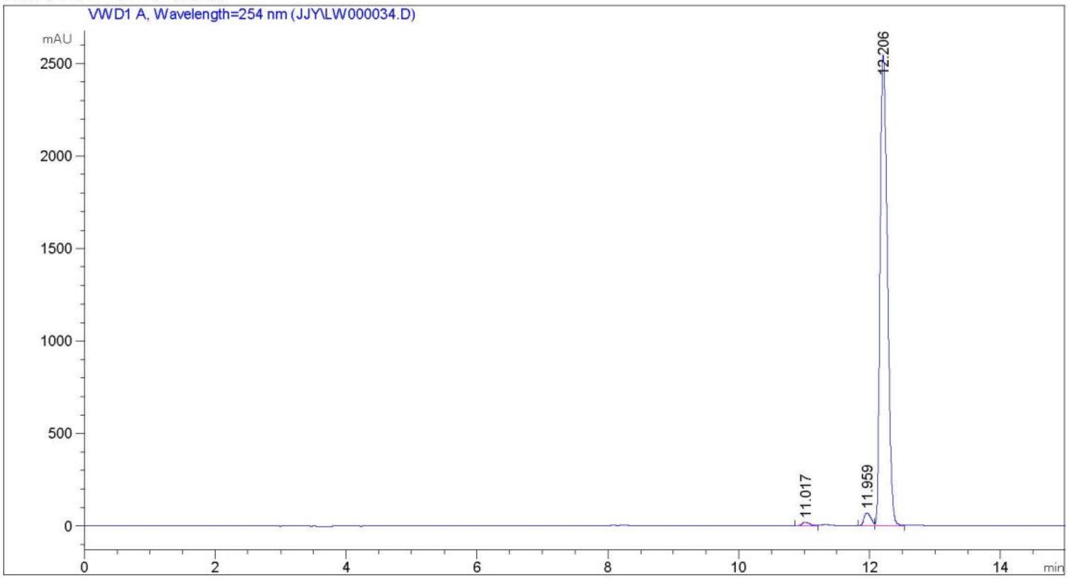

===

面积百分比报告

===

排序　　　　　　: 信号
乘积因子:　　　　: 1.0000
稀释因子:　　　　: 1.0000
内标使用乘积因子和稀释因子

信号 1: VWD1 A, Wavelength=254 nm

峰 #	保留时间 [min]	类型	峰宽 [min]	峰面积 [mAU*s]	峰高 [mAU]	峰面积 %
1	11.017	BV	0.1276	155.63445	19.28533	0.7636
2	11.959	BV	0.1160	506.72784	70.39314	2.4863
3	12.206	VV	0.1238	1.97186e4	2548.52173	96.7501

总量 :　　2.03809e4　2638.20020

^{1}H, ^{13}C, ^{19}F NMR, HPLC and HRMS spectra of *compound* **13g**

^{1}H NMR (400 MHz, $CDCl_3$) δ 9.24 (s, 1H), 8.26 (s, 1H), 7.93 (d, J = 8.5 Hz, 1H), 7.76 (d, J = 8.4 Hz, 1H), 6.93 (t, J = 9.1 Hz, 1H), 6.64 (d, J = 12.7 Hz, 1H), 6.59 (d, J = 8.9 Hz, 1H), 4.45 (d, J = 9.2 Hz, 1H), 3.97 (d, J = 9.2 Hz, 1H), 3.84 (s, 1H), 3.74 (s, 3H), 1.57 (s, 3H).

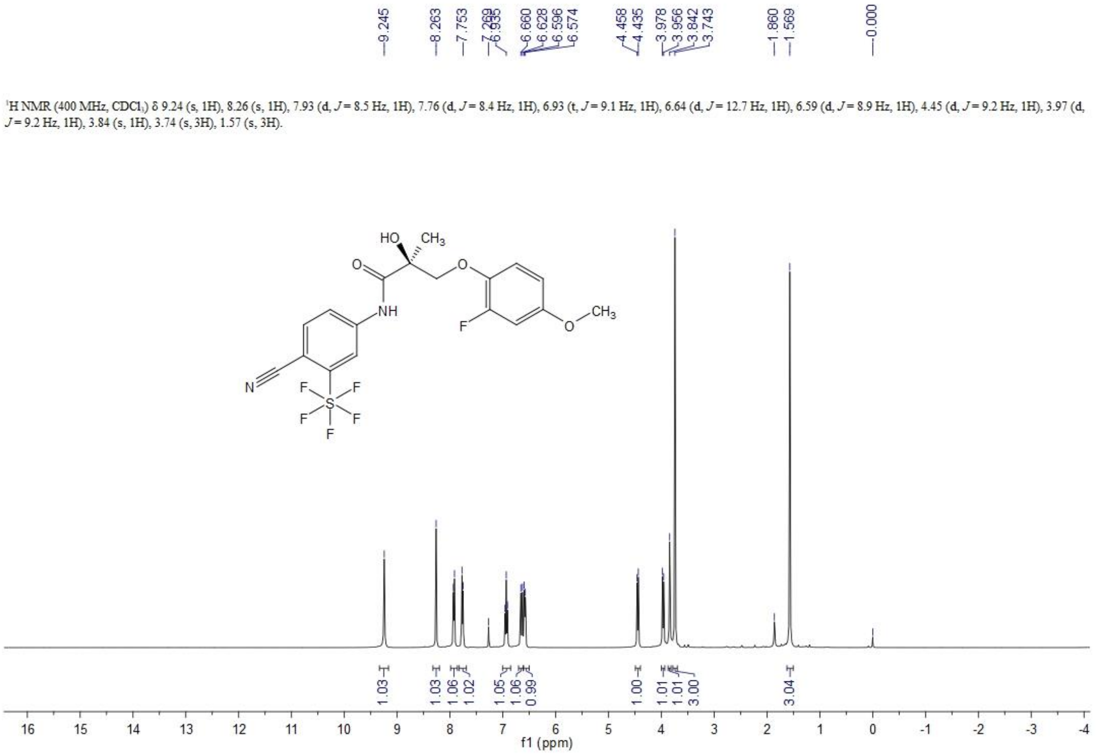

^{13}C NMR (101 MHz, $CDCl_3$) δ 173.15, 155.56, 155.47, 155.28, 154.60, 152.16, 141.55, 139.54, 139.43, 135.97, 121.66, 119.13, 118.08, 118.06, 116.22, 109.20, 109.16, 104.26, 103.35, 103.14, 75.68, 75.54, 55.79, 22.85.

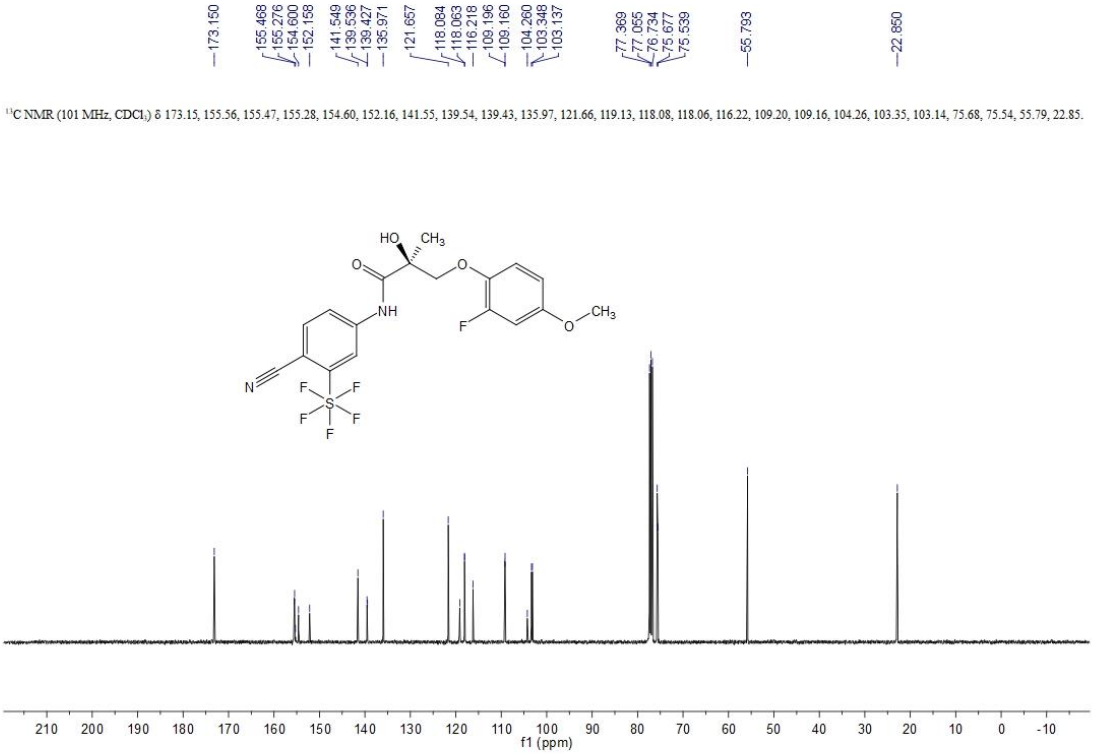

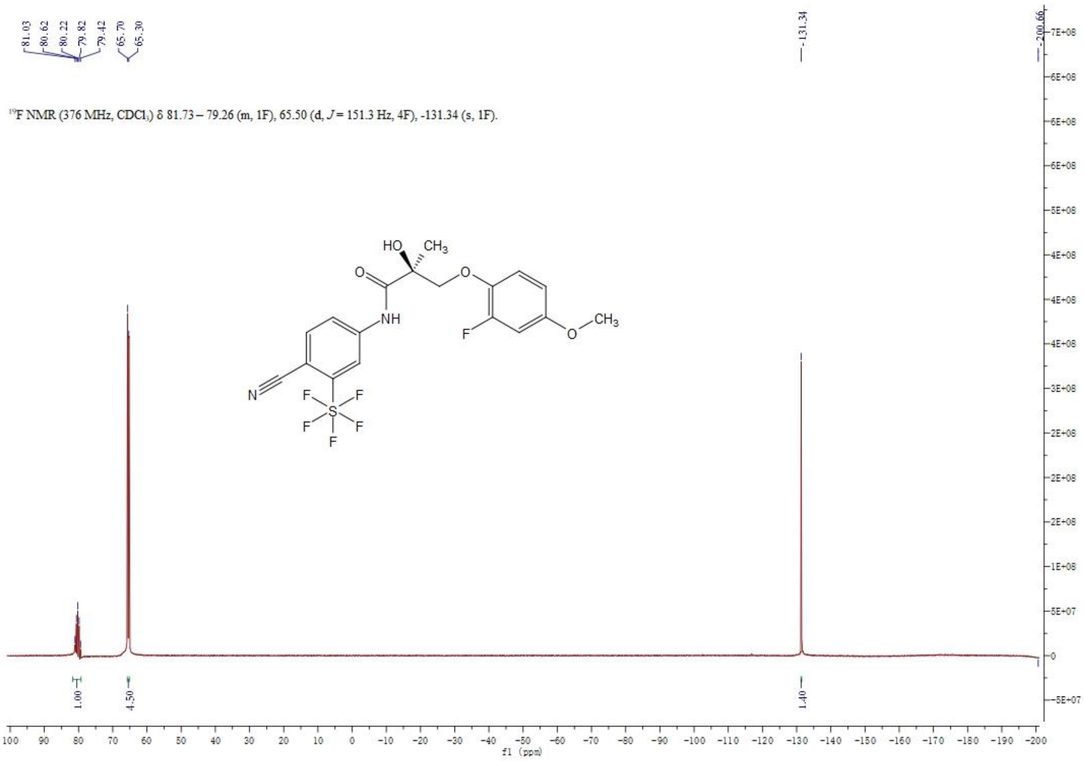

Elemental Composition Report **Page 1**

Single Mass Analysis
Tolerance = 100.0 PPM / DBE: min = -1.5, max = 50.0
Element prediction: Off

Monoisotopic Mass, Even Electron Ions
1 formula(e) evaluated with 1 results within limits (up to 50 closest results for each mass)
Elements Used:
C: 18-18 H: 16-17 N: 2-2 O: 4-4 F: 6-6 Na: 0-1 S: 1-1
JJY-A00188-108 32 (0.639)
1: TOF MS ES+

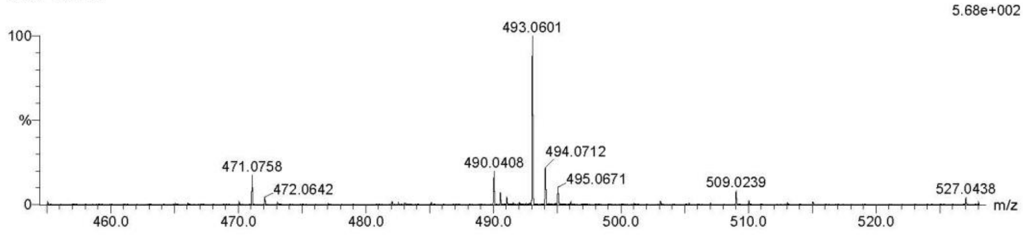

Minimum:				-1.5	
Maximum:		5.0	100.0	50.0	
Mass	Calc. Mass	mDa	PPM	DBE	Formula
493.0601	493.0633	-3.2	-6.5	8.5	C18 H16 N2 O4 F6 Na S

数据文件: C:\CHEM32\1\DATA\JJY\LW000033.D
样品名称: JJY-A00188-108

===

操作者	: spx		
仪器	: 仪器 1	位置	: 样品瓶 1
进样日期	: 2019/11/14 14:35:12		
		进样量	: 没有进样
采集方法	: C:\CHEM32\1\METHODS\JJY-15MIN.M		
最后修改	: 2019/11/14 14:33:37 : spx<br>(调用后修改)		
分析方法	: C:\CHEM32\1\METHODS\JJY-15MIN.M		
最后修改	: 2019/11/4 16:29:42 : CYT		

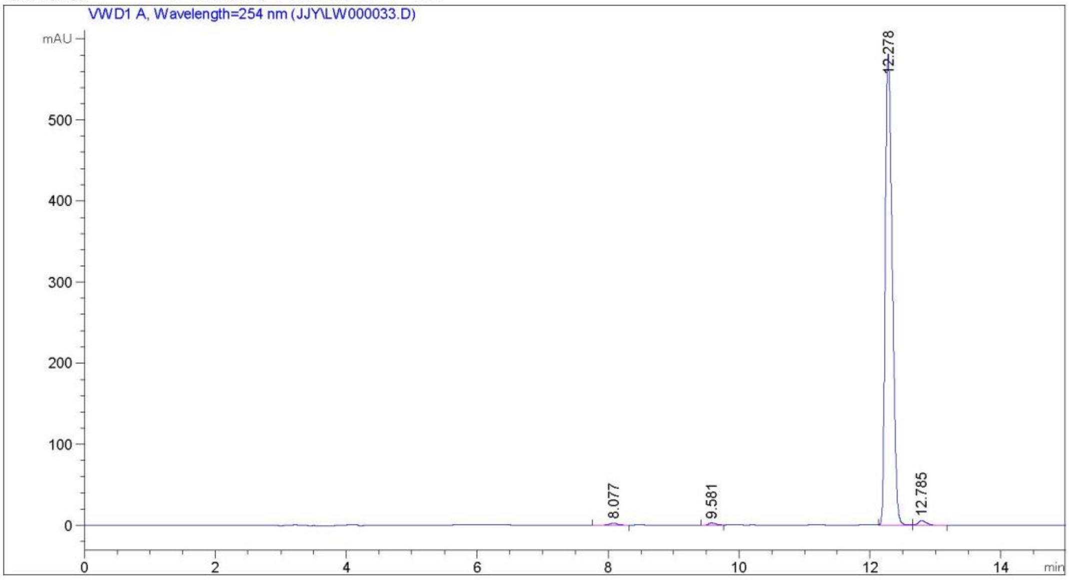

===

面积百分比报告

===

排序 : 信号
乘积因子: : 1.0000
稀释因子: : 1.0000
内标使用乘积因子和稀释因子

信号 1: VWD1 A, Wavelength=254 nm

峰 #	保留时间 [min]	类型	峰宽 [min]	峰面积 [mAU*s]	峰高 [mAU]	峰面积 %
1	8.077	BB	0.1347	22.07032	2.50782	0.4985
2	9.581	BV	0.1136	21.32775	2.94525	0.4818
3	12.278	VV	0.1175	4330.63037	581.43524	97.8211
4	12.785	VB	0.1362	53.06511	6.02596	1.1986

总量 : 4427.09355 592.91427

仪器 1 2019/11/14 18:09:30 spx 页 1/2

^{1}H, ^{13}C, ^{19}F NMR, HPLC and HRMS spectra of *compound* **16a**

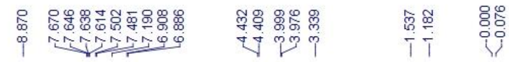

^{1}H NMR (400 MHz, $CDCl_3$) δ 8.87 (s, 1H), 7.68 – 7.59 (m, 4H), 7.49 (d, *J* = 8.5 Hz, 2H), 6.90 (d, *J* = 8.5 Hz, 2H), 4.42 (d, *J* = 9.2 Hz, 1H), 3.99 (d, *J* = 9.2 Hz, 1H), 3.34 (s, 1H), 1.54 (s, 3H).

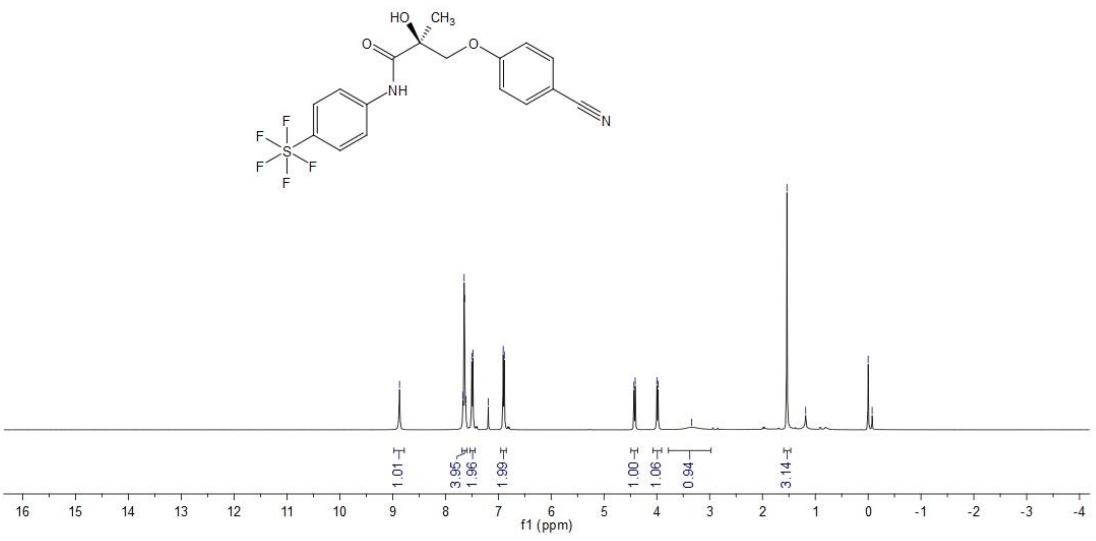

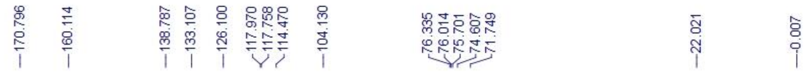

^{13}C NMR (101 MHz, $CDCl_3$) δ 170.80, 160.11, 138.79, 133.11, 126.10, 117.97, 117.76, 114.47, 104.13, 74.61, 71.75, 22.02.

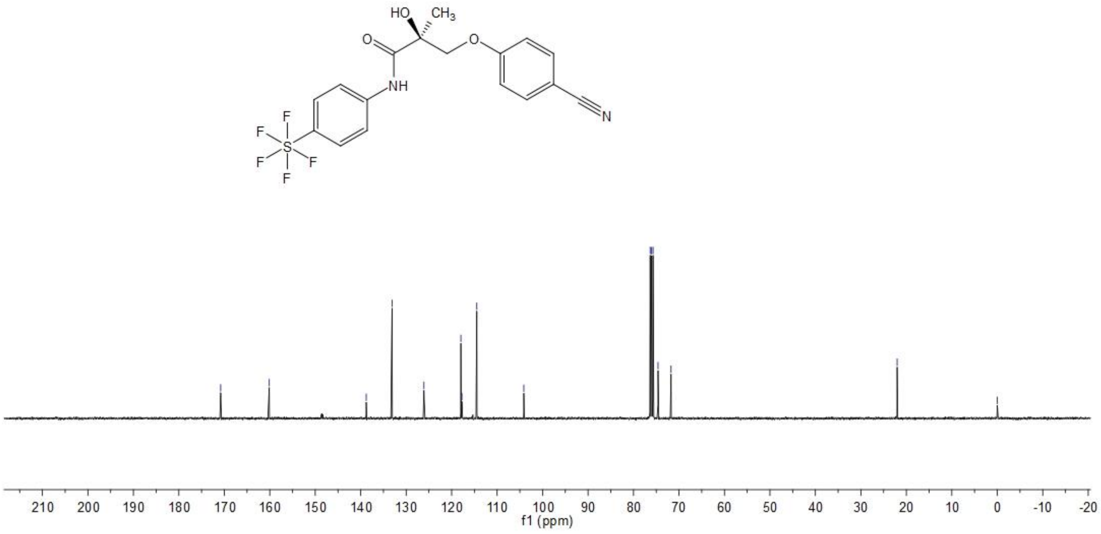

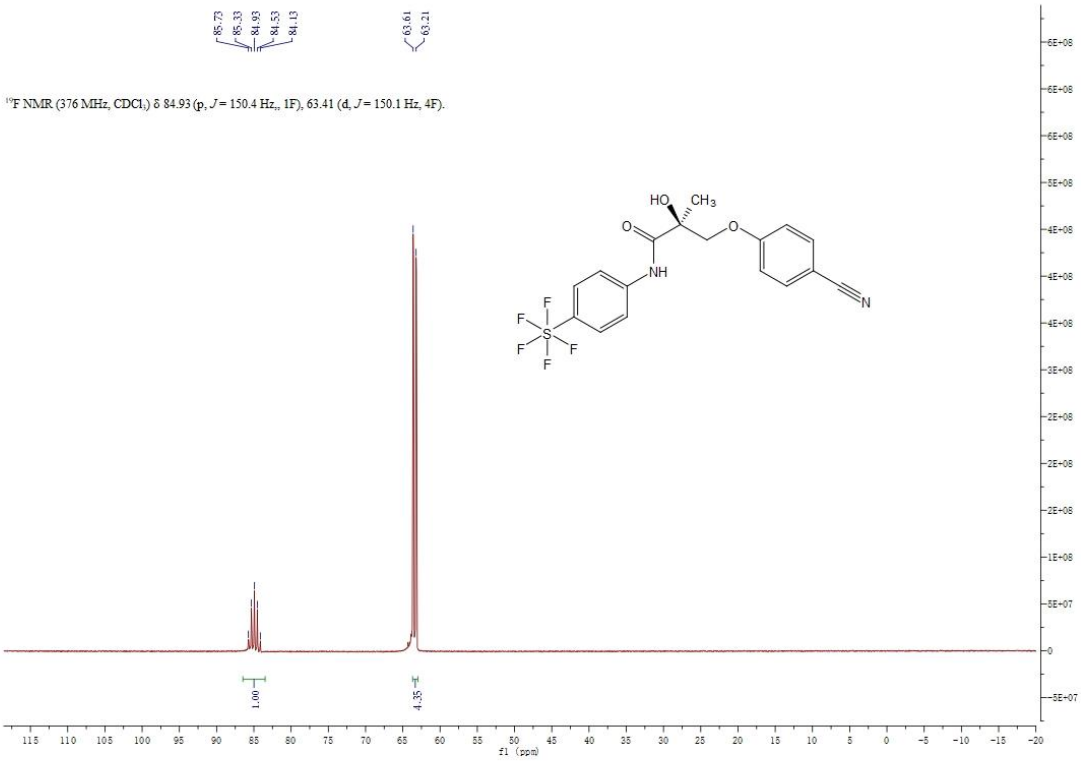

Elemental Composition Report

Page 1

Single Mass Analysis
Tolerance = 100.0 PPM / DBE: min = -1.5, max = 50.0
Element prediction: Off

Monoisotopic Mass, Even Electron Ions
1 formula(e) evaluated with 1 results within limits (up to 50 closest results for each mass)
Elements Used:
C: 17-17 H: 16-16 N: 2-2 O: 3-3 F: 5-5 S: 1-1
JJY-A00188-071 35 (0.710)
1: TOF MS ES+

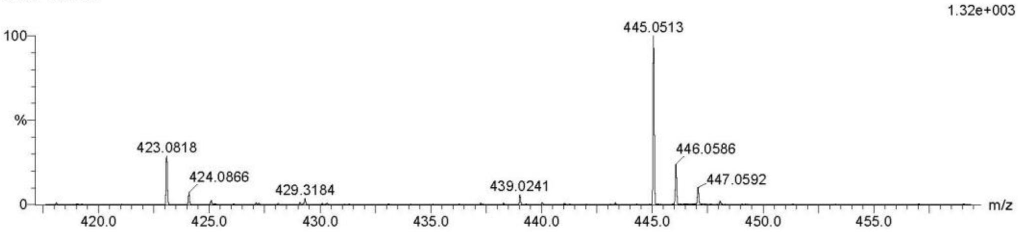

Minimum:				-1.5	
Maximum:		5.0	100.0	50.0	
Mass	Calc. Mass	mDa	PPM	DBE	Formula
423.0818	423.0802	1.6	3.8	8.5	C17 H16 N2 O3 F5 S

Data File D:\AGILENT DATA\DATA\JJY\JJY20160203 2016-03-02 11-21-19\JJY0000001.D
Sample Name: JJY-A00188-071

===

Acq. Operator	: JJY	Seq. Line :	1
Acq. Instrument	: Instrument 1	Location :	Vial 82
Injection Date	: 3/2/2016 11:22:52 AM	Inj :	1
		Inj Volume :	10.0 µl

Acq. Method : D:\AGILENT DATA\DATA\JJY\JJY20160203 2016-03-02 11-21-19\JJY-0.1TFA-CH3CN-15MIN.M
Last changed : 3/2/2016 10:11:59 AM by JJY
Analysis Method : D:\AGILENT DATA\METHOD\XXY-PTX.M
Last changed : 11/6/2019 2:57:04 PM by HY
Additional Info : Peak(s) manually integrated

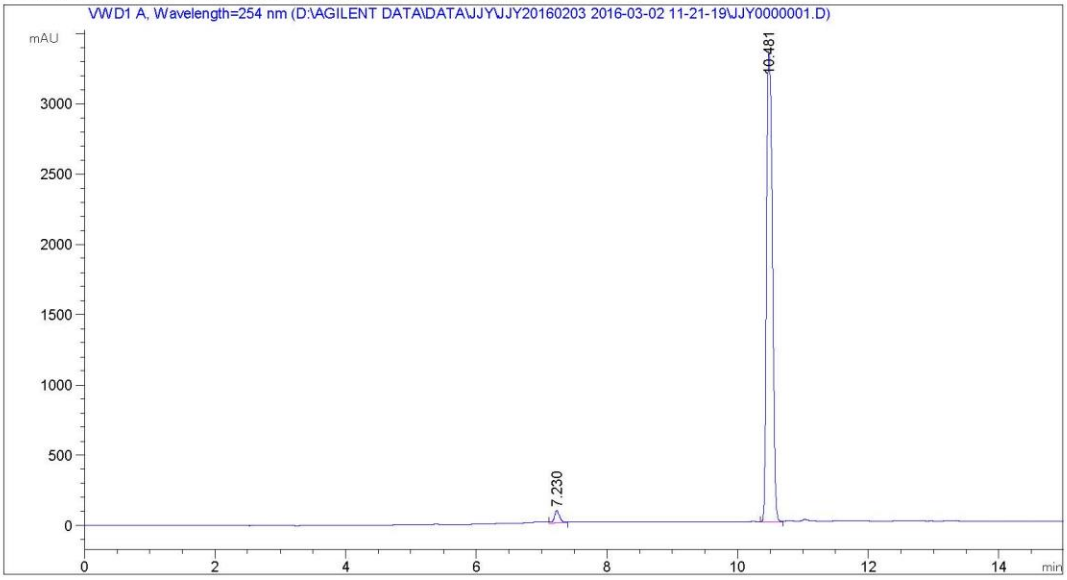

===

Area Percent Report

===

Sorted By : Signal
Multiplier : 1.0000
Dilution : 1.0000
Use Multiplier & Dilution Factor with ISTDs

Signal 1: VWD1 A, Wavelength=254 nm

Peak #	RetTime [min]	Type	Width [min]	Area mAU *s	Height [mAU]	Area %
1	7.230	VV	0.0838	504.59473	88.70807	2.3702
2	10.481	BV	0.1019	2.07847e4	3331.57300	97.6298

Totals : 2.12893e4 3420.28107

===

^{1}H, ^{13}C, ^{19}F NMR, HPLC and HRMS spectra of *compound* **16b**

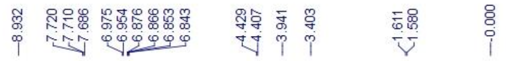

^{1}H NMR (400 MHz, $CDCl_3$) δ 8.93 (s, 1H), 7.73 (d, J = 9.5 Hz, 2H), 7.70 (d, J = 9.8 Hz, 2H), 6.98 (t, J = 8.5 Hz, 2H), 6.86 (dd, J = 9.1, 4.1 Hz, 2H), 4.42 (d, J = 9.0 Hz, 1H), 3.95 (d, J = 8.9 Hz, 1H), 3.40 (s, 1H), 1.58 (s, 3H).

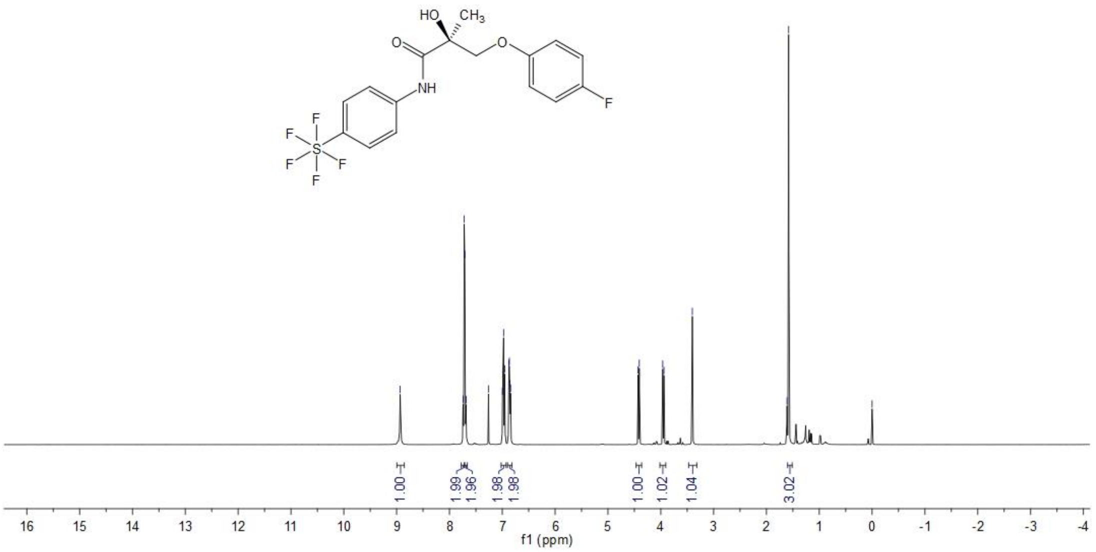

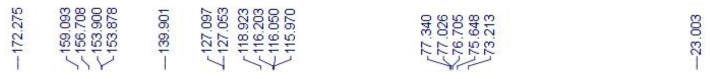

^{13}C NMR (101 MHz, $CDCl_3$) δ 172.27, 159.09, 156.71, 153.90, 153.88, 139.90, 127.14, 127.10, 127.05, 118.92, 116.20, 116.05, 115.97, 75.65, 73.21, 23.00.

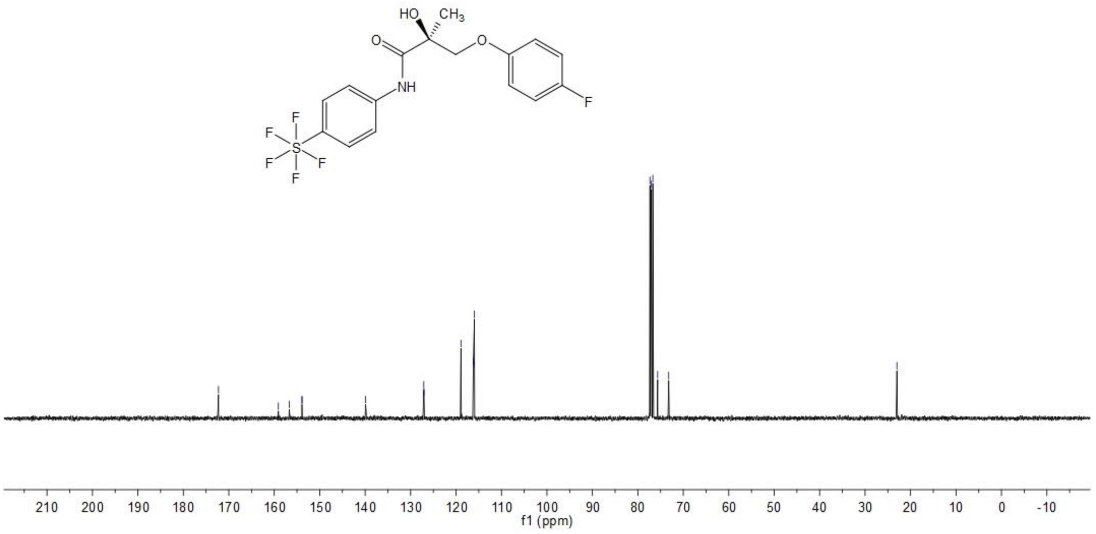

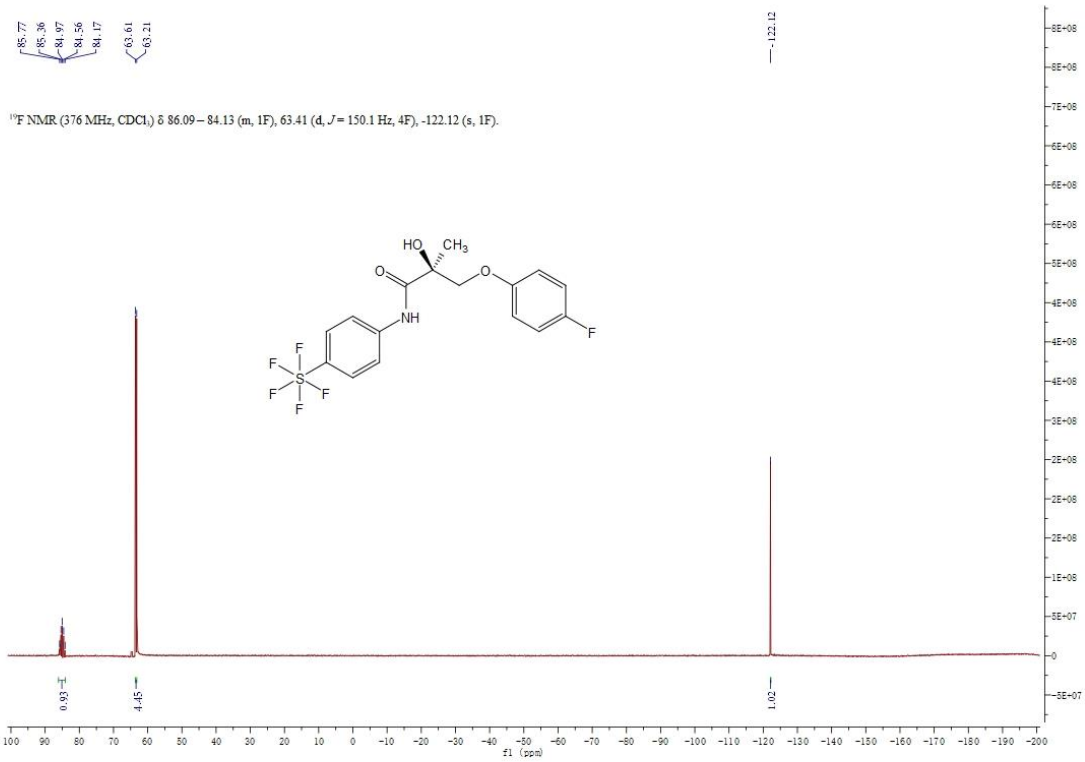

Elemental Composition Report

Page 1

Single Mass Analysis

Tolerance = 100.0 PPM / DBE: min = -1.5, max = 50.0
Element prediction: Off

Monoisotopic Mass, Even Electron Ions
1 formula(e) evaluated with 1 results within limits (up to 50 closest results for each mass)
Elements Used:
C: 16-16 H: 16-16 N: 1-1 O: 3-3 F: 6-6 S: 1-1
JJY-A00188-072 35 (0.710)
1: TOF MS ES+

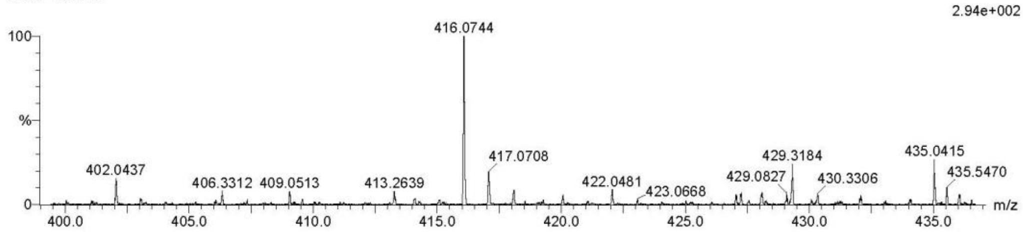

Minimum:				-1.5	
Maximum:		5.0	100.0	50.0	
Mass	Calc. Mass	mDa	PPM	DBE	Formula
416.0744	416.0755	-1.1	-2.6	6.5	C16 H16 N O3 F6 S

Data File D:\AGILENT DATA\DATA\JJY\JJY20160203 2016-03-11 17-01-08\JJY0000002.D
Sample Name: JJY-A00188-072

===

Acq. Operator : JJY Seq. Line : 2
Acq. Instrument : Instrument 1 Location : Vial 82
Injection Date : 3/11/2016 5:24:12 PM Inj : 1
Inj Volume : 10.0 µl
Different Inj Volume from Sequence ! Actual Inj Volume : 5.0 µl
Acq. Method : D:\AGILENT DATA\DATA\JJY\JJY20160203 2016-03-11 17-01-08\JJY-0.1TFA-CH3CN-15MIN.M
Last changed : 3/11/2016 4:14:26 PM by JJY
Analysis Method : D:\AGILENT DATA\METHOD\JJY-0.1TFA-CH3CN-15MIN.M
Last changed : 3/2/2016 10:11:59 AM by JJY
Additional Info : Peak(s) manually integrated

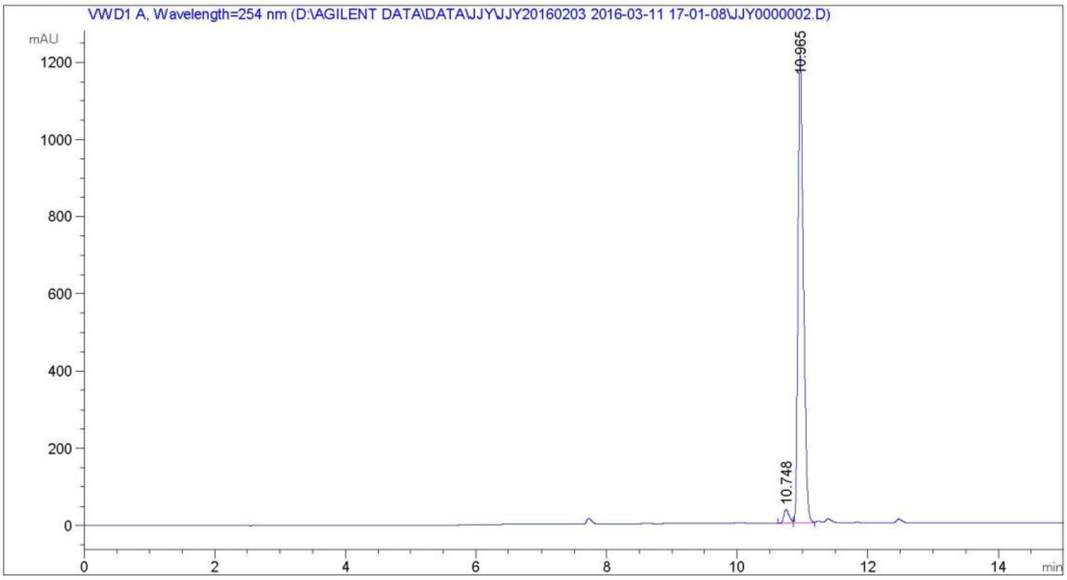

===

Area Percent Report

===

Sorted By : Signal
Multiplier : 1.0000
Dilution : 1.0000
Use Multiplier & Dilution Factor with ISTDs

Signal 1: VWD1 A, Wavelength=254 nm

Peak #	RetTime [min]	Type	Width [min]	Area mAU *s	Height [mAU]	Area %
1	10.748	BV	0.0848	201.79230	35.73984	2.8204
2	10.965	VV	0.0857	6952.95068	1215.25793	97.1796

Totals : 7154.74298 1250.99778

^{1}H, ^{13}C, ^{19}F NMR, HPLC and HRMS spectra of *compound* **16c**

^{1}H NMR (400 MHz, $CDCl_3$) δ 8.94 (s, 1H), 7.70 (q, *J* = 9.5 Hz, 4H), 7.22 (d, *J* = 8.5 Hz, 2H), 6.83 (d, *J* = 8.5 Hz, 2H), 4.41 (d, *J* = 8.9 Hz, 1H), 3.95 (d, *J* = 9.0 Hz, 1H), 3.48 (s, 1H), 1.58 (s, 3H).

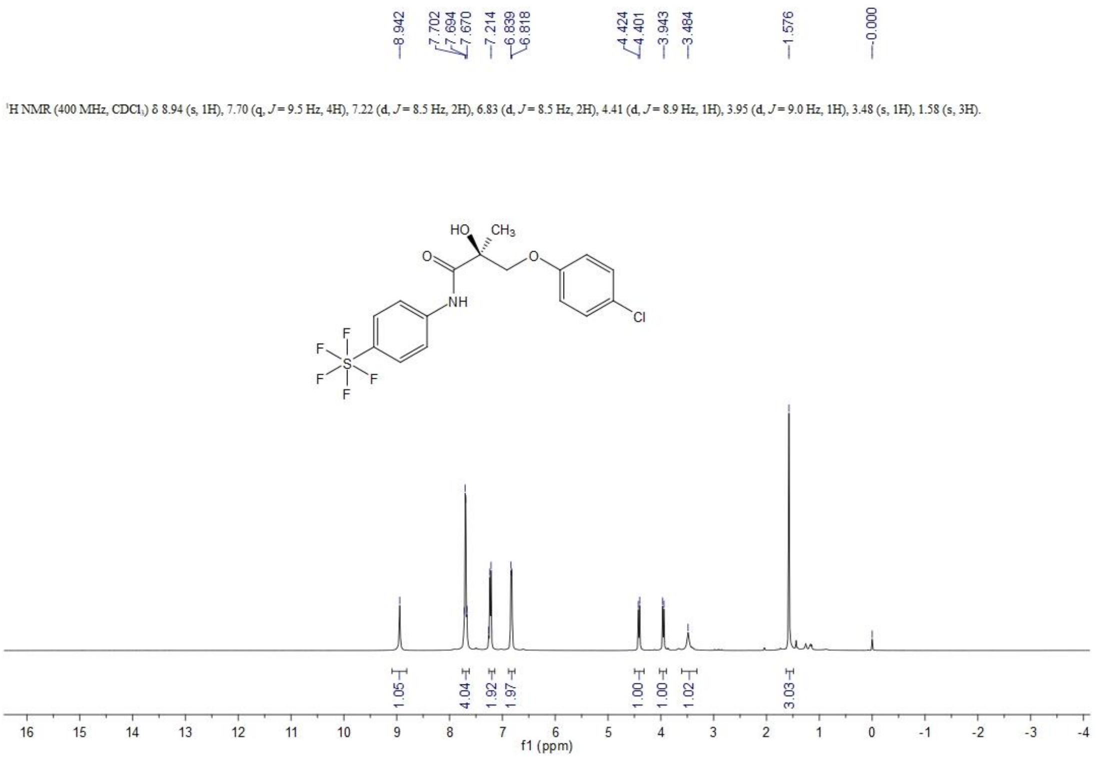

^{13}C NMR (101 MHz, $CDCl_3$) δ 172.25, 156.41, 149.45, 139.85, 129.54, 127.08, 126.91, 118.95, 116.08, 75.63, 72.81, 23.00

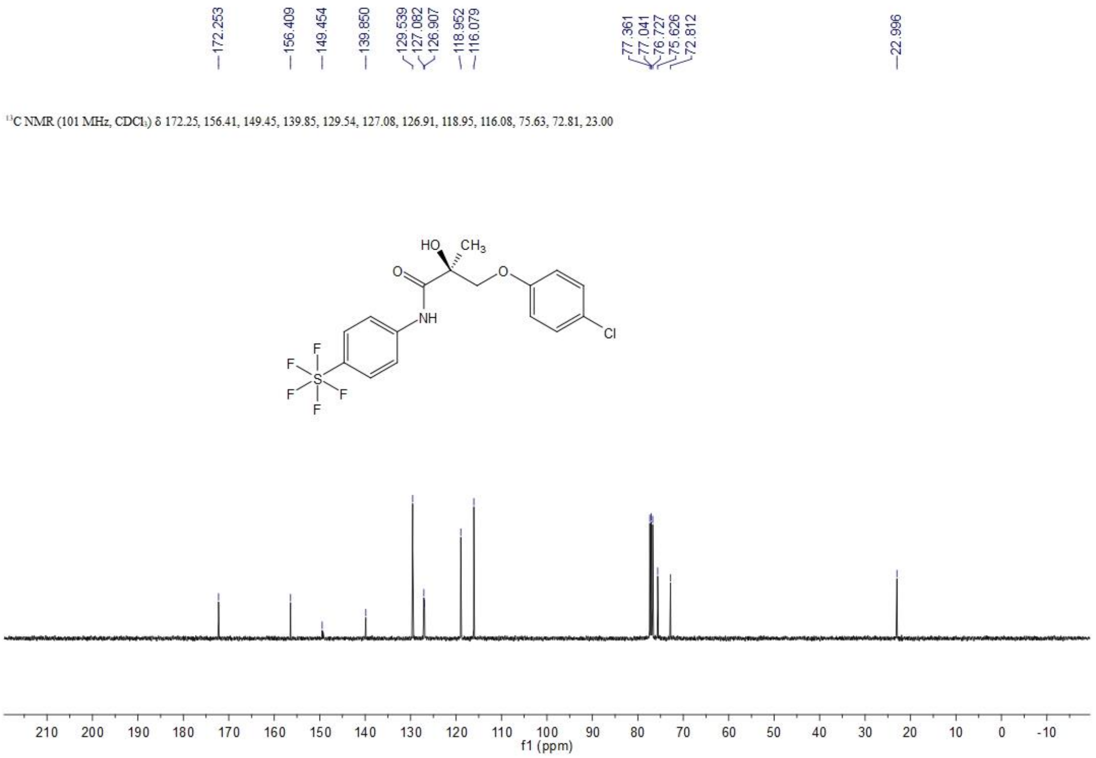

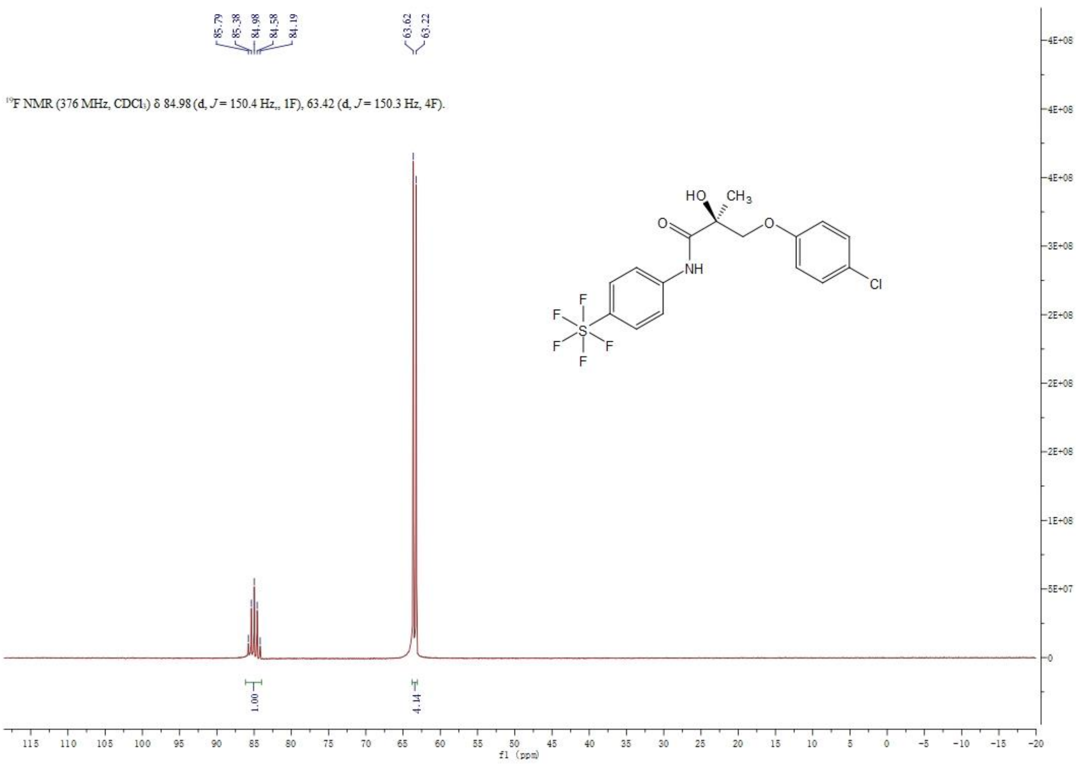

Elemental Composition Report **Page 1**

Single Mass Analysis
Tolerance = 100.0 PPM / DBE: min = -1.5, max = 50.0
Element prediction: Off

Monoisotopic Mass, Even Electron Ions
1 formula(e) evaluated with 1 results within limits (up to 50 closest results for each mass)
Elements Used:
C: 16-16 H: 16-16 N: 1-1 O: 3-3 F: 5-5 S: 1-1 Cl: 1-1
JJY-A00188-081 29 (0.588)
1: TOF MS ES+

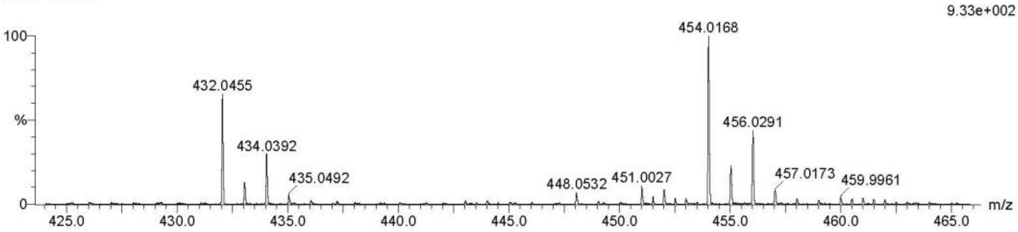

Minimum:				-1.5	
Maximum:		5.0	100.0	50.0	
Mass	Calc. Mass	mDa	PPM	DBE	Formula
432.0455	432.0460	-0.5	-1.2	6.5	C16 H16 N O3 F5 S Cl

Data File D:\AGILENT DATA\DATA\JJY\JJY20160203 2016-03-11 16-14-27\JJY0000001.D
Sample Name: JJY-A00188-081-1

===

Acq. Operator : JJY Seq. Line : 1
Acq. Instrument : Instrument 1 Location : Vial 81
Injection Date : 3/11/2016 4:15:59 PM Inj : 1
Inj Volume : 10.0 µl
Different Inj Volume from Sequence ! Actual Inj Volume : 15.0 µl
Acq. Method : D:\AGILENT DATA\DATA\JJY\JJY20160203 2016-03-11 16-14-27\JJY-0.1TFA-CH3CN-15MIN.M
Last changed : 3/11/2016 4:14:26 PM by JJY
Analysis Method : D:\AGILENT DATA\METHOD\XXY-PTX.M
Last changed : 11/6/2019 2:57:04 PM by HY
Additional Info : Peak(s) manually integrated

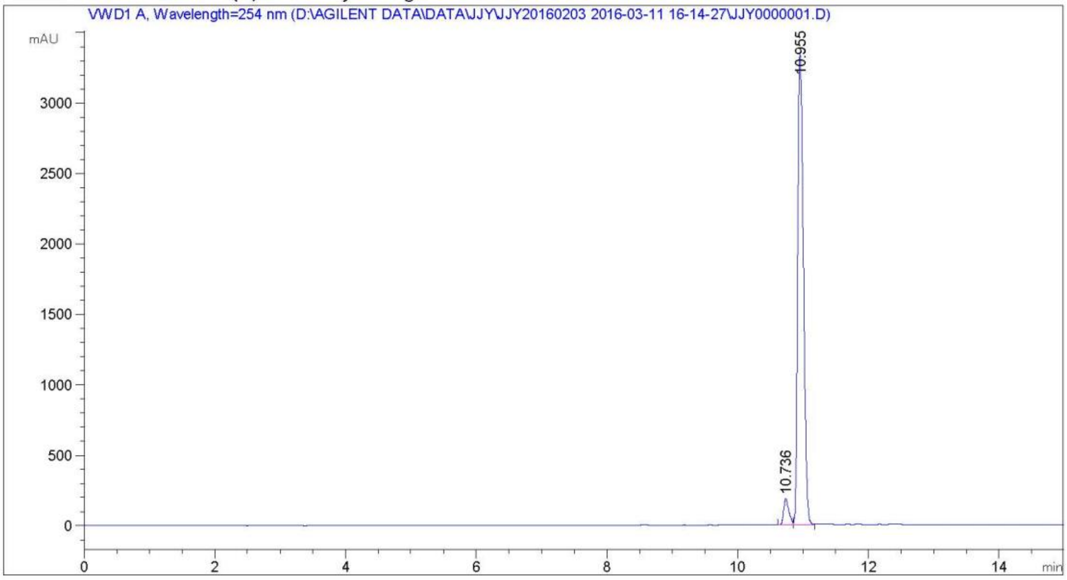

===

Area Percent Report

===

Sorted By : Signal
Multiplier : 1.0000
Dilution : 1.0000
Use Multiplier & Dilution Factor with ISTDs

Signal 1: VWD1 A, Wavelength=254 nm

Peak #	RetTime [min]	Type	Width [min]	Area mAU *s	Height [mAU]	Area %
1	10.736	BV	0.0820	1029.82776	186.29970	4.8199
2	10.955	VV	0.0957	2.03361e4	3341.78809	95.1801

Totals : 2.13660e4 3528.08778

^{1}H, ^{13}C, ^{19}F NMR, HPLC and HRMS spectra of *compound* **16d**

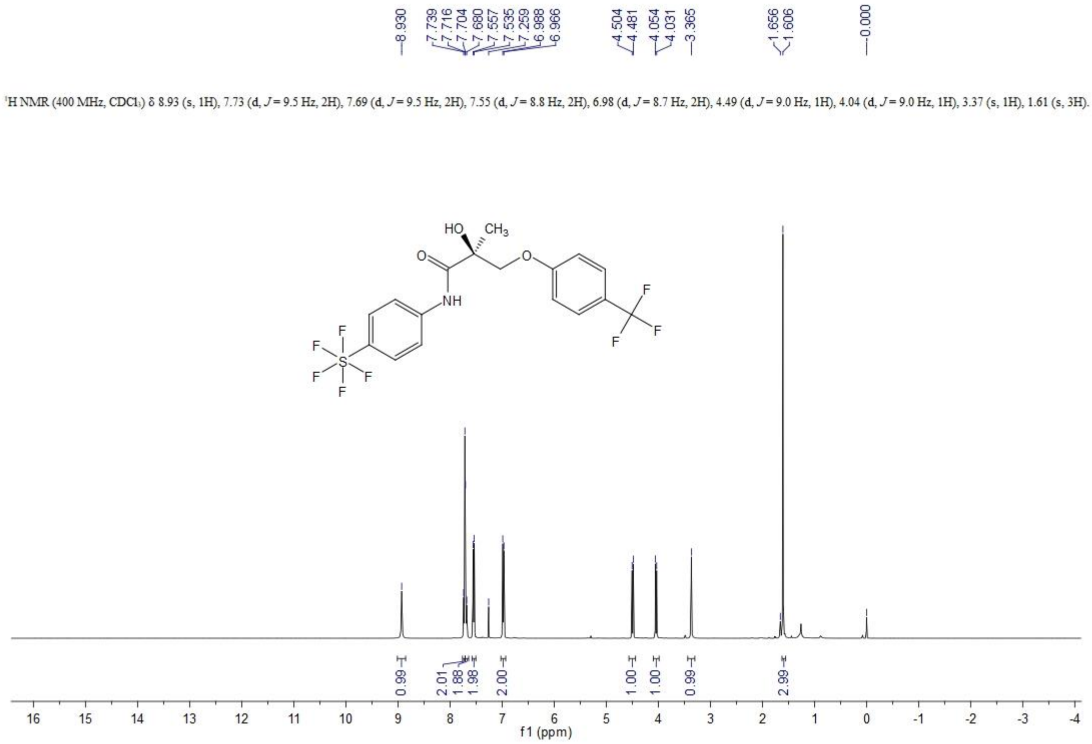

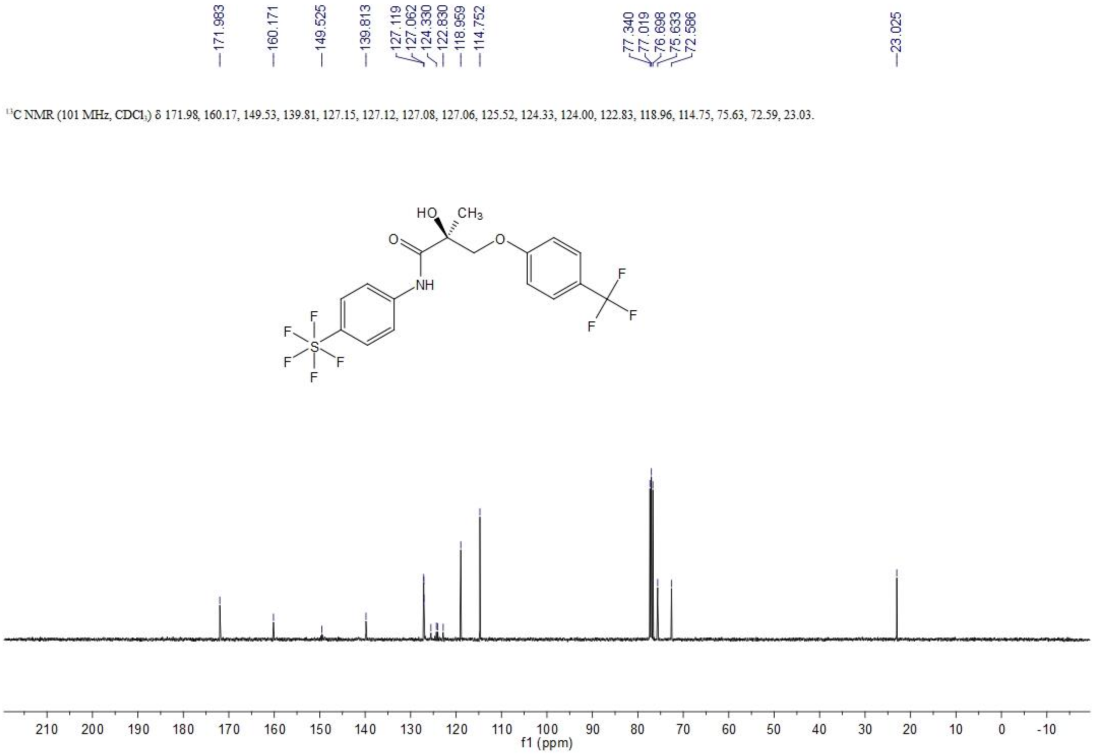

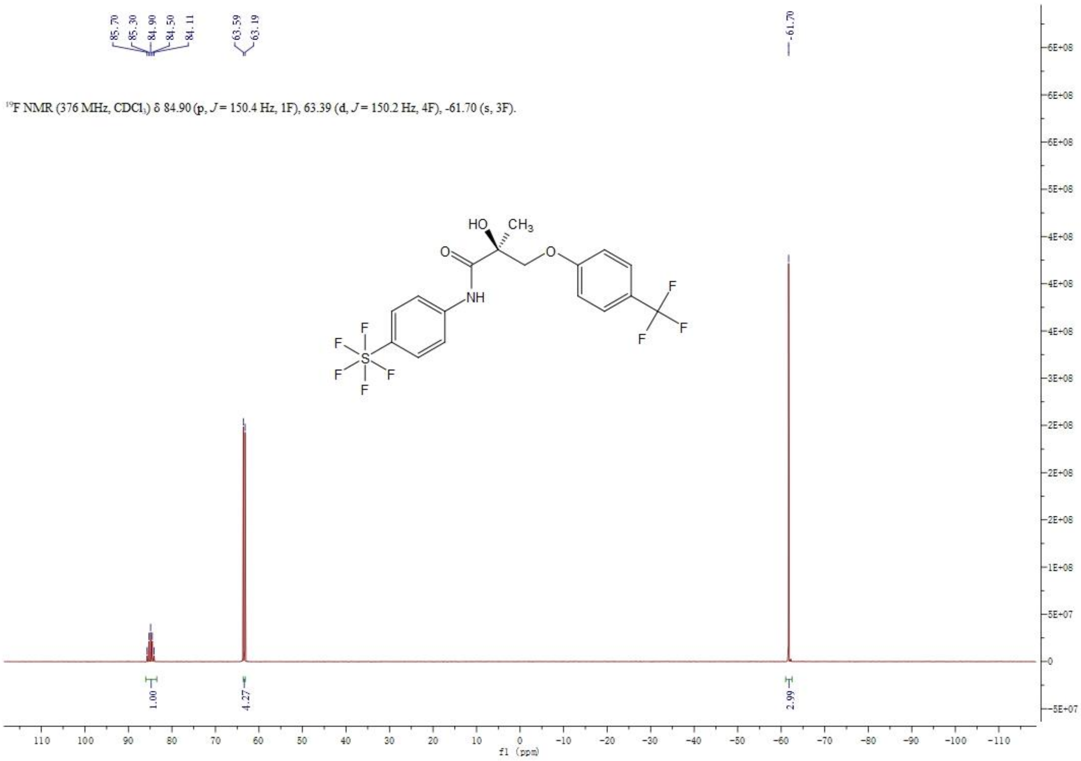

Elemental Composition Report **Page 1**

Single Mass Analysis
Tolerance = 100.0 PPM / DBE: min = -1.5, max = 50.0
Element prediction: Off

Monoisotopic Mass, Even Electron Ions
1 formula(e) evaluated with 1 results within limits (up to 50 closest results for each mass)
Elements Used:
C: 17-17 H: 15-15 N: 1-1 O: 3-3 F: 8-8 S: 1-1 Na: 0-1

JJY-A00188-085 31 (0.622)
1: TOF MS ES+

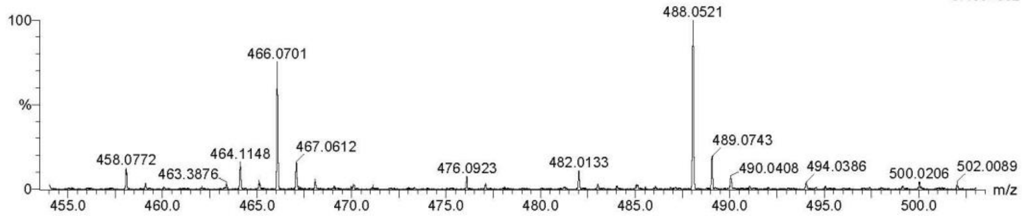

Minimum:				-1.5	
Maximum:		5.0	100.0	50.0	
Mass	Calc. Mass	mDa	PPM	DBE	Formula
488.0521	488.0543	-2.2	-4.5	6.5	C17 H15 N O3 F8 S Na

数据文件: C:\CHEM32\1\DATA\JJY\LW000039.D
样品名称: JJY-A00188-085

===

操作者 : spx
仪器 : 仪器 1 位置 : 样品瓶 1
进样日期 : 2019/11/14 16:57:04
进样量 : 没有进样
采集方法 : C:\CHEM32\1\METHODS\JJY-15MIN.M
最后修改 : 2019/11/14 16:54:16 : spx
(调用后修改)
分析方法 : C:\CHEM32\1\METHODS\JJY-15MIN.M
最后修改 : 2019/11/4 16:29:42 : CYT
附加信息: 峰已手动积分

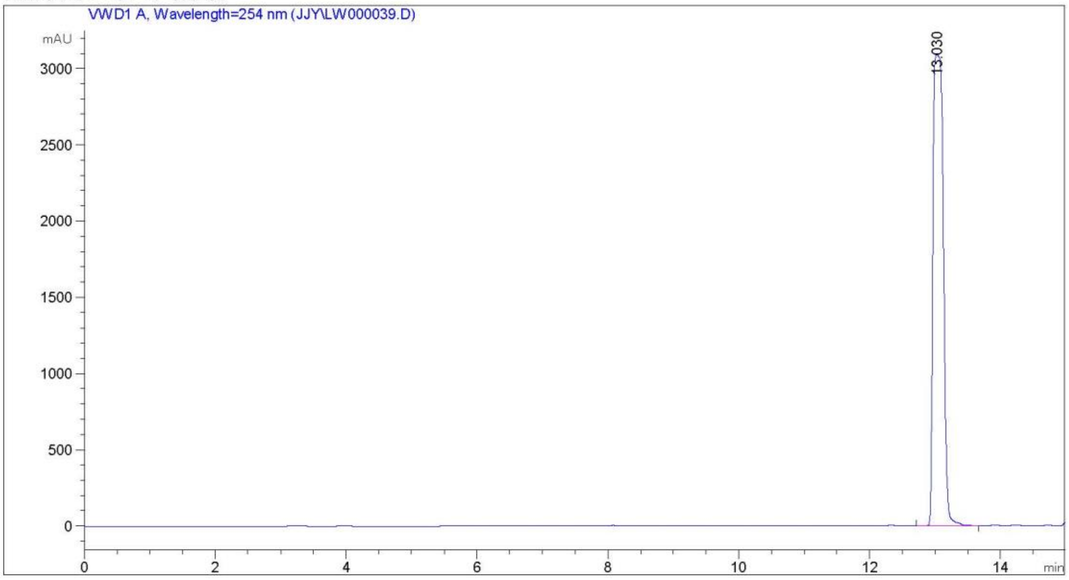

===
面积百分比报告
===

排序 : 信号
乘积因子: : 1.0000
稀释因子: : 1.0000
内标使用乘积因子和稀释因子

信号 1: VWD1 A, Wavelength=254 nm

峰 #	保留时间 [min]	类型	峰宽 [min]	峰面积 [mAU*s]	峰高 [mAU]	峰面积 %
1	13.030	VB	0.1758	3.34704e4	3092.09277	100.0000

总量 : 3.34704e4 3092.09277

===
*** 报告结束 ***

^{1}H, ^{13}C, ^{19}F NMR, HPLC and HRMS spectra of *compound* **16e**

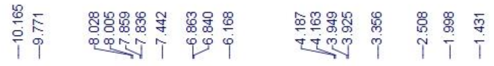

^{1}H NMR (400 MHz, DMSO) δ 10.17 (s, 1H), 9.77 (s, 1H), 8.02 (d, J = 8.9 Hz, 2H), 7.85 (d, J = 9.2 Hz, 2H), 7.45 (d, J = 8.9 Hz, 2H), 6.85 (d, J = 8.9 Hz, 2H), 6.17 (s, 1H), 4.17 (d, J = 9.5 Hz, 1H), 3.94 (d, J = 9.5 Hz, 1H), 2.00 (s, 3H), 1.43 (s, 3H).

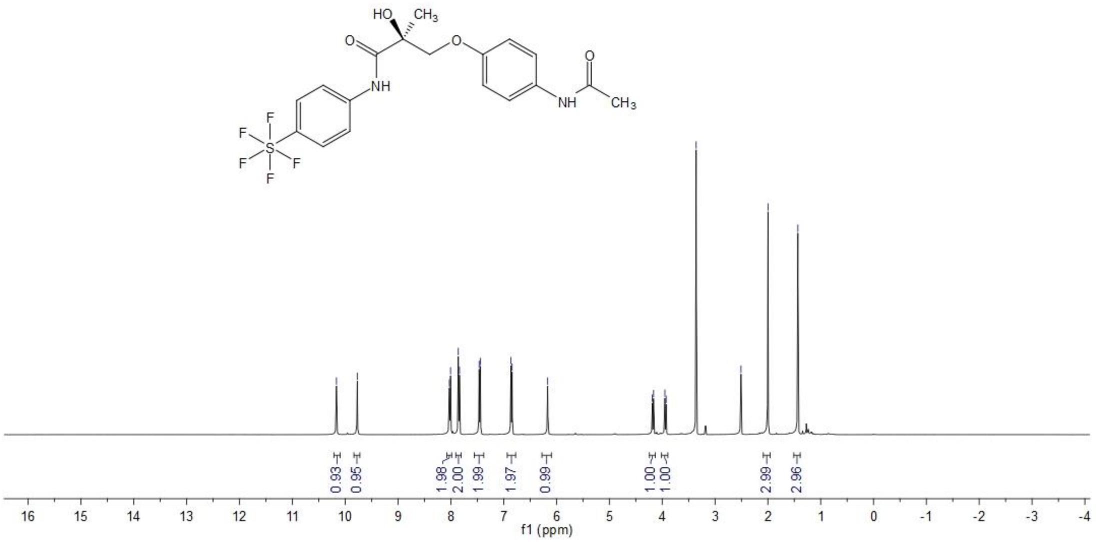

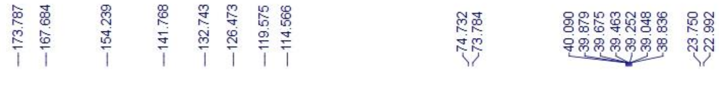

^{13}C NMR (101 MHz, DMSO) δ 173.79, 167.68, 154.24, 141.77, 132.74, 126.47, 120.36, 119.57, 114.57, 74.73, 73.78, 23.75, 22.99.

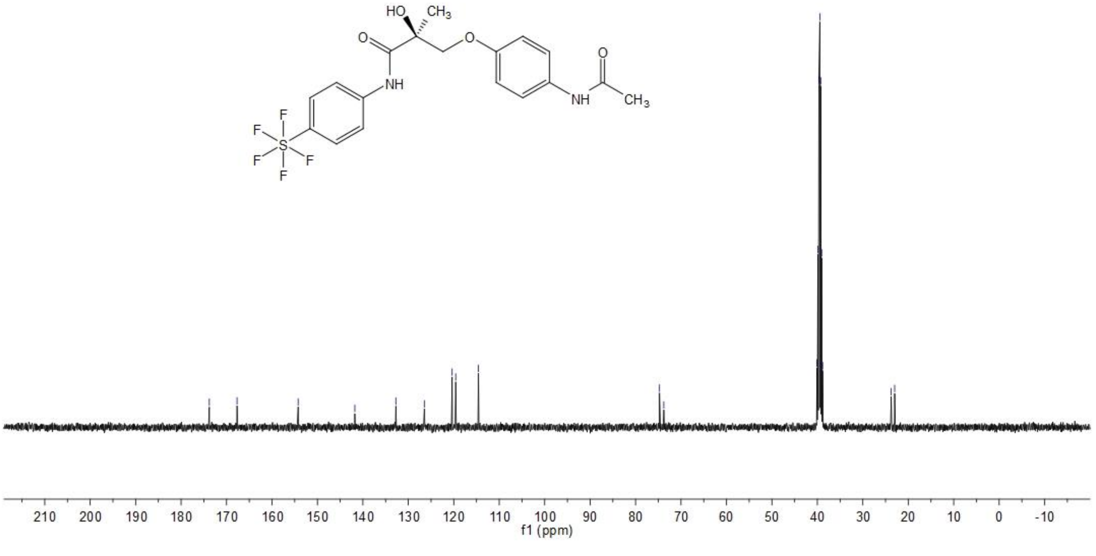

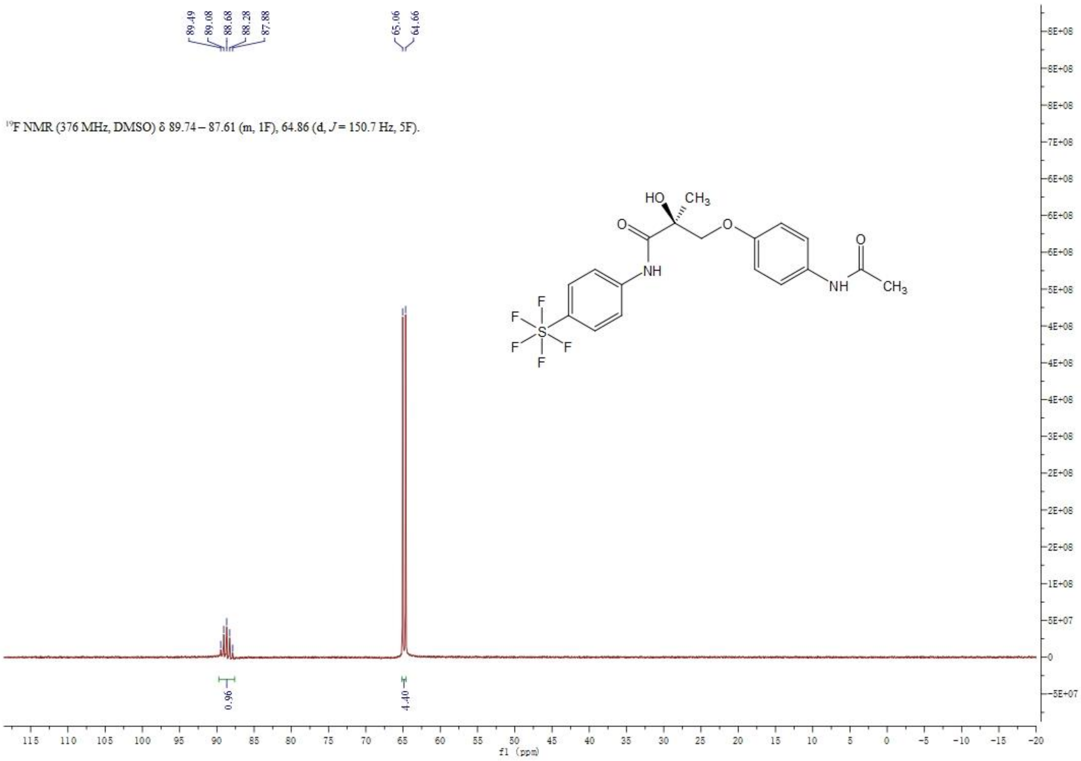

Elemental Composition Report **Page 1**

Single Mass Analysis
Tolerance = 100.0 PPM / DBE: min = -1.5, max = 50.0
Element prediction: Off

Monoisotopic Mass, Even Electron Ions
1 formula(e) evaluated with 1 results within limits (up to 50 closest results for each mass)
Elements Used:
C: 18-18 H: 19-20 N: 2-2 O: 4-4 F: 5-5 S: 1-1 Na: 0-1

JJY-A00188-083 60 (1.194)
1: TOF MS ES+

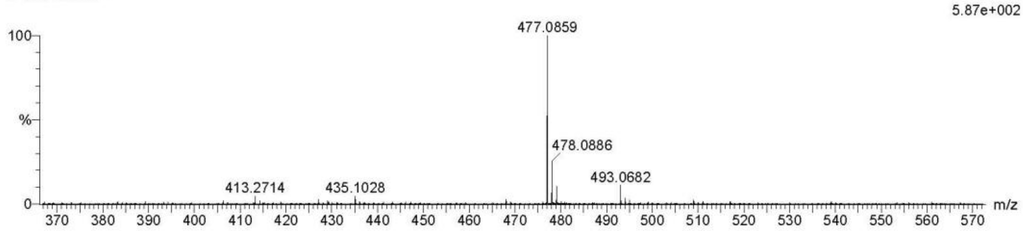

Minimum:				-1.5	
Maximum:		5.0	100.0	50.0	
Mass	Calc. Mass	mDa	PPM	DBE	Formula
477.0859	477.0883	-2.4	-5.0	7.5	C18 H19 N2 O4 F5 S Na

Data File D:\AGILENT DATA\DATA\JJY\JJY20160203 2016-03-12 13-53-14\JJY0000002.D
Sample Name: JJY-A00188-083

===

Acq. Operator : JJY Seq. Line : 2
Acq. Instrument : Instrument 1 Location : Vial 82
Injection Date : 3/12/2016 2:16:01 PM Inj : 1
Inj Volume : 10.0 µl
Different Inj Volume from Sequence ! Actual Inj Volume : 3.0 µl
Acq. Method : D:\AGILENT DATA\DATA\JJY\JJY20160203 2016-03-12 13-53-14\JJY-0.1TFA-CH3CN-15MIN.M
Last changed : 3/11/2016 4:14:26 PM by JJY
Analysis Method : D:\AGILENT DATA\METHOD\JJY-0.1TFA-CH3CN-15MIN.M
Last changed : 3/11/2016 4:14:26 PM by JJY
Additional Info : Peak(s) manually integrated

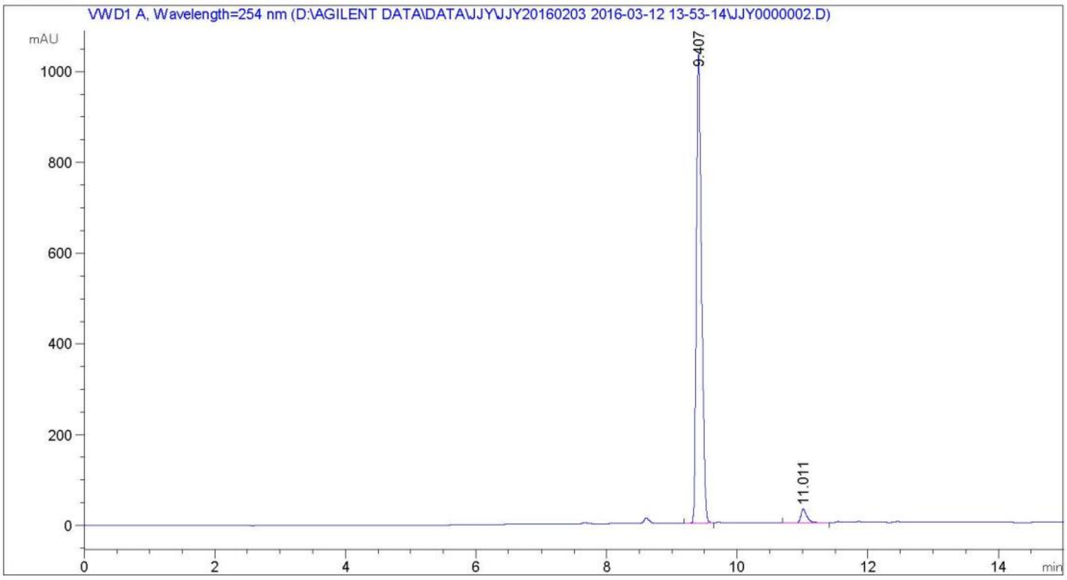

===

Area Percent Report

===

Sorted By : Signal
Multiplier : 1.0000
Dilution : 1.0000
Use Multiplier & Dilution Factor with ISTDs

Signal 1: VWD1 A, Wavelength=254 nm

Peak #	RetTime [min]	Type	Width [min]	Area mAU *s	Height [mAU]	Area %
1	9.407	BV	0.0827	5656.05322	1034.53369	96.7496
2	11.011	BB	0.0904	190.02280	30.41092	3.2504

Totals : 5846.07602 1064.94461

^{1}H, ^{13}C, ^{19}F NMR, HPLC and HRMS spectra of *compound* **16f**

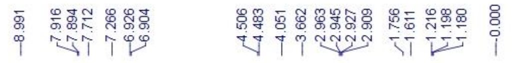

^{1}H NMR (400 MHz, $CDCl_3$) δ 8.99 (s, 1H), 7.91 (d, J = 8.8 Hz, 2H), 7.71 (s, 4H), 6.91 (d, J = 8.8 Hz, 2H), 4.49 (d, J = 9.2 Hz, 1H), 4.06 (d, J = 9.2 Hz, 1H), 3.66 (s, 1H), 2.94 (q, J = 7.2 Hz, 2H), 1.61 (s, 3H), 1.20 (t, J = 7.2 Hz, 3H).

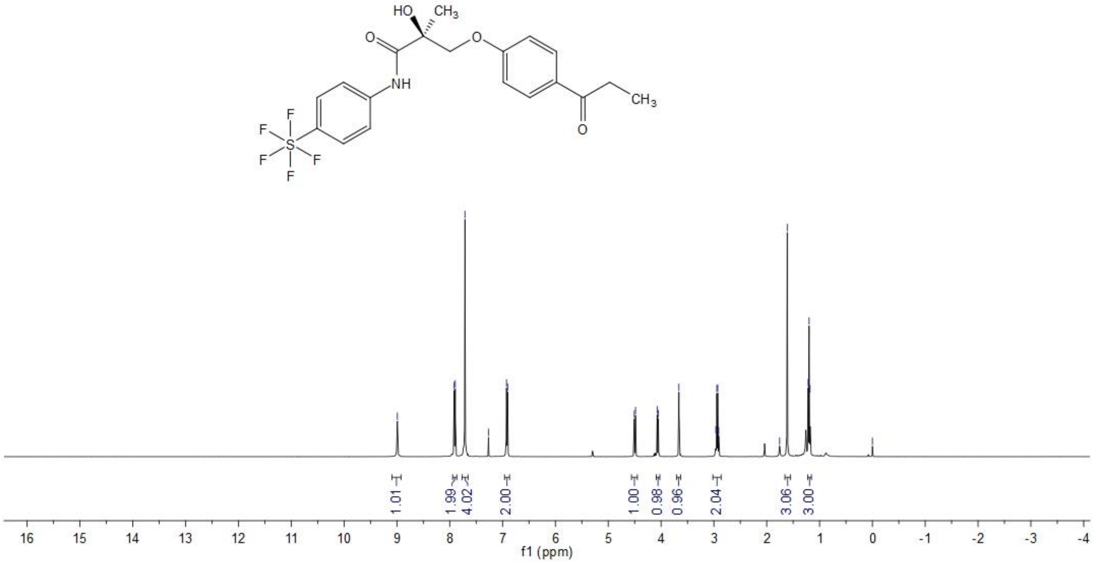

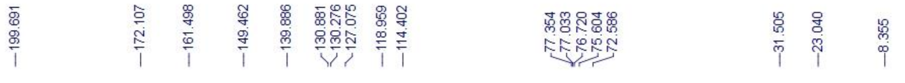

^{13}C NMR (101 MHz, $CDCl_3$) δ 199.69, 172.11, 161.50, 149.46, 139.89, 130.88, 130.28, 127.07, 118.96, 114.40, 75.60, 72.59, 31.51, 23.04, 8.35.

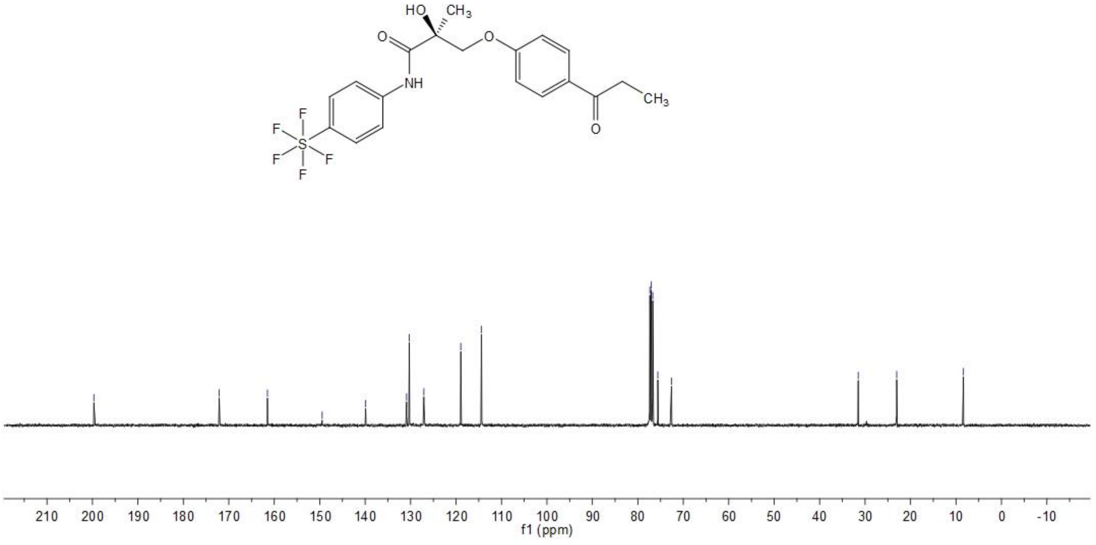

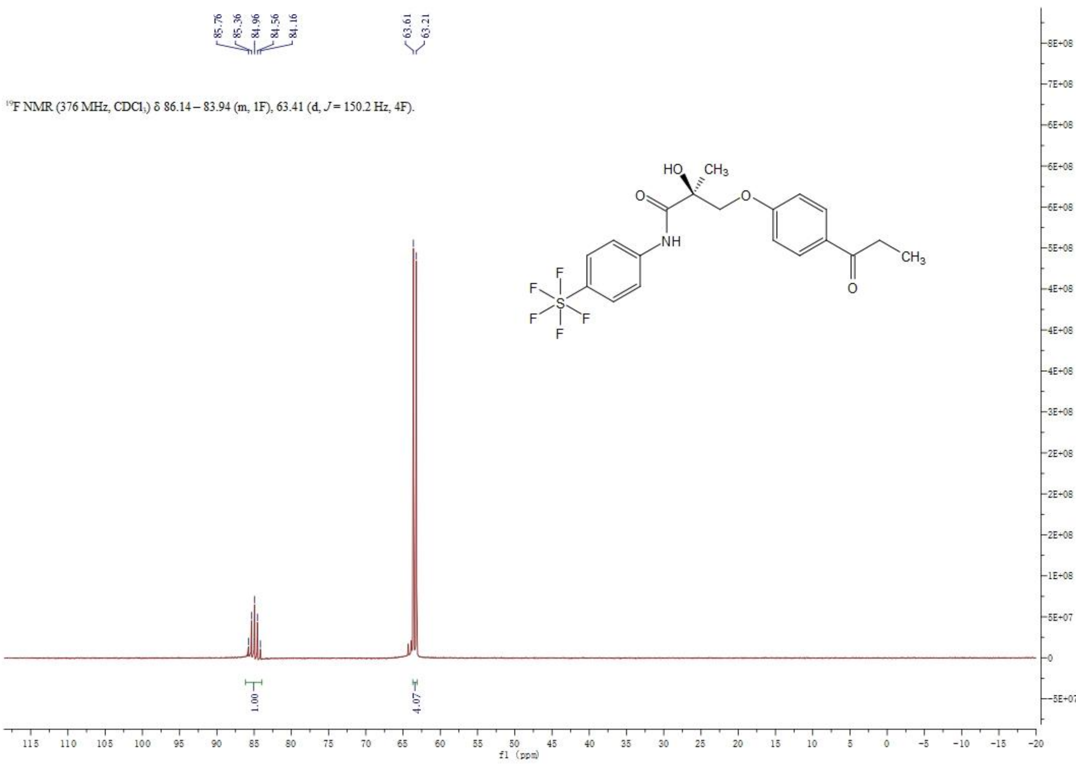

Elemental Composition Report **Page 1**

Single Mass Analysis
Tolerance = 100.0 PPM / DBE: min = -1.5, max = 50.0
Element prediction: Off

Monoisotopic Mass, Even Electron Ions
1 formula(e) evaluated with 1 results within limits (up to 50 closest results for each mass)
Elements Used:
C: 19-19 H: 20-20 N: 1-1 O: 4-4 F: 5-5 Na: 0-1 S: 1-1
JJY-A00188-084 27 (0.554)
1: TOF MS ES+

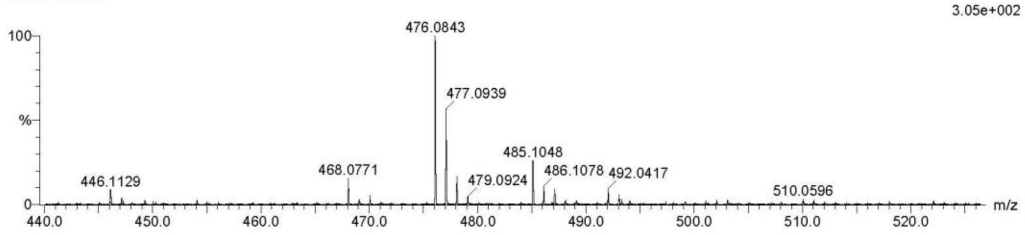

Minimum:				-1.5	
Maximum:		5.0	100.0	50.0	
Mass	Calc. Mass	mDa	PPM	DBE	Formula
476.0923	476.0931	-0.8	-1.7	7.5	C19 H20 N O4 F5 Na S

Data File D:\AGILENT DATA\DATA\JJY\JJY20160203 2016-03-15 13-28-32\JJY0000001.D
Sample Name: JJY-A00188-084

===

Acq. Operator : JJY Seq. Line : 1
Acq. Instrument : Instrument 1 Location : Vial 81
Injection Date : 3/15/2016 1:30:01 PM Inj : 1
Inj Volume : 10.0 µl
Different Inj Volume from Sequence ! Actual Inj Volume : 5.0 µl
Acq. Method : D:\AGILENT DATA\DATA\JJY\JJY20160203 2016-03-15 13-28-32\JJY-0.1TFA-CH3CN-15MIN.M
Last changed : 3/14/2016 12:04:47 PM by JJY
Analysis Method : D:\AGILENT DATA\METHOD\JJY-0.1TFA-CH3CN-15MIN.M
Last changed : 3/14/2016 12:04:47 PM by JJY
Additional Info : Peak(s) manually integrated

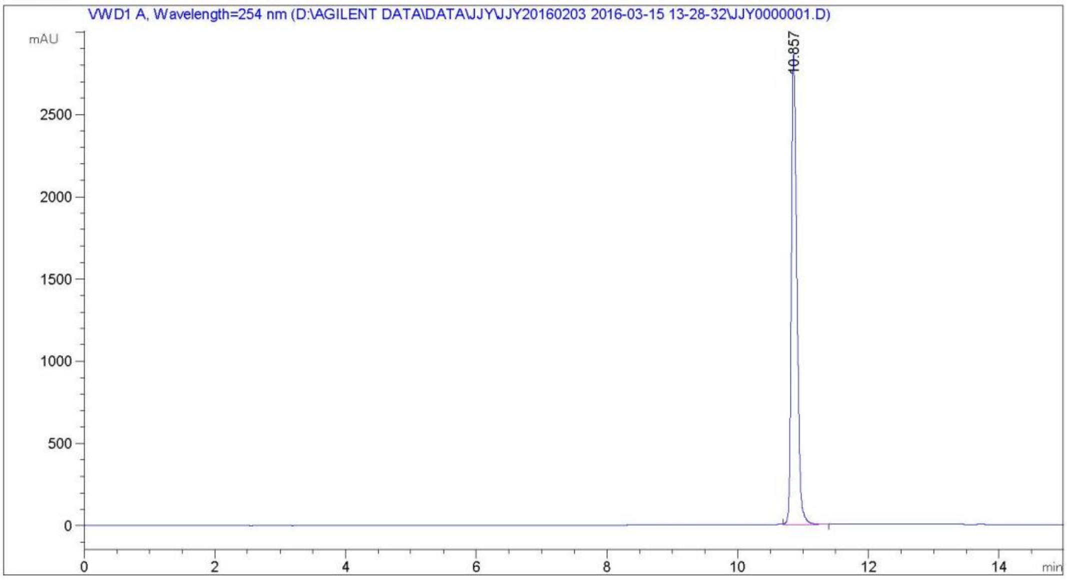

===

Area Percent Report

===

Sorted By : Signal
Multiplier : 1.0000
Dilution : 1.0000
Use Multiplier & Dilution Factor with ISTDs

Signal 1: VWD1 A, Wavelength=254 nm

Peak #	RetTime [min]	Type	Width [min]	Area mAU *s	Height [mAU]	Area %
1	10.857	VB	0.0870	1.66829e4	2860.37720	100.0000

Totals : 1.66829e4 2860.37720

===

^{1}H, ^{13}C, ^{19}F NMR, HPLC and HRMS spectra of *compound* **16g**

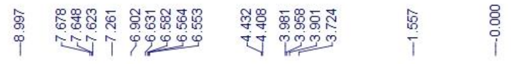

^{1}H NMR (400 MHz, $CDCl_3$) δ 9.00 (s, 1H), 7.76 – 7.62 (m, 4H), 6.92 (t, J = 9.2 Hz, 1H), 6.64 (dd, J = 12.7, 2.9 Hz, 1H), 6.57 (ddd, J = 9.0, 2.9, 1.4 Hz, 1H), 4.42 (d, J = 9.3 Hz, 1H), 3.97 (d, J = 9.2 Hz, 1H), 3.90 (s, 1H), 3.72 (s, 3H), 1.56 (s, 3H).

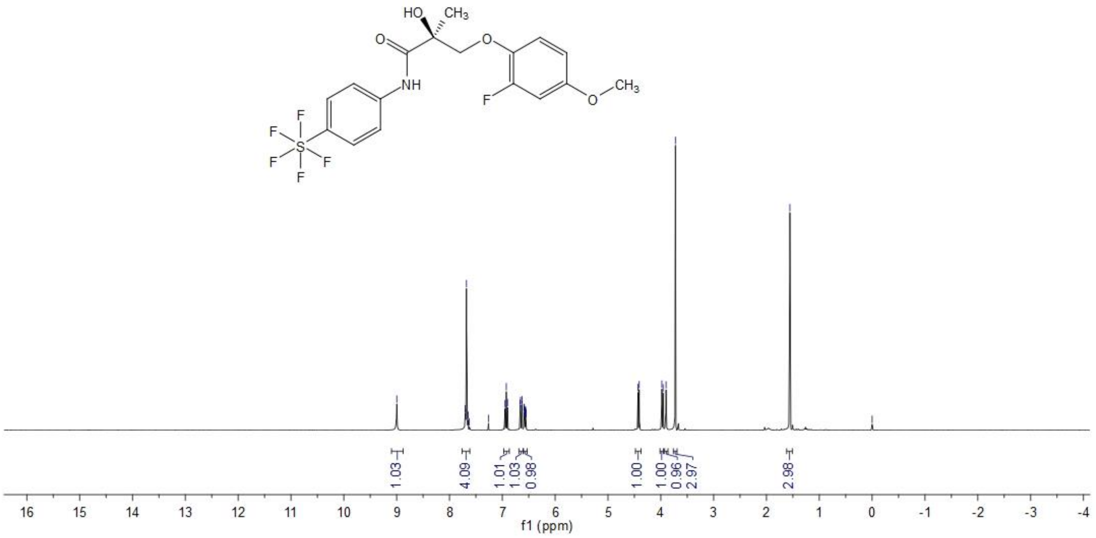

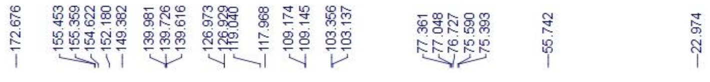

^{13}C NMR (101 MHz, $CDCl_3$) δ 172.68, 155.45, 155.36, 154.62, 152.18, 149.38, 139.98, 139.73, 139.62, 127.02, 126.97, 126.93, 119.04, 117.99, 117.97, 109.17, 109.15, 103.36, 103.14, 75.59, 75.39, 55.74, 22.97.

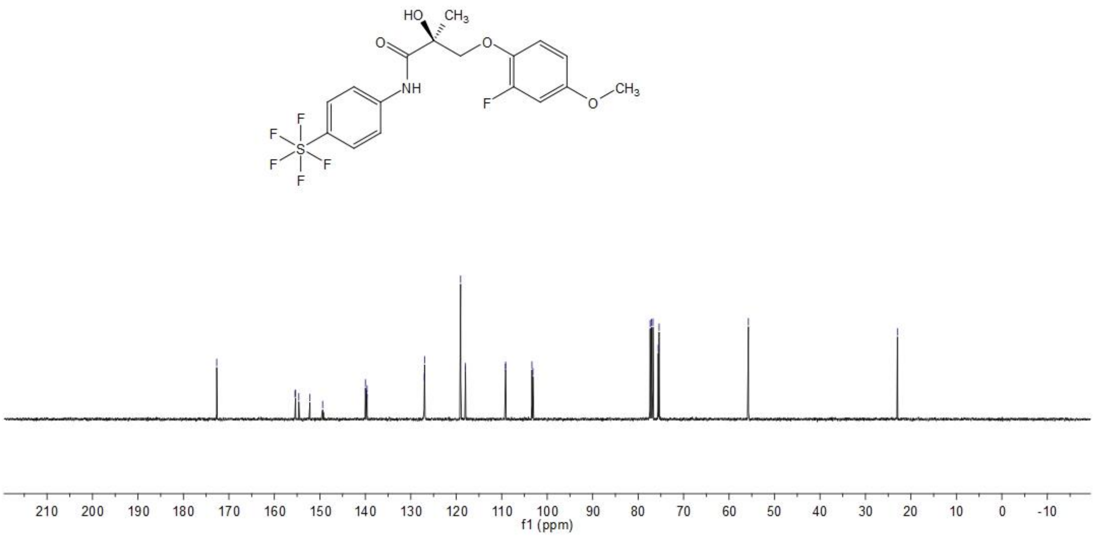

^{19}F NMR (376 MHz, $CDCl_3$) δ 86.57 – 84.10 (m, 1F), 63.39 (d, J = 150.3 Hz, 3F), -131.31 (s, 1F).

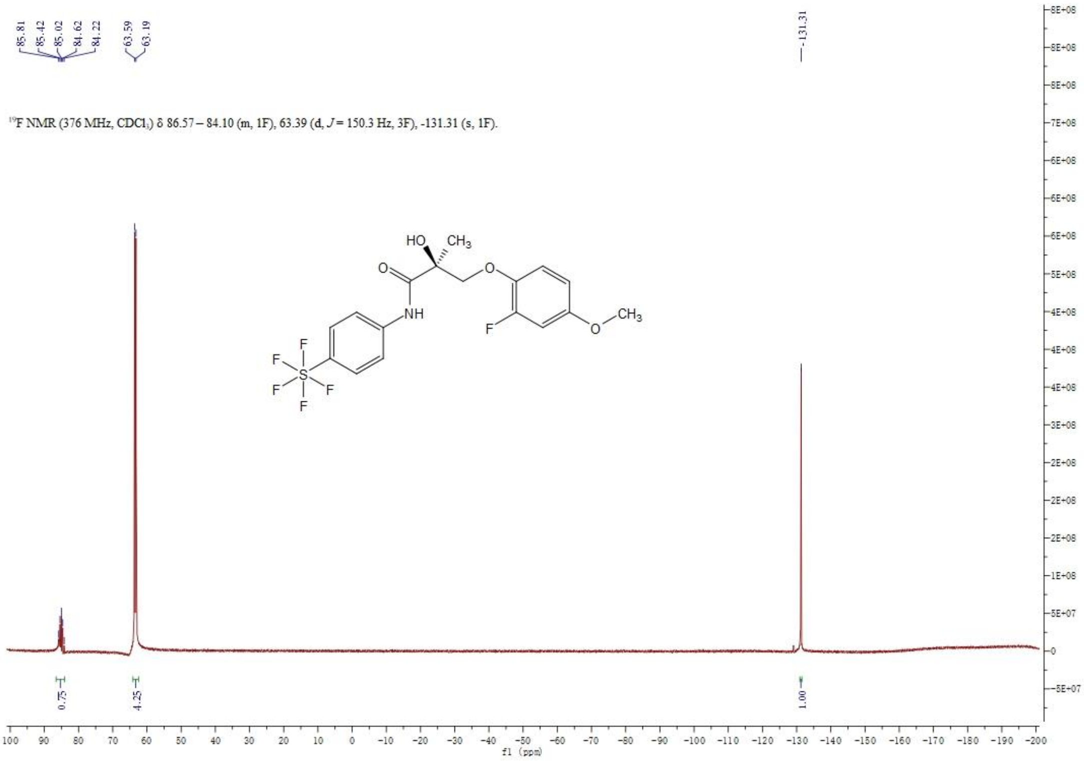

Elemental Composition Report **Page 1**

Single Mass Analysis
Tolerance = 100.0 PPM / DBE: min = -1.5, max = 50.0
Element prediction: Off

Monoisotopic Mass, Even Electron Ions
1 formula(e) evaluated with 1 results within limits (up to 50 closest results for each mass)
Elements Used:
C: 17-17 H: 18-18 N: 1-1 O: 4-4 F: 6-6 S: 1-1
JJY-A00188-089 33 (0.676) Cm (33:42)
1: TOF MS ES+

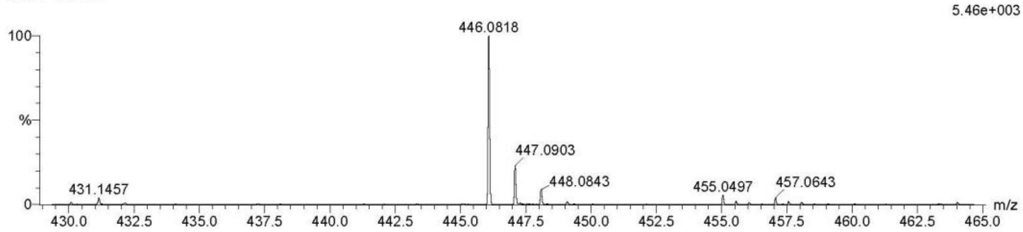

Minimum:				-1.5	
Maximum:		5.0	100.0	50.0	
Mass	Calc. Mass	mDa	PPM	DBE	Formula
446.0818	446.0861	-4.3	-9.6	6.5	C17 H18 N O4 F6 S

Data File D:\AGILENT DATA\DATA\JJY\JJY20160203 2016-03-17 13-17-41\JJY0000001.D
Sample Name: JJY-A00188-089

===

Acq. Operator : JJY   Seq. Line : 1
Acq. Instrument : Instrument 1   Location : Vial 81
Injection Date : 3/17/2016 1:19:11 PM   Inj : 1
                                                  Inj Volume : 10.0 µl
Acq. Method : D:\AGILENT DATA\DATA\JJY\JJY20160203 2016-03-17 13-17-41\JJY-0.1TFA-CH3CN-15MIN.M
Last changed : 3/17/2016 12:29:03 PM by JJY
Analysis Method : D:\AGILENT DATA\METHOD\JJY-0.1TFA-CH3CN-15MIN.M
Last changed : 3/17/2016 12:29:03 PM by JJY
Additional Info : Peak(s) manually integrated

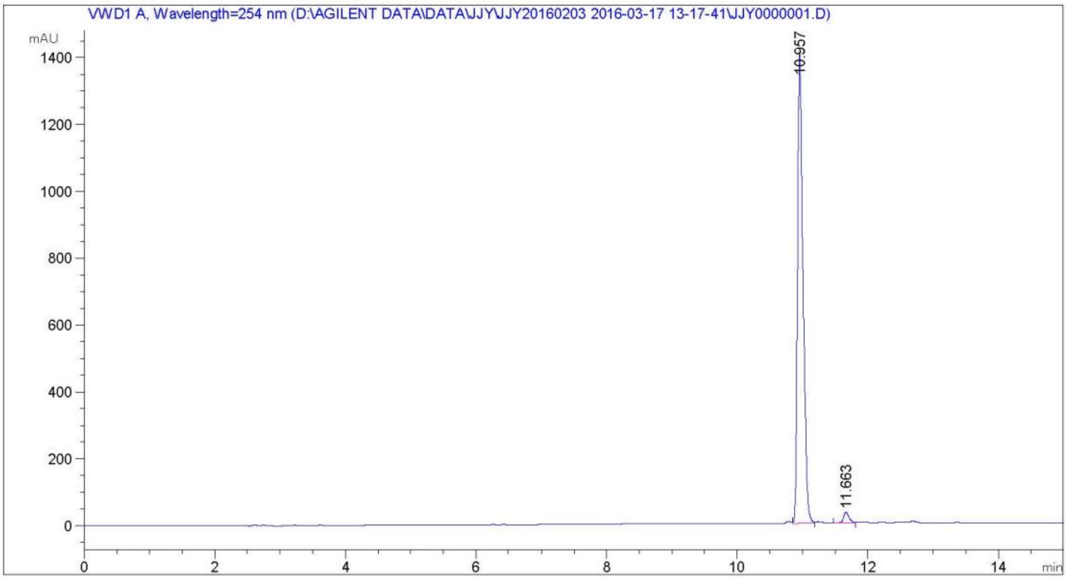

===

Area Percent Report

===

Sorted By : Signal
Multiplier : 1.0000
Dilution : 1.0000
Use Multiplier & Dilution Factor with ISTDs

Signal 1: VWD1 A, Wavelength=254 nm

Peak #	RetTime [min]	Type	Width [min]	Area mAU *s	Height [mAU]	Area %
1	10.957	VV	0.0834	8127.12256	1406.40491	97.8307
2	11.663	BV	0.0823	180.21136	32.43166	2.1693

Totals : 8307.33392 1438.83657

===

Instrument 1 3/17/2016 2:10:45 PM JJY   Page 1 of 1
